# Supplementary material for: Medication Reconciliation: An Educational Module
Source: MedEdPORTAL. 2019 Nov 1;15:10852. doi: 10.15766/mep_2374-8265.10852 (PMC6952281; doi:10.15766/mep_2374-8265.10852)
Supplement: Supplementary file 1 — A. Medication Reconciliation Slides.pptx B. Embedded ARS Questions.docx C. Pre-Post Assessment.docx D. Pre-Post Assessment Answers and References.docx [file mep-15-10852-s001.zip › _Educational_Summary_Report_10852.pdf]

MedEdPORTAL® The AAMC Journal of Teaching and Learning Resources

Original Publication

Open Access

Medication Reconciliation: An Educational Module

Paula E. Lester, MD*, Sukhminder Sahansra, MD, Mark Shen, PharmD, Maria Becker, DO, Shahidul Islam, DrPH

*Corresponding author: plester@winthrop.org

Abstract

Introduction: Patients often transition between health care settings, such as office to hospital, hospital to nursing facility, or hospital to home. When a patient is admitted, it is imperative that clinicians review prior medication lists along with new orders to reconcile any discrepancies. This process should occur in a standardized manner to reduce medication errors leading to adverse events and patient harm. **Methods:** We developed this program as an instructional method via PowerPoint to teach the importance of accurate medication reconciliation. We implemented the program in multiple grand rounds settings with students, trainees, and attending physicians in internal medicine and surgery. Approximately 150 learners attended the sessions. We assessed learners with pre/post self-efficacy assessment (74 completed precourse surveys, 39 completed posttest surveys, and 49 participated in the audience response during the course) and multiple-choice knowledge questions. **Results:** The results of the postcourse knowledge assessment demonstrated improvement in every question we tested, with two of the improvements reaching statistical significance. We found that 30% of attendees were not at all confident or only somewhat confident in conducting an appropriate medication reconciliation on admission to the hospital. Additionally, 82% of respondents reported that the presentation was likely or extremely likely to improve their medication reconciliation efforts. **Discussion:** Our educational program was successful in improving learners' knowledge in every question we tested; however, only two of the improvements were statistically significant. Our program is an organized and effective tool for teaching effective and reliable medication reconciliation.

Keywords
Medication Reconciliation, Transitions of Care, Transitional Care, Hospitalization, Postacute Care, Subacute Care

Educational Objectives

By the end of this learning session, participants will be able to:

1. Define medication reconciliation and discrepancies.
2. Demonstrate case examples.
3. Construct a best possible medication history.
4. Describe one example of hospital committee interventions.
5. Describe practice recommendations from the Joint Commission.

Introduction

Medication reconciliation is defined by the Institute for Healthcare Improvement as the process of creating the most accurate list possible of all medications a patient is taking—including the drug name, dosage, frequency, and route—and comparing that list against the physician's admission, transfer, and/or discharge orders, with the goal of providing correct medications to the patient at all transition points within the hospital.[1,2]

Citation:
Lester PE, Sahansra S, Shen M, Becker M, Islam S. Medication reconciliation: an educational module. *MedEdPORTAL*. 2019;15:10852. https://doi.org/10.15766/mep_2374-8265.10852

Research has demonstrated that inaccurate communication of medical information at transitions of care is responsible for as many as 50% of all medication errors and up to 20% of adverse drug events in the hospital setting.[3] Accurate and reliable medication reconciliation at all transitions of care helps prevent adverse drug events and avoid harm to patients.

When a patient is admitted to a new health care venue (e.g., hospital or nursing facility), it is imperative that clinicians review the prior medication list along with new orders and plans for care and reconcile any discrepancies. Information needed in an accurate medication reconciliation process includes the drug name, dosage, frequency, and route. Accurate and reliable medication reconciliation at all transitions of care helps prevent adverse drug events.[4] The medication reconciliation process should be standardized to ensure comprehensive reconciliation to reduce medication errors that can lead to patient harm. Discharge from the hospital is also an essential time for accurate medication reconciliation, as medications might have been

Copyright © 2019 Lester et al. This is an open-access publication distributed under the terms of the Creative Commons Attribution license.

added, changed, held, or discontinued in the hospital for a variety of reasons, such as newly diagnosed medical conditions, drug interactions, or complying with the hospital formulary.[5] Based on all of the changes in the hospital, discharge medications should be reconciled appropriately.

Medication reconciliation begins with collecting a current medication list. The medication list should include all medications (prescriptions, over-the-counter, herbals, supplements, etc.) and their dose, route, frequency, and indication. It is vital to know which medications the patient has been taking. It is also necessary to validate if the patient is actually taking the medication as prescribed to ensure that the patient is not under- or overdosed. Subsequently, decisions must be made as to whether the medication should be continued, changed, or discontinued. Patients and health care providers providing subsequent care for patients need to be informed of these changes.

Recognizing the importance of reliable medication reconciliation has led to varied institutional efforts to improve awareness and accuracy about the process. Prior research on educational modules has focused only on pharmacy students or internal medicine residents.[6,7] Our educational module on medication reconciliation adds to the current literature because, in addition to including effective materials for both training and assessment, our program is used for multiple learner levels (trainees, mid-level providers, and attending physicians) and for both internal medicine and surgery clinicians. Additionally, we provide several methods for assessment of learners.

In our institution, we implemented this course in two large-group settings in an internal medicine grand rounds and a surgery grand rounds for faculty and house staff. We also conducted the course for physician assistant/nurse practitioner (PA/NP) grand rounds, which was a smaller group setting. These fields were selected because they have a high ratio of sick patients on multiple medications being admitted; in addition, many of their patients are discharged to a skilled nursing facility. We chose to implement our educational model in a large-group grand rounds setting because of the greater efficiency in reaching a critical number of attendees in one setting. Although smaller groups may be amenable to more discussion of medication reconciliation errors and near misses, the topic of medication reconciliation is not one that requires debate or focused deliberation to learn the material and concepts. This presentation was successful in our institution when implemented for trainees, mid-level providers, and attending physicians. We believed that it was imperative to include faculty in addition to trainees for a multitude of reasons: Faculty should be directing the overall admission and discharge process, they are encouraged to verify the medication reconciliation, and if they are not cognizant of the importance of medication reconciliation, then they will not reinforce the concept to trainees and mid-level providers.

Methods

Health care institutions have increasingly recognized the importance of accurate medication reconciliation, and organizations such as the Institute for Healthcare Improvement and the Joint Commission also have been emphasizing the importance of medication reconciliation during transitions of care.

Our institution developed the Medication Reconciliation Committee in an effort to assess current practices, to conduct a needs assessment, and to strategize to improve processes. We found that the medication reconciliation process was undervalued and underrecognized as a vital component of patient care. We also recognized technical barriers to performing medication reconciliation, including multiple electronic health records (EHRs) used in outpatient settings and nursing homes, with none of those EHRs communicating directly with the hospital EHR. The committee reviewed previous literature and curricula created to educate about medication reconciliation and developed an educational program for clinicians—the medication reconciliation educational program presented here. Our content experts on the committee developed a PowerPoint (PPT) educational slide deck, multiple-choice pre-/postassessment tool, and audience response questions embedded in the PPT.

The learning objectives for our educational program were determined by the membership of the Medication Reconciliation Committee. There was no prerequisite knowledge needed by learners. We chose clinicians with extensive experiences in transitioning patients to varied health settings to be facilitators for this course, but this is not required. We used small focus groups of geriatric medicine fellows, geriatric medicine attendings, and hospitalist attendings to evaluate the validity, feasibility, and utility of our content and to pilot our assessment tools, which were developed by content experts on the Medication Reconciliation Committee. Our grand rounds presentations were the first department-level educational method used for training in medication reconciliation.

To get our baseline assessment of knowledge and confidence, we distributed an online precourse survey through

Copyright © 2019 Lester et al. This is an open-access publication distributed under the terms of the Creative Commons Attribution license.

SurveyMonkey.com a week prior to each of the grand rounds presentations to all potential attendees of the grand rounds sessions. The oral presentation (using the PPT in Appendix A) took approximately 45 minutes and was presented by clinicians with transitions of care expertise: two geriatric medicine physicians, a surgery PA, and a PharmD. Our PPT presentation included case scenarios to help learners recognize the potential adverse outcomes from poor medication reconciliation in real-life examples. The Medication Reconciliation Committee received support from the broader Quality Improvement Committee to use this format. We used additional time within the 1-hour grand rounds time slot to include self-efficacy assessments (Appendix B) and the multiple-choice knowledge assessment tool (Appendix C). For attendees who did not submit their precourse assessment in the week prior to the presentation, we provided paper copies of the precourse assessment tool and collected them before the presentation began. Additionally, a posttest on knowledge was implemented and could be completed on paper or online using SurveyMonkey after the session.

We did not review the answers to the assessment tool in real time because we did not want to influence those who chose to take the posttest online. However, educators using this module could distribute the answer key (Appendix D) to each learner after completion of the knowledge assessment or review the key together as a group led by the preceptor. No additional materials are needed beyond the included resources; however, case presentations from individual institutions may be useful. Additional clinical scenarios submitted by the learners may be useful and meaningful for attendees, as they can learn how the presenter would reconcile a medication in a given scenario. This can be done through a submission process in advance of the presentation or ad hoc during the presentation.

We used an audience response system (ARS) called Poll Everywhere for self-efficacy embedded in the presentation. Our self-efficacy assessments (Appendix B) were implemented using the ARS; however, options include conducting them via paper or embedding them in the PPT as part of an ARS such as Poll Everywhere. We utilized these questions to help the learners assess their initial comfort level and encourage them to be more engaged during the course as they recognized weakness and strove to gain knowledge.

Implementation in other institutions can easily be adapted based on individual needs. The PPT educational presentation can be provided by providers familiar with the hospital discharge process. This medication reconciliation program can be provided in a large or small setting to improve knowledge about appropriate medication reconciliation in the hospital and other settings. Smaller groups of 12-15 learners may allow for greater discussion of patient cases in a workshop format. This educational module is focused and directed so learners can more accurately conduct medication reconciliation. Successful implementation can be increased by personalization based on individual institutional resources (e.g., contact information for pharmacists and quality assurance staff). Additionally, the educational material can include any documents or guidelines unique to one's own institution.

Practical implementation is straightforward. The knowledge-based multiple-choice assessment can be conducted only after the education program or can be conducted both before and after the education module. The multiple-choice assessment can be administered via paper or as an online test after the didactic session. Whether online or on paper, conducting the posttest within the session time is more likely to result in better response rates than expecting learners to find time after the session to complete the posttest. The knowledge assessments can also be conducted prior to the course to serve as a needs assessment and/or to provide baseline scores. Our 10-question assessment tool includes one demographics question, which other users of the program may choose to exclude. Follow-up assessments of self-efficacy and knowledge could be conducted in a reasonable follow-up period (e.g., 3 months) to evaluate for knowledge retention.

The files included in the submission are as follows:

- Medication reconciliation PPT slides (Appendix A)—to be used in presenting the importance of appropriate medication reconciliation and keys to effective transitions of care. The slides include descriptive text and additional information in the Notes section.
- Questions on self-efficacy (Appendix B)—can be used for pre- and/or postsession assessment and can be provided through an online survey tool, via paper, or embedded in the PPT presentation using an ARS program.
- Multiple-choice knowledge assessment tool (Appendix C)—includes eight multiple-choice knowledge questions, one perception question, and one demographics question in a one-page format that can be administered at the end of the session on paper or provided online through a survey tool.
- Multiple-choice knowledge assessment tool with answers and references (Appendix D)—includes eight multiple-choice knowledge questions, one perception question,

Copyright © 2019 Lester et al. This is an open-access publication distributed under the terms of the Creative Commons Attribution license.

and one demographics question. This document features answers to the eight multiple-choice questions and references.

Statistical Method

Participants took the knowledge-based tests prior to exposure to the medication reconciliation educational program and after the program. Pre/post groups of participants were not paired for logistical reasons. We collected pretest scores up to 1 week prior to the educational presentations, and owing to scheduling and rotation changes, we could not pair the participants because we could not guarantee attendance and participation in the assessment tools, as rotations and call responsibilities varied often. The two groups were assumed to be independent. Seventy-one participants took the preeducational test, and 37 took the test after attending the educational program. Some of the participants may have been present in both groups. Test results were coded as a binary variable (correct or incorrect), summarized using frequencies and percentages, and compared between pre/post groups using Fisher's exact test. In addition, an aggregated score was computed for each participant by adding the scores from each question (questions 1-8) and compared between groups using the Wilcoxon rank sum test. Embedded self-efficacy assessment questions were summarized using count and percentages.

SAS 9.4 (SAS Institute, Cary, North Carolina) was used to conduct all analyses, and a result was considered statistically significant if $p < .05$.

Results

The largest group implementation using this educational module was internal medicine grand rounds and surgery grand rounds lecture presentations for faculty and house staff in our hospital. For our data analysis, we called responses *preintervention* for precourse assessments and *postintervention* for postcourse assessments. American Board of Internal Medicine faculty members were eligible for Maintenance of Certification credits and needed to complete the survey online to earn credits. There were approximately 150 attendees at all of the grand rounds during which the presentation was given. We had 74 providers completing the precourse knowledge survey and 39 completing posttests. The posttest was distributed only to those who participated in the educational program and therefore represents only providers who attended the program. We had 49 responses for the ARS embedded questions, which were collected in real time during the presentations.

Learning outcome measures with a postsession self-efficacy and knowledge assessment quiz revealed acquisition of knowledge and high perception by participants after the session that the program would improve their future medication reconciliation efforts. This demonstrated positive impact on both reactions and learning using Kirkpatrick's pyramid of responses.

Forty-nine learners answered the self-assessment using the ARS about confidence. Table 1 presents the results.

There were 37 providers who participated in the presession multiple-choice knowledge assessment. They represented various fields: PA student (one), resident (nine), fellow (two), PA/NP (11), attending physician (two), and nursing (three). There were 72 attendees who completed the postsession knowledge quiz. Attendees came from various fields: resident (one), fellow (one), PA/NP (21), attending physician (33), and nursing (14). The attendees participating in the pre- and postsession tests were not matched for logistical reasons, including scheduling conflicts. For every knowledge question, the percentage of respondents with correct answers improved from before the session (preintervention) to after the educational program (postintervention). Two of the questions had improvement that reached statistical significance. Table 2 presents the results,

Table 1. Embedded Self-Efficacy Assessment

Question	No. of Respondents	Not at All Confident	Somewhat Confident	Neutral	Confident	Extremely Confident
Asked at the beginning of a presentation:						
To what extent are you confident in your ability to do an appropriate medication reconciliation on admission TO the hospital?	49	12.2%	18.4%	10.2%	44.9%	14.3%
To what extent are you confident in your ability to do an appropriate medication reconciliation on discharge FROM hospital TO home or rehabilitation?	46	15.2%	13.0%	17.4%	30.4%	23.9%
		**Extremely Unlikely**	**Unlikely**	**Neutral**	**Likely**	**Extremely Likely**
Asked at the end of the presentation:						
Rate how likely this presentation will improve your future medication reconciliation efforts.	34	5.9%	0.0%	11.8%	41.2%	41.2%

Copyright © 2019 Lester et al. This is an open-access publication distributed under the terms of the Creative Commons Attribution license.

Table 2. Knowledge Assessment by Comparing Participants Who Participated in the Medication Reconciliation Education Program With Those Who Did Not

	No. (%) of Participants With Correct Answer		
Question	Preintervention (N = 71)	Postintervention (N = 37)	p
1: Which of the following can be used as a source for medication history?	67 (94.4)	37 (100.0)	.3
2: Which techniques should NOT be used to get the "best possible medication history"?	24 (34.3)	30 (81.1)	<.0001[a]
3: Which of the following should be included in an accurate medication list for a particular patient?	48 (67.7)	29 (78.4)	.3
4: Of all medication errors that occur during transitions of care, medication reconciliation errors account for what percentage?	29 (41.4)	25 (67.6)	.01[a]
5: Barriers to good medication reconciliation include all of the following EXCEPT:	59 (84.3)	34 (91.9)	.4
6: Which of the following is NOT a high risk for medication errors?	61 (87.1)	35 (94.6)	.3
7: Which of the following is correct about medication discrepancies?	29 (42.0)	22 (59.5)	.1
8: Which of the following is not included in the Joint Commission's process for discharge medications?	50 (69.4)	31 (83.8)	.2

[a]Denotes statistical significance with $p < .05$ using Fisher's exact test.

and Appendix C provides complete wordage of questions and answers. Scores of questions 1-8 were aggregated and compared between groups. Overall, the median (interquartile range) score of postintervention group 6 (6-7) significantly improved compared to that of preintervention group 5 (4-6), $p < .0001$ (Figure).

We did not evaluate results of internal medicine clinicians alone or surgeons alone or compare the two groups, because the internal medicine group was much larger and because we did not identify the survey responses based on field. We performed subgroup analysis based on the types of learners (combining residents and fellows into one subgroup) to evaluate for significant improvements in knowledge assessment. The only statistically significant finding was that the PA/NPs demonstrated significant improvement in question 8 about Joint Commission recommendations: from 61.9% correct in the preintervention group to 100% correct in the postintervention group, with a p value of .03.

As part of the longer questionnaire conducted before and after the educational model, we asked participants their perception of how important accurate medication reconciliation was in caring for hospitalized patients. Both prior to the presentation and after the presentation, 97% of respondents said that it was extremely important.

Analysis of a post hoc focus group provided very positive feedback. Learners reported appreciation of the comprehensive approach delineated in our presentation. They also appreciated the real-life examples that allowed them to better recognize the potentially detrimental impact of inappropriate medication reconciliation. The learners expressed feeling more confident about how to obtain the best possible medication history and how to utilize hospital resources to assist them.

Discussion

We aimed to improve knowledge and self-efficacy about medication reconciliation for internal medicine and surgery clinicians, including trainees, mid-level providers, and attending physicians. We were successful in reaching a range of clinician levels with our educational program using PPT slides with self-assessment and knowledge assessment tools. Based on verbal feedback about the presentations, both internal medicine and surgery were receptive to the information provided.

Our educational program was successful in improving learners' knowledge in every question we tested; however, only two of the improvements were statistically significant. Perhaps with a higher number of participants, especially if pre- and postintervention groups were of similar size, there would be sufficient power to show statistically significant improvements. Although only one question was answered correctly by 100% of respondents after the educational module, it is reassuring that improvement occurred in every question asked. The two questions that reached statistically significant improvement in percentage with correct answers (questions 2 and 4) also had the lowest percentage of correct answers in the preintervention assessment. This seems logical considering that a lower starting point provided more room for improvement after the educational program. However, question 7 about medication discrepancies had a similarly low starting point (only 42.0% correct), but its improvement postintervention did not reach statistical significance. This suggests that efforts to focus on the items covered in question 7 may require more emphasis.

Additionally, it is notable that both before and after the presentation, there was virtually universal recognition of the importance of medication reconciliation for hospitalized patients, as this demonstrates a widespread awareness of its important

Copyright © 2019 Lester et al. This is an open-access publication distributed under the terms of the Creative Commons Attribution license.

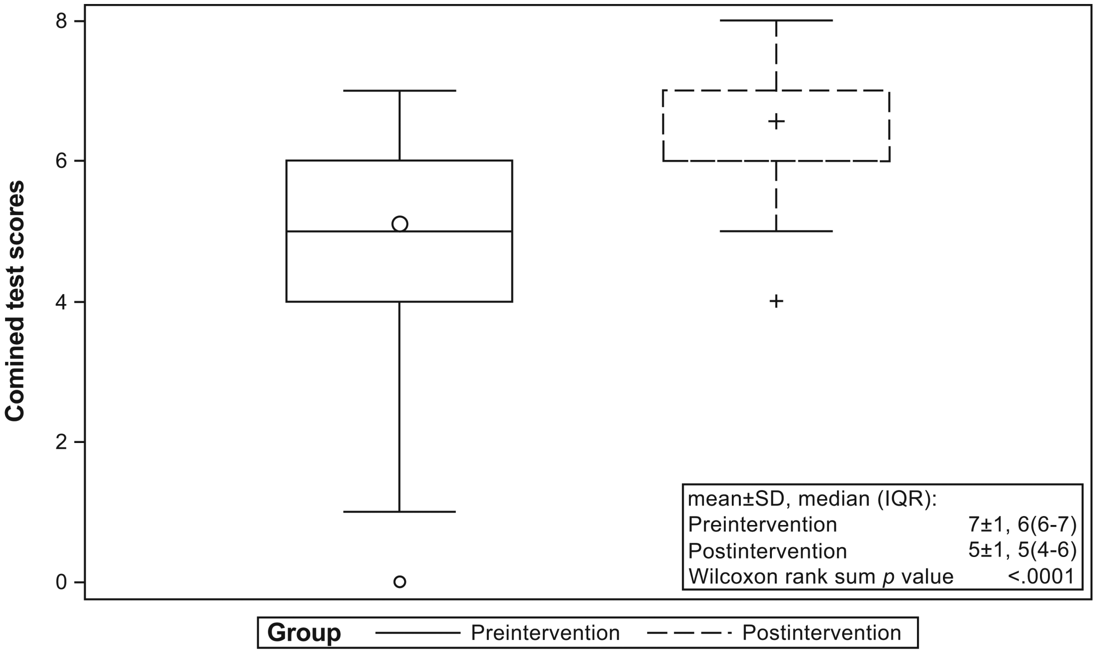

Figure. Combined knowledge-based test scores. Box plots show 25th, 50th (median), and 75th percentiles (horizontal bars). The lower fence is 1.5 × interquartile range (IQR) below the 25th percentile. The upper fence is 1.5 × IQR above the 75th percentile. The circle and plus sign inside the boxes are means. The circle and plus sign outside the fences are outliers.

role in high-quality patient care. Nonetheless, most attendees (82.4%) reported that the presentation was likely or extremely likely to improve their future medication reconciliation efforts. This reveals that although the respondents previously knew medication reconciliation was important, they still found the educational module informative and valuable.

The preintervention data showed that 30.6% of attendees were not at all confident or only somewhat confident in conducting an appropriate medication reconciliation on admission to the hospital. This is concerning because mistakes on the initial hospital medication reconciliation list can reverberate throughout a medical stay and have significant harmful impact on patient care. Almost half (45.7%) of attendees in the preintervention assessment reported that they were not confident or extremely confident in performing appropriate discharge from the hospital to home or a rehabilitation facility. This too is disturbing, as the discharge medication plan from hospitalization is vital to successful implementation of the treatment plans developed during acute hospitalization.

There are several limitations of our research. First, we did not match each individual for testing results in comparing individual knowledge gained by attending the session. Although doing so would have made results more powerful and likely more significant, the logistics of matching were not feasible in our institution because of on-call responsibilities and rotation changes. Arguably, anonymous completion of knowledge tools is more likely to be honest, as there is less concern for being judged or criticized for erroneous answers. Additionally, more participants answered the preintervention knowledge tool than completed the postintervention knowledge tool after the presentation. Unfortunately, busy schedules often make it difficult for attendees to complete a posttest immediately after a course, and therefore, the precourse test was distributed via email 1 week prior to the presentation, allowing more schedule flexibility to participate. As with any educational program, there is a challenge to train new house staff and employees in response to turnover. Online or enduring educational modules can reduce the burden of providing repeated presentations. For the purpose of future research, it might be beneficial to attempt to use identifiers for pre- and postintervention with the caveat that they may not all be matched due to scheduling conflicts.

This educational program about medication reconciliation provides useful training for clinicians to use when admitting or discharging hospital patients. Individual institutions can add any specific policies implemented in their facilities and include real-life instances of medication errors and examples of forms used for medication reconciliation. Future research could be designed

Copyright © 2019 Lester et al. This is an open-access publication distributed under the terms of the Creative Commons Attribution license.

to assess impact on behavior and results as an outcome of this educational module. A well-rehearsed presentation is imperative to effectively present these educational materials. An experienced session facilitator may choose to edit or redesign content for local needs and institutional policies. For example, some facilities have defined medication reconciliation templates in their EHRs. Learners reported that the educational module was effective and useful and requested copies of the PPT so they could refer to it as a reference source in the future.

Appendices

A. Medication Reconciliation Slides.pptx

B. Embedded ARS Questions.docx

C. Pre-Post Assessment.docx

D. Pre-Post Assessment Answers and References.docx

All appendices are peer reviewed as integral parts of the Original Publication.

Paula E. Lester, MD: Associate Fellowship Program Director, Geriatric Medicine, NYU Winthrop Hospital; Associate Professor of Medicine, NYU Long Island School of Medicine

Sukhminder Sahansra, MD: Clinical Assistant Professor of Medicine, NYU Long Island School of Medicine

Mark Shen, PharmD: Clinical Pharmacist, NYU Winthrop Hospital

Maria Becker, DO: Family Practice Resident, Peconic Bay Medical Center

Shahidul Islam, DrPH: Research Assistant Professor, Division of Health Services Research, NYU Long Island School of Medicine

Acknowledgments
The authors acknowledge Andrew Mak, MD, MBA, and Lore Von Hoffen, PA-C, for copresenting this educational module.

Disclosures
None to report.

Funding/Support
None to report.

Ethical Approval
Reported as not applicable.

References

1. Medication reconciliation to prevent adverse drug events. Institute for Healthcare Improvement website. http://www.ihi.org/Topics/ADEsMedicationReconciliation/Pages/default.aspx. Accessed July 3, 2018.
2. Hennen CR, Jorgensen JA. Importance of medication reconciliation in the continuum of care. *Am J Pharm Benefits*. 2014;6(2):71-75.
3. Midelfort L. Medication reconciliation review. Institute for Healthcare Improvement website. http://www.ihi.org/resources/Pages/Tools/MedicationReconciliationReview.aspx. Accessed July 3, 2018.
4. Cornish PL, Knowles SR, Marchesano R, et al. Unintended medication discrepancies at the time of hospital admission. *Arch Intern Med*. 2005;165(4):424-429. https://doi.org/10.1001/archinte.165.4.424
5. Silvestre CC, Santos LMC, Silva ROS, et al. Risk factors for unintentional medication discrepancies at hospital admission: a matched case-control study. *Eur J Intern Med*. 2017;40:e24-e25. https://doi.org/10.1016/j.ejim.2017.02.003
6. McShane M, Stark R. Medication reconciliation in the hospital: an interactive case-based session for internal medicine residents. *MedEdPORTAL*. 2018;14:10770. https://doi.org/10.15766/mep_2374-8265.10770
7. Sando KR, Doty R, Elliott J, Stanton ML. Medication history lab and assessment using the Medication Mysteries Infinite Case Tool. *MedEdPORTAL*. 2016;12:10519. https://doi.org/10.15766/mep_2374-8265.10519

Received: January 15, 2019
Accepted: July 11, 2019
Published: November 1, 2019

Copyright © 2019 Lester et al. This is an open-access publication distributed under the terms of the Creative Commons Attribution license.
