# Supplementary material for: Medication Reconciliation: An Educational Module
Source: MedEdPORTAL. 2019 Nov 1;15:10852. doi: 10.15766/mep_2374-8265.10852 (PMC6952281; doi:10.15766/mep_2374-8265.10852)
Supplement: Supplementary file 1 — A. Medication Reconciliation Slides.pptx B. Embedded ARS Questions.docx C. Pre-Post Assessment.docx D. Pre-Post Assessment Answers and References.docx [file mep-15-10852-s001.zip › A. Medication Reconciliation Slides.pptx]

## Slide 1
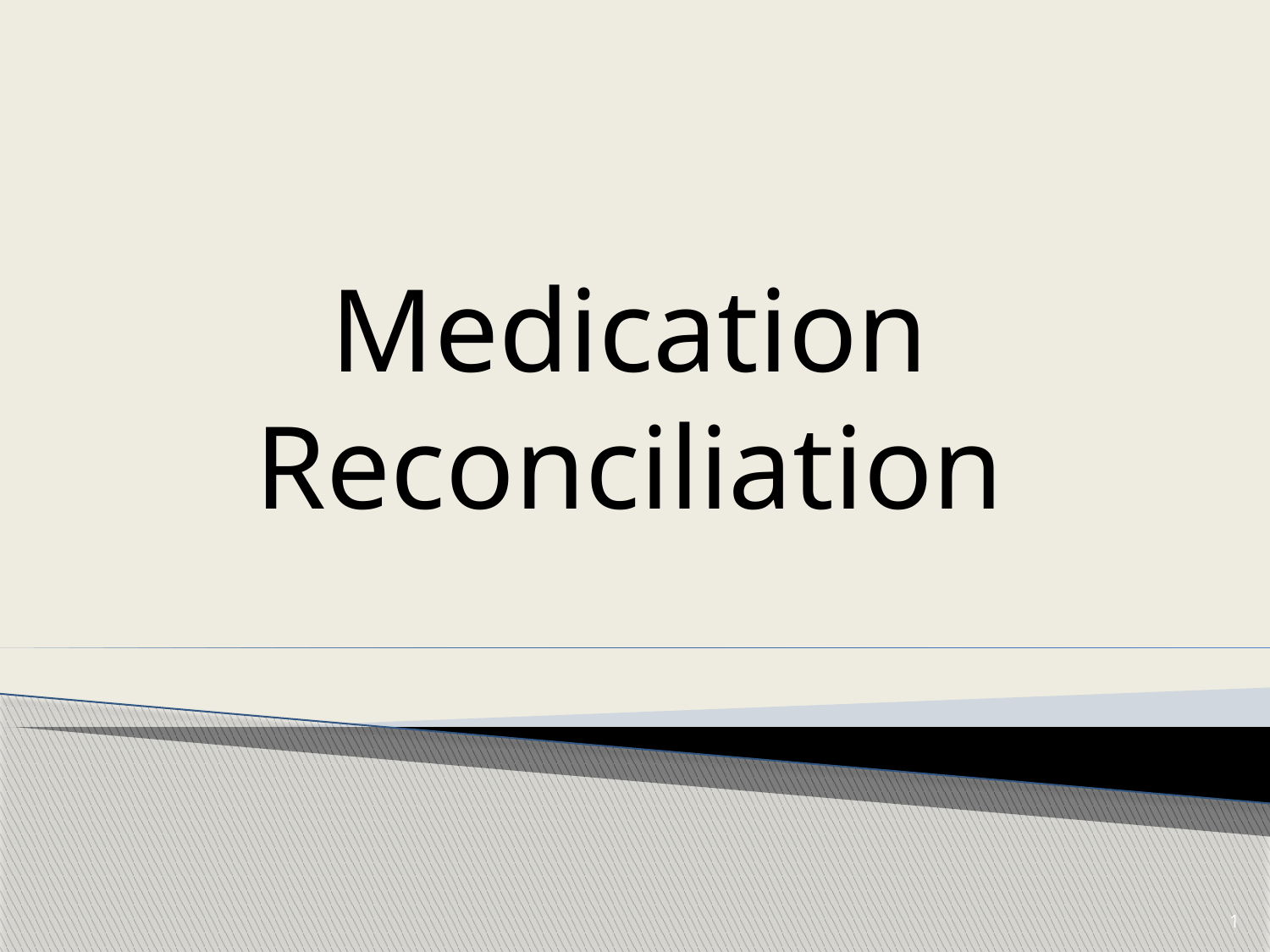

#
Medication Reconciliation
1

## Slide 2
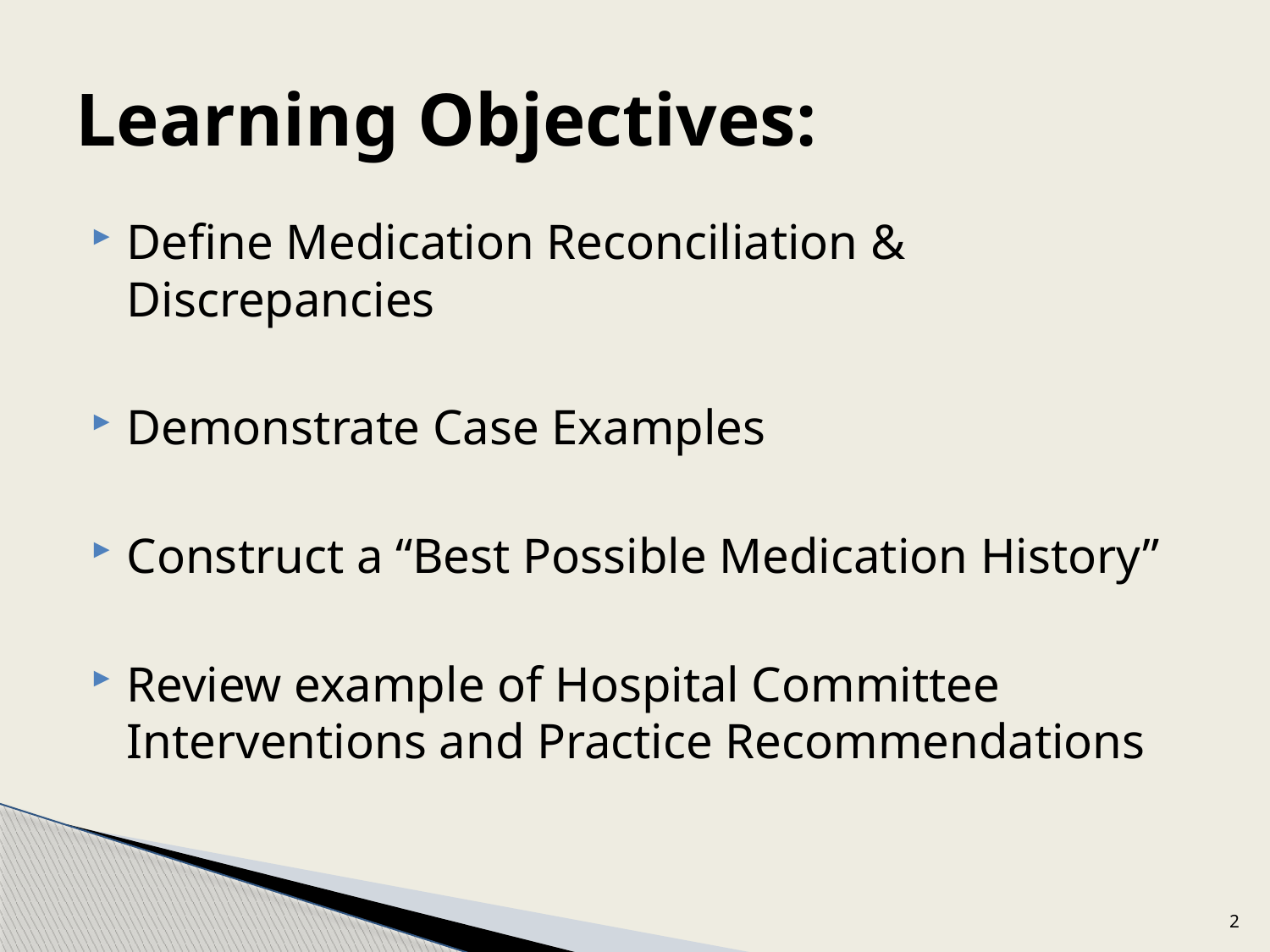

# Learning Objectives:
Define Medication Reconciliation & Discrepancies
Demonstrate Case Examples
Construct a “Best Possible Medication History”
Review example of Hospital Committee Interventions and Practice Recommendations
2

## Slide 3
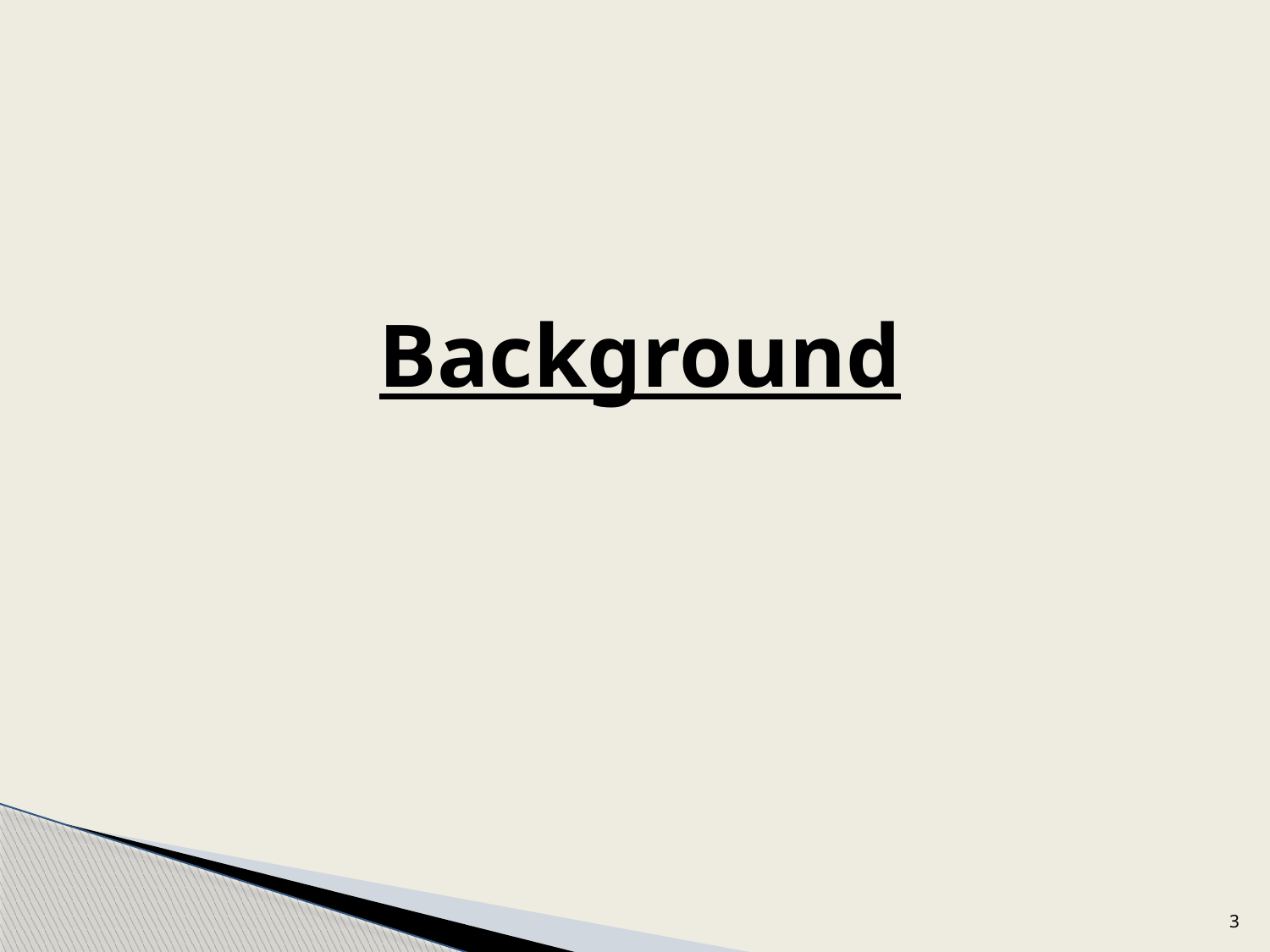

# Background
3

## Slide 4
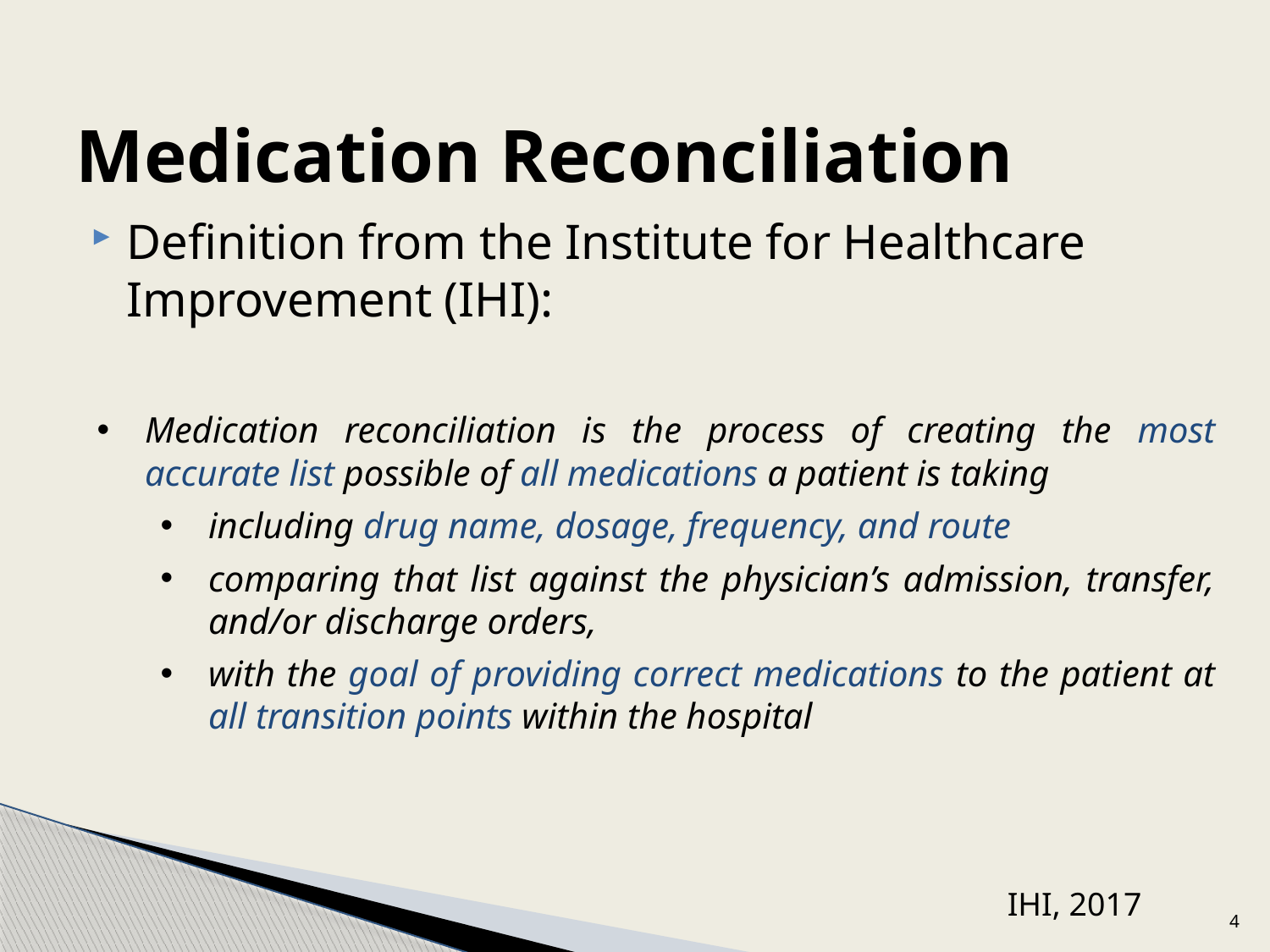

# Medication Reconciliation
Definition from the Institute for Healthcare Improvement (IHI):
Medication reconciliation is the process of creating the most accurate list possible of all medications a patient is taking
including drug name, dosage, frequency, and route
comparing that list against the physician’s admission, transfer, and/or discharge orders,
with the goal of providing correct medications to the patient at all transition points within the hospital
IHI, 2017
4

## Slide 5
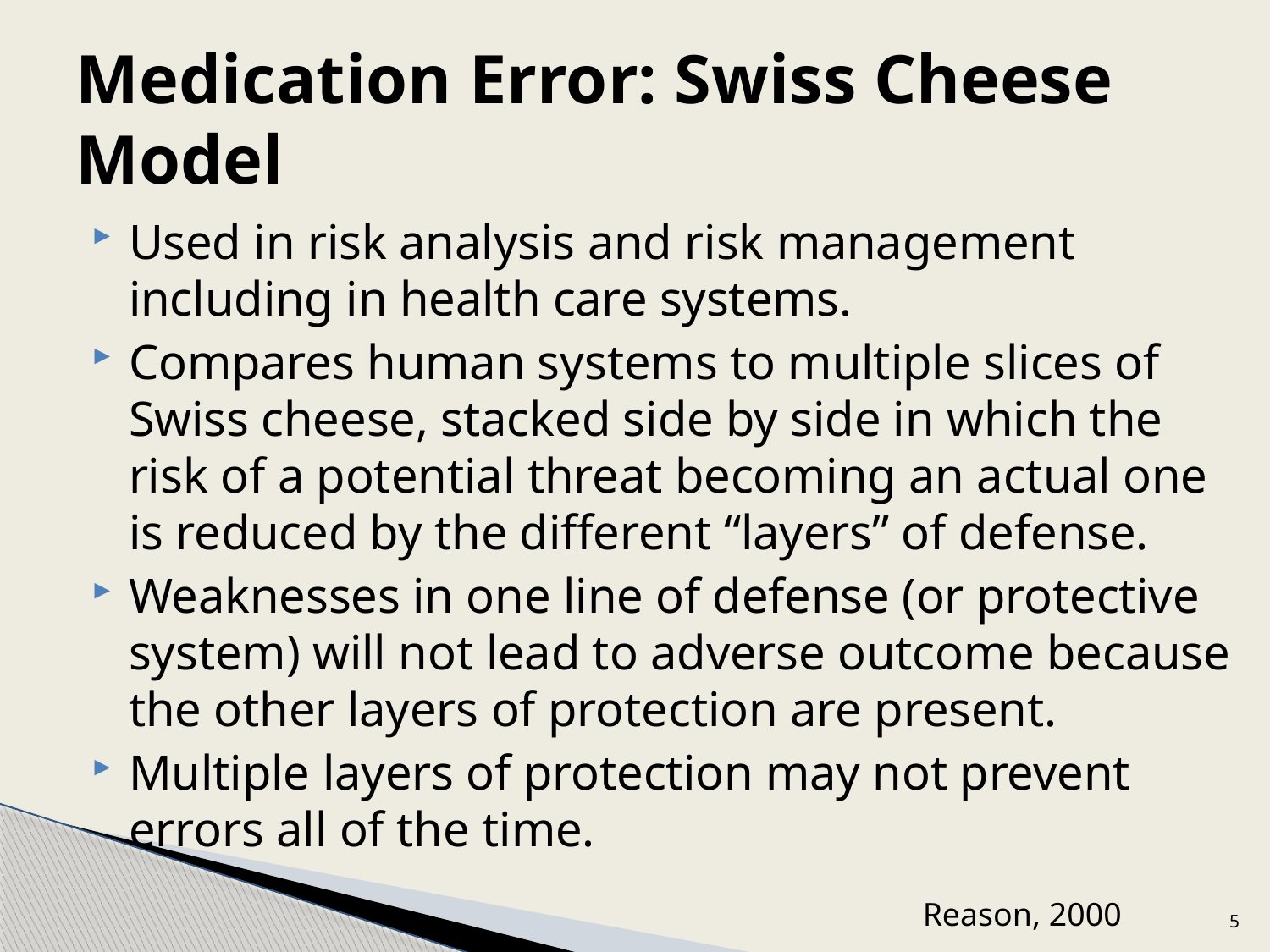

# Medication Error: Swiss Cheese Model
Used in risk analysis and risk management including in health care systems.
Compares human systems to multiple slices of Swiss cheese, stacked side by side in which the risk of a potential threat becoming an actual one is reduced by the different “layers” of defense.
Weaknesses in one line of defense (or protective system) will not lead to adverse outcome because the other layers of protection are present.
Multiple layers of protection may not prevent errors all of the time.
Reason, 2000
5

## Slide 6
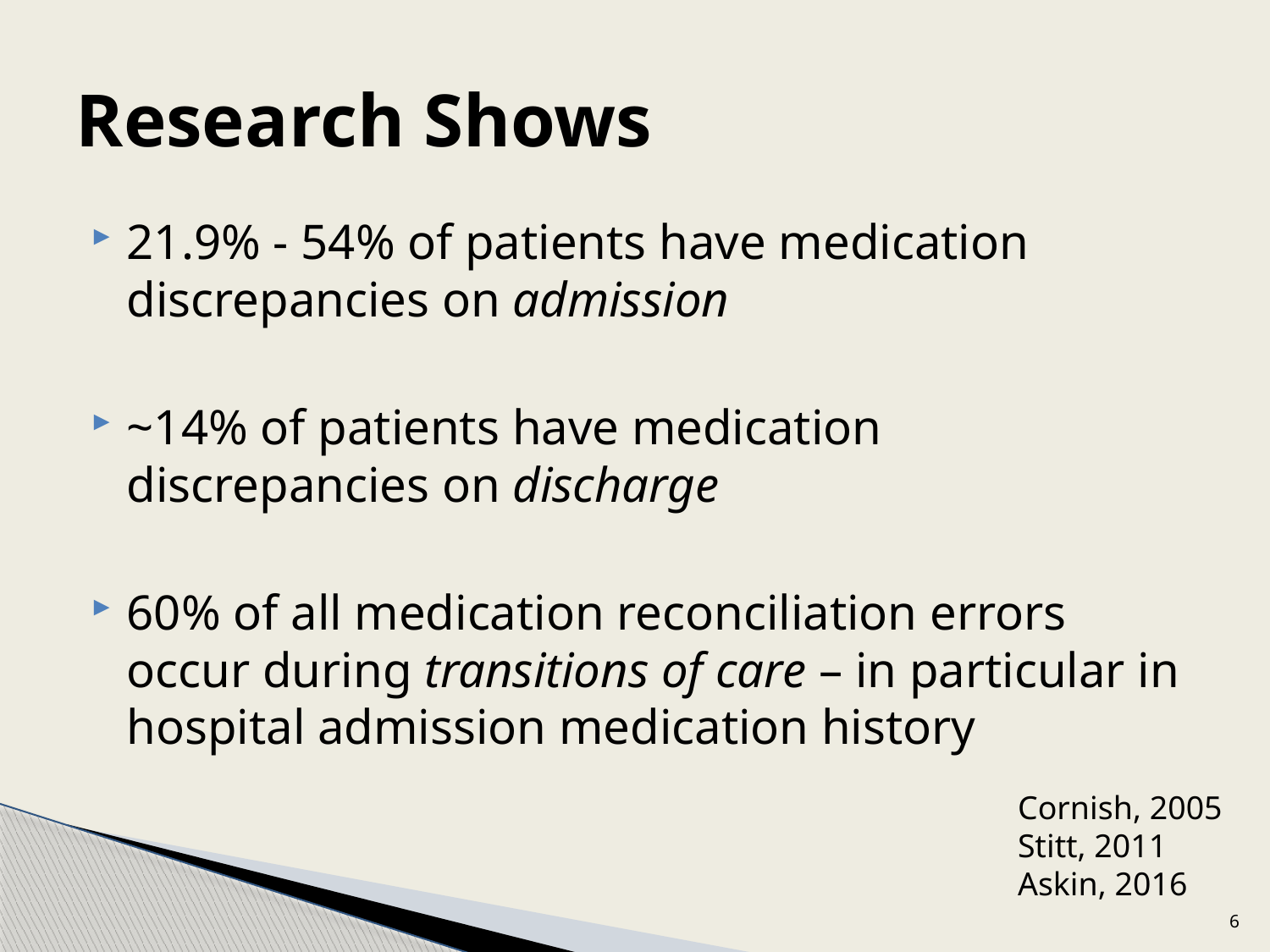

# Research Shows
21.9% - 54% of patients have medication discrepancies on admission
~14% of patients have medication discrepancies on discharge
60% of all medication reconciliation errors occur during transitions of care – in particular in hospital admission medication history
Cornish, 2005
Stitt, 2011
Askin, 2016
6

## Slide 7
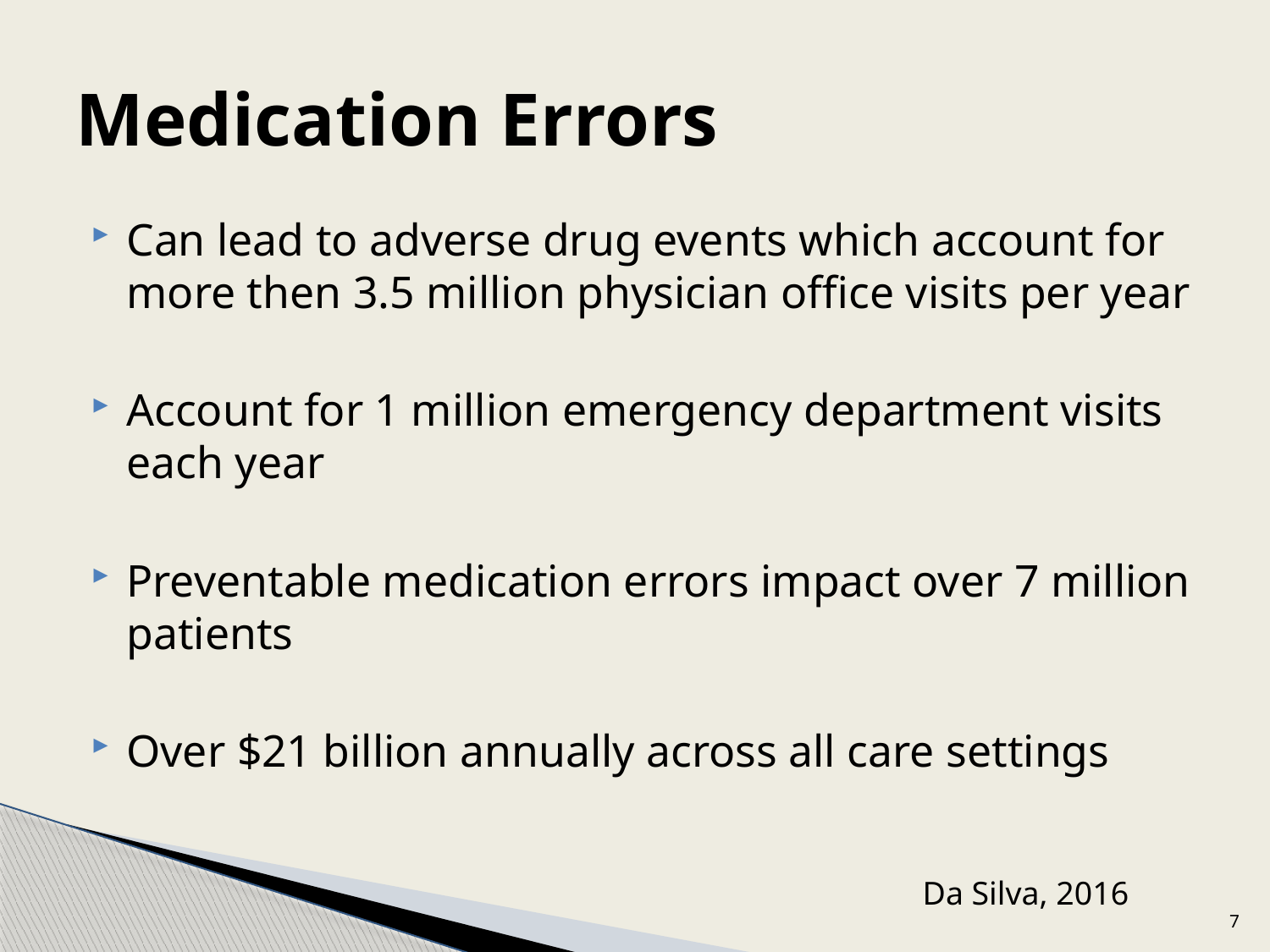

# Medication Errors
Can lead to adverse drug events which account for more then 3.5 million physician office visits per year
Account for 1 million emergency department visits each year
Preventable medication errors impact over 7 million patients
Over $21 billion annually across all care settings
Da Silva, 2016
7

## Slide 8
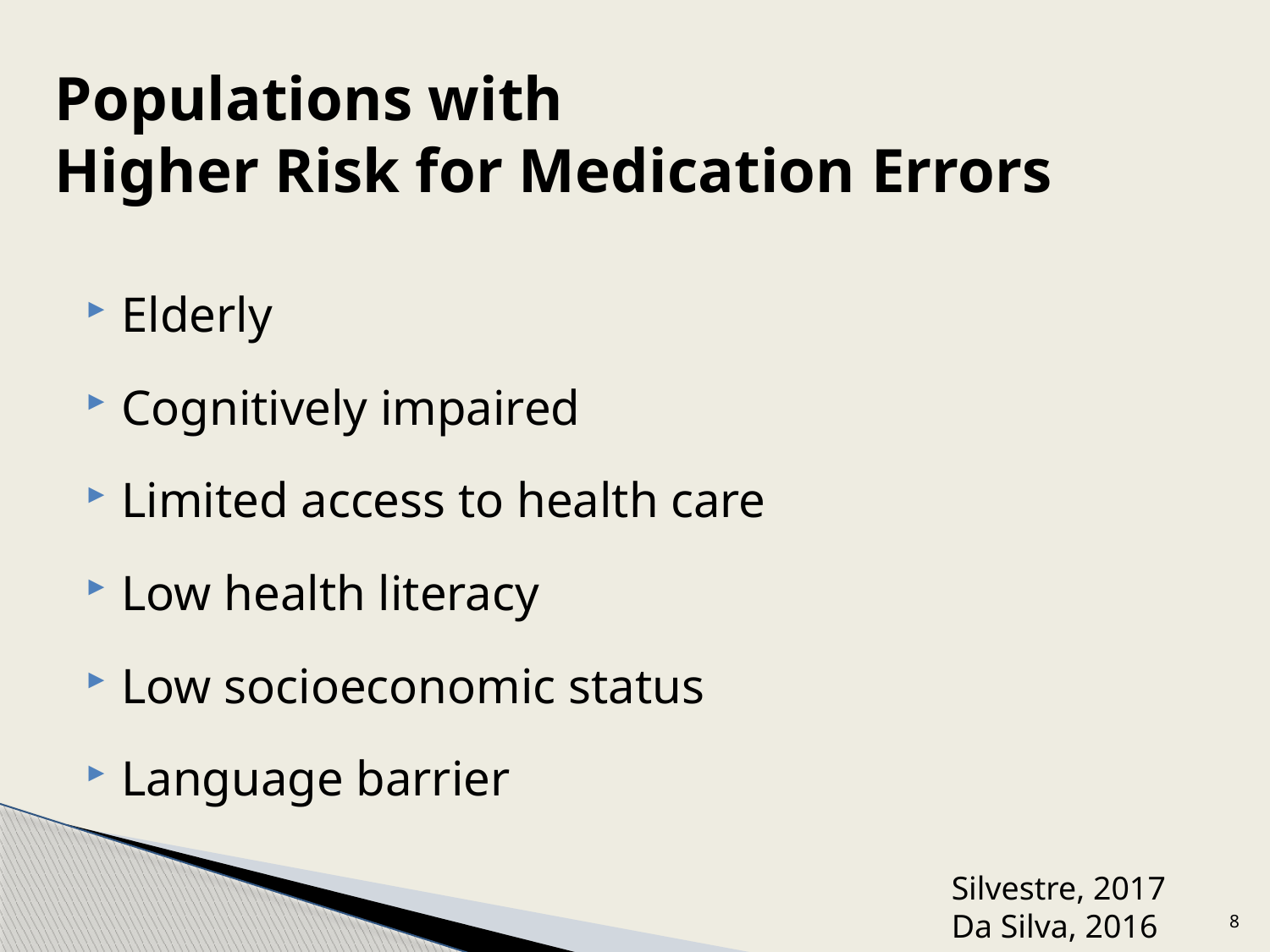

# Populations with Higher Risk for Medication Errors
Elderly
Cognitively impaired
Limited access to health care
Low health literacy
Low socioeconomic status
Language barrier
Silvestre, 2017
Da Silva, 2016
8

## Slide 9
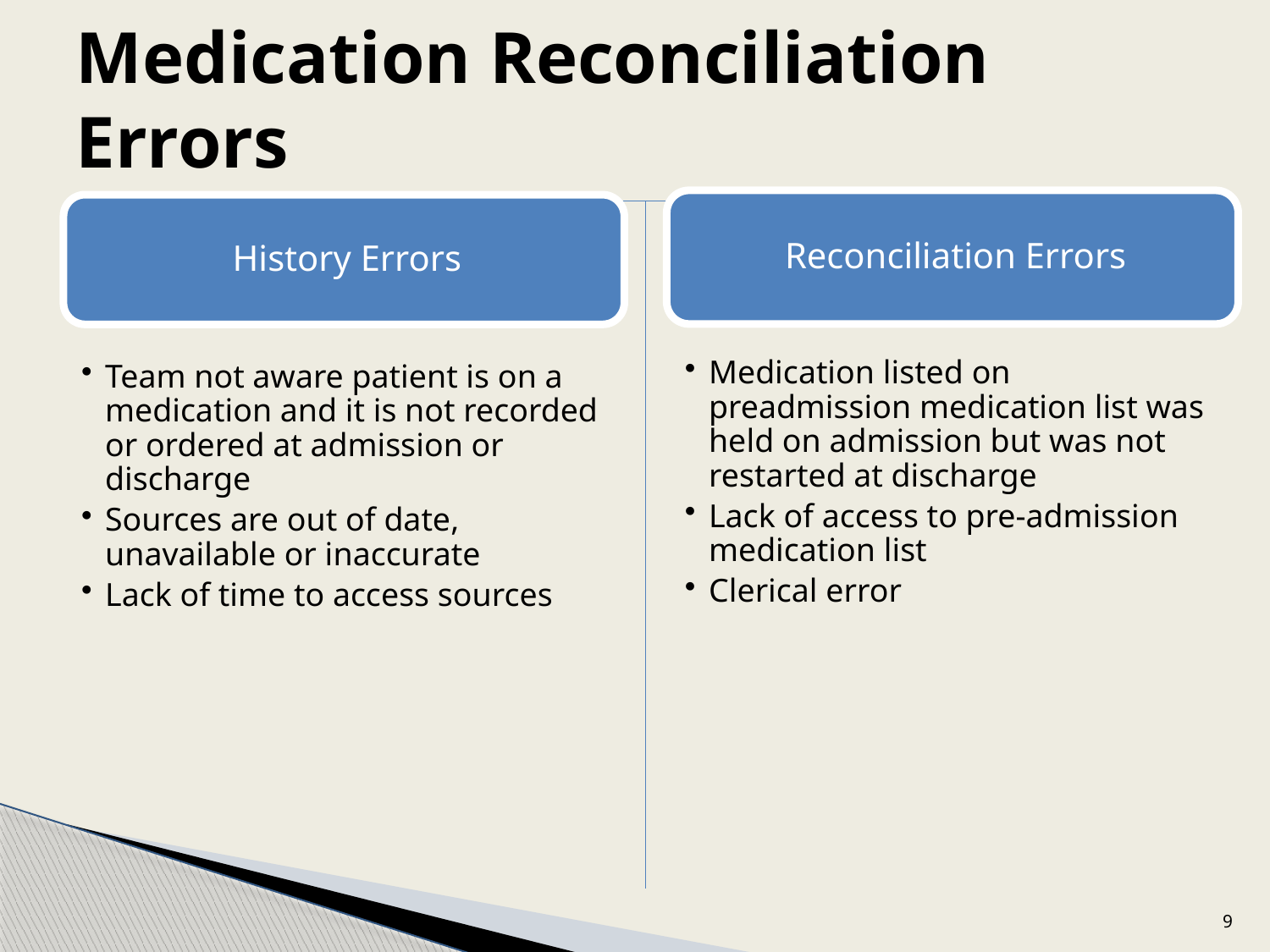

# Medication Reconciliation Errors
9

## Slide 10
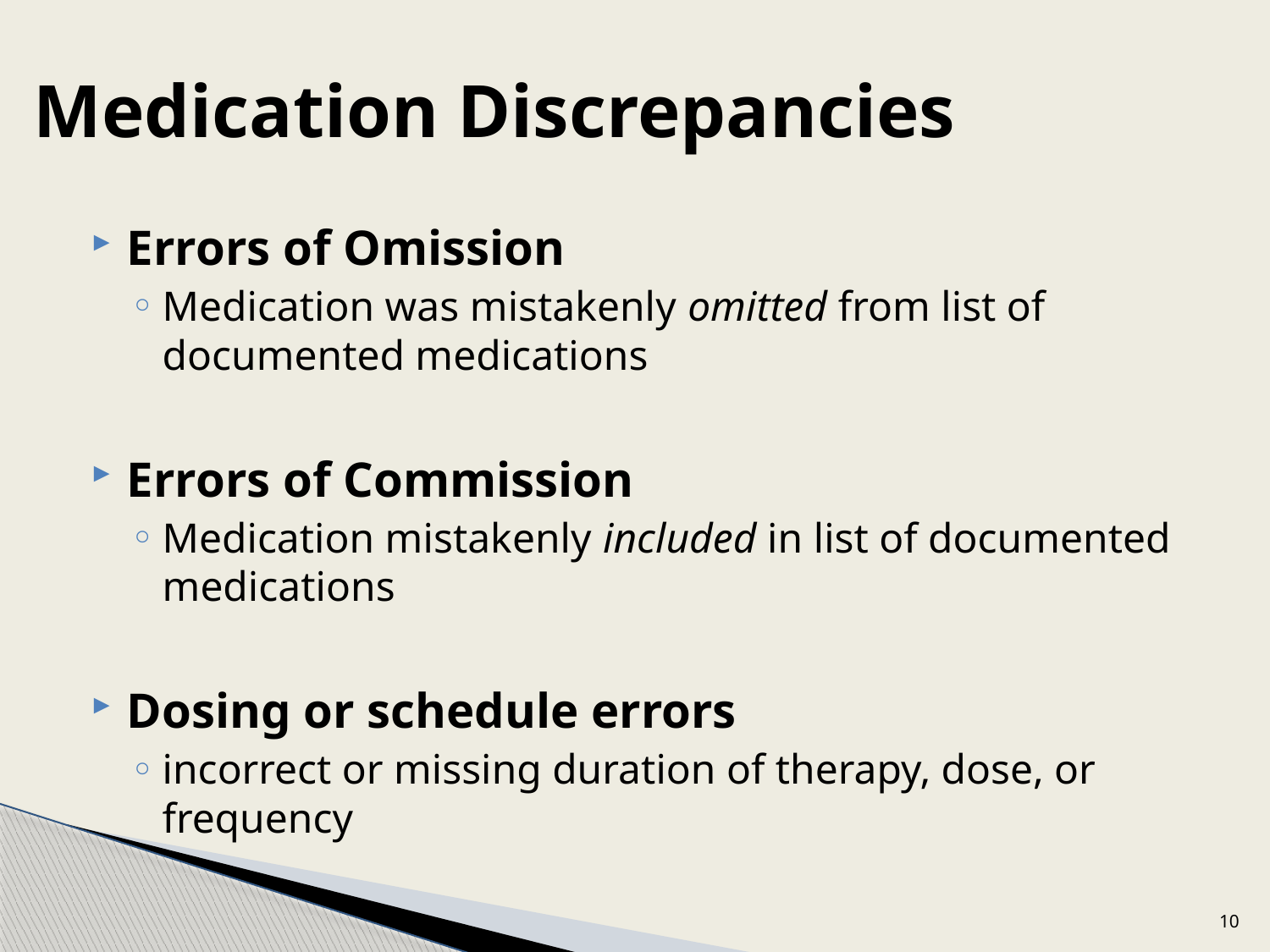

# Medication Discrepancies
Errors of Omission
Medication was mistakenly omitted from list of documented medications
Errors of Commission
Medication mistakenly included in list of documented medications
Dosing or schedule errors
incorrect or missing duration of therapy, dose, or frequency
10

## Slide 11
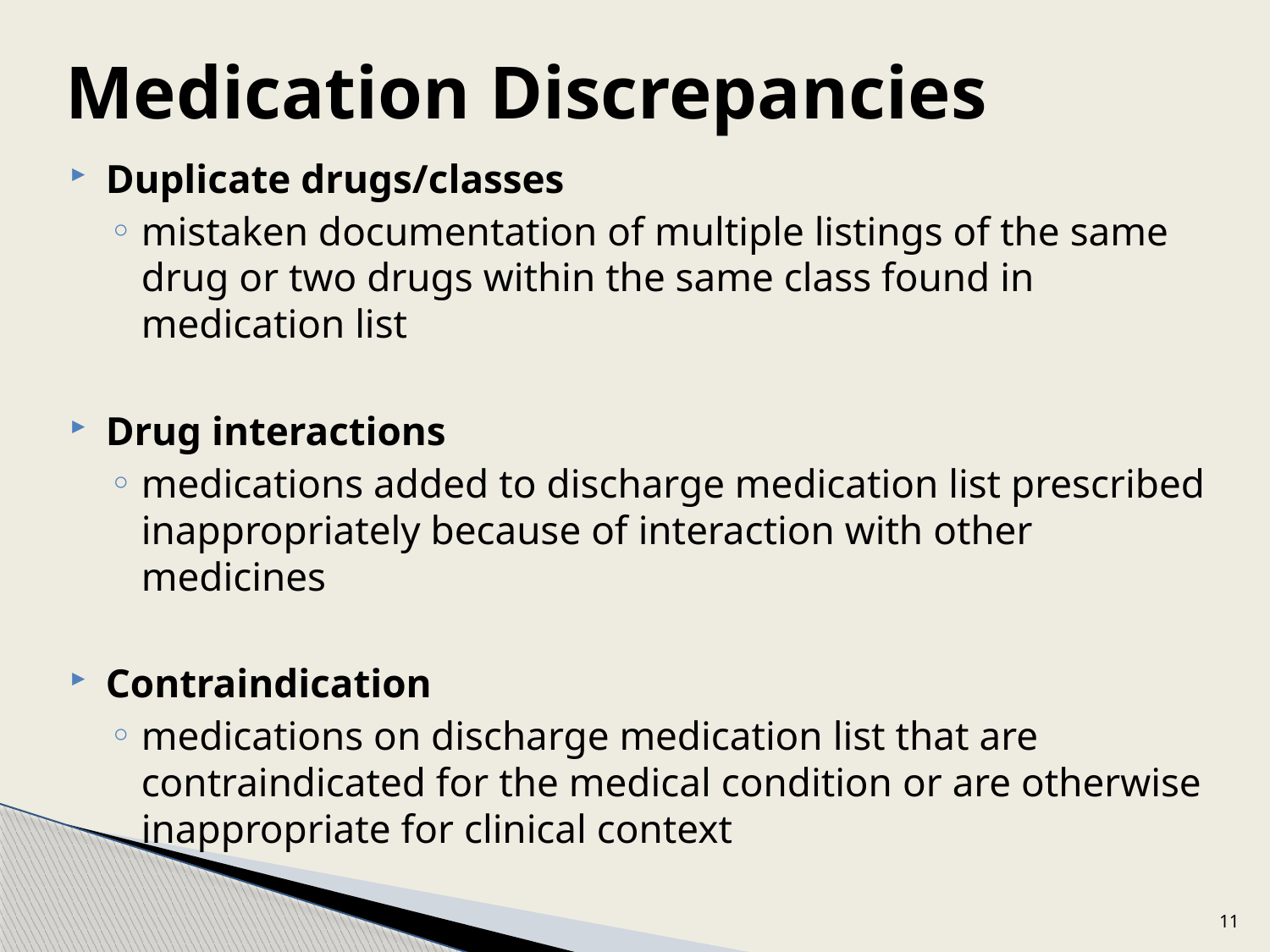

# Medication Discrepancies
Duplicate drugs/classes
mistaken documentation of multiple listings of the same drug or two drugs within the same class found in medication list
Drug interactions
medications added to discharge medication list prescribed inappropriately because of interaction with other medicines
Contraindication
medications on discharge medication list that are contraindicated for the medical condition or are otherwise inappropriate for clinical context
11

## Slide 12
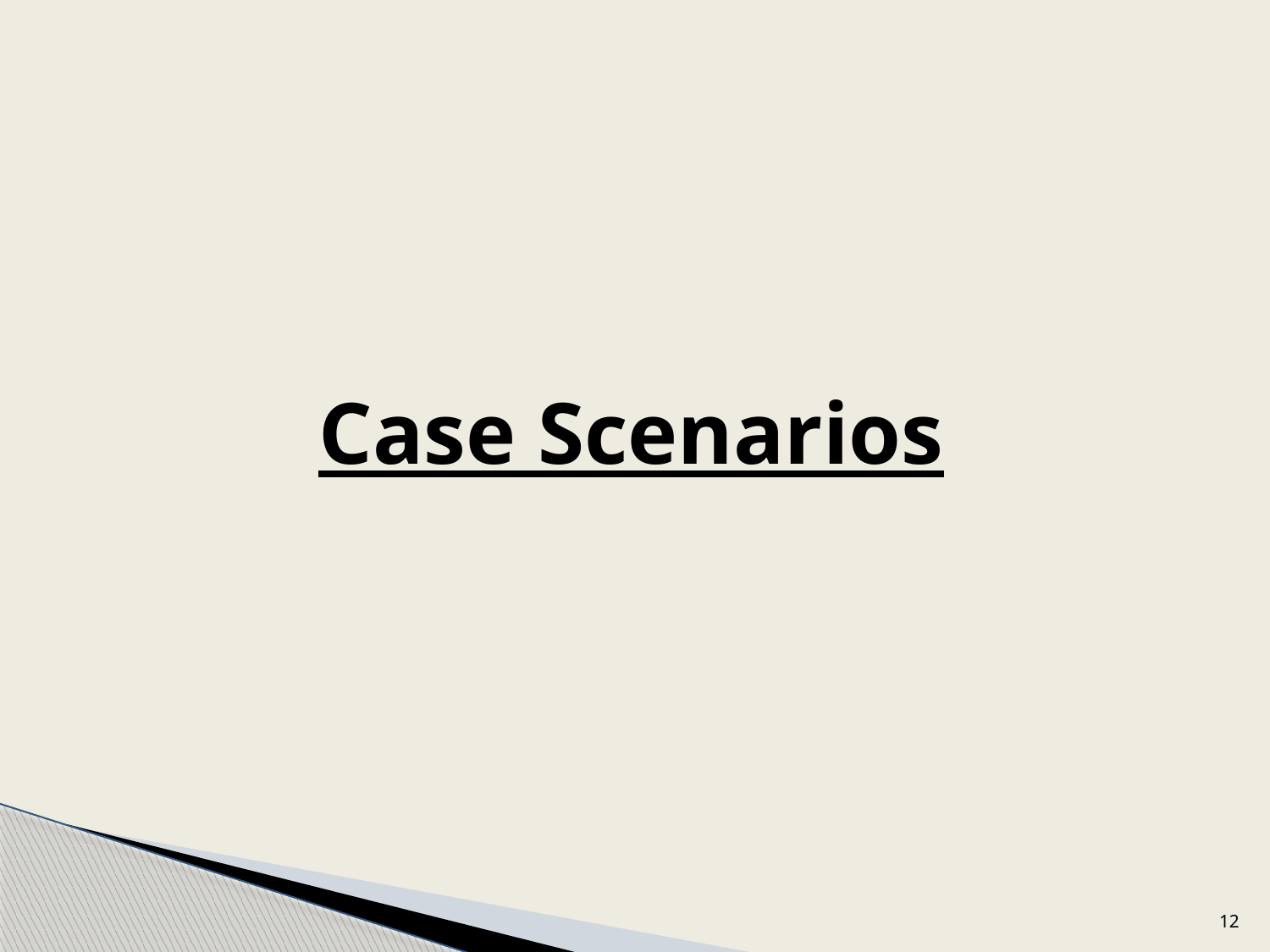

# Case Scenarios
12

## Slide 13
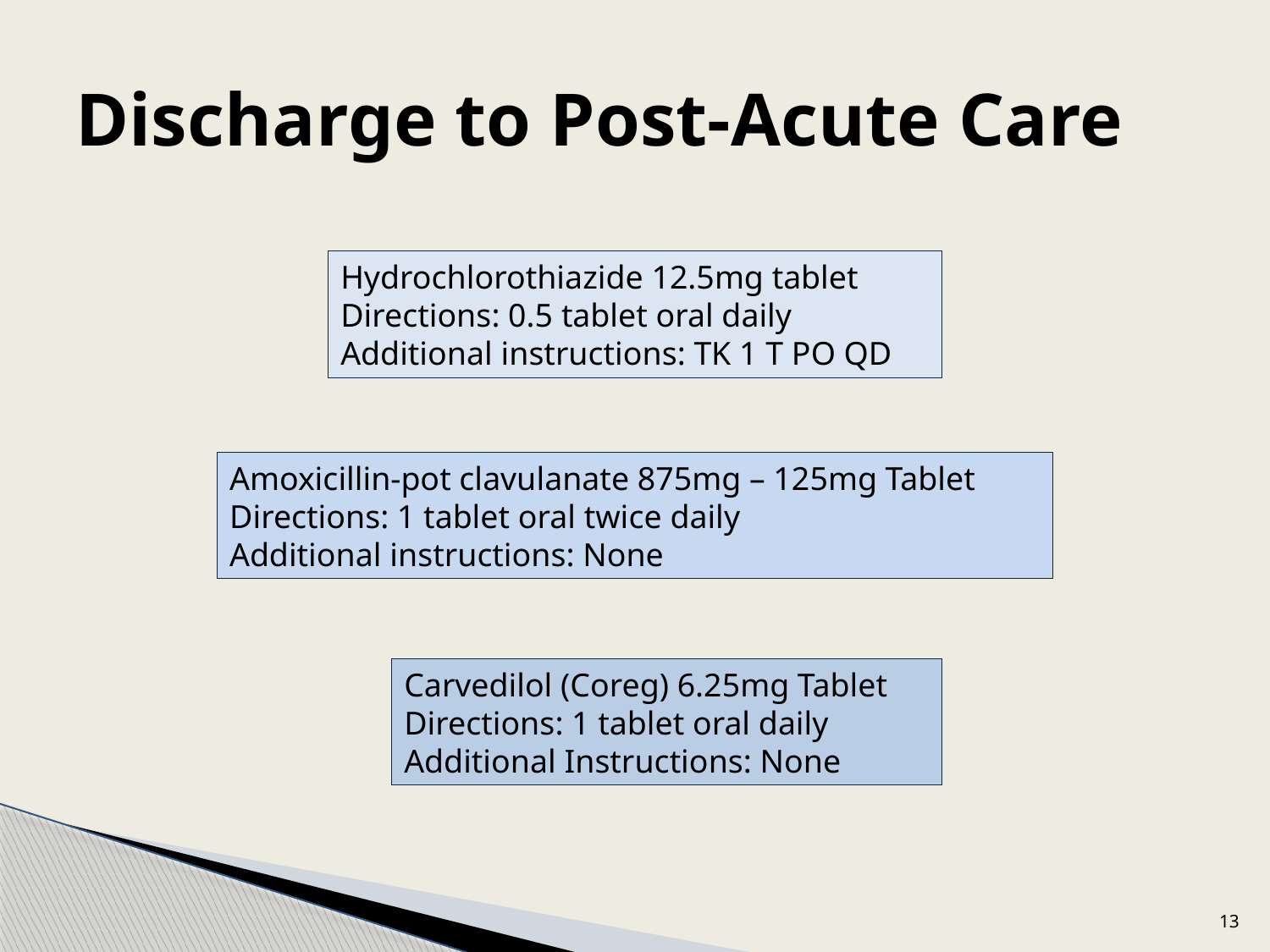

# Discharge to Post-Acute Care
Hydrochlorothiazide 12.5mg tablet
Directions: 0.5 tablet oral daily
Additional instructions: TK 1 T PO QD
Amoxicillin-pot clavulanate 875mg – 125mg Tablet
Directions: 1 tablet oral twice daily
Additional instructions: None
Carvedilol (Coreg) 6.25mg Tablet
Directions: 1 tablet oral daily
Additional Instructions: None
13

## Slide 14
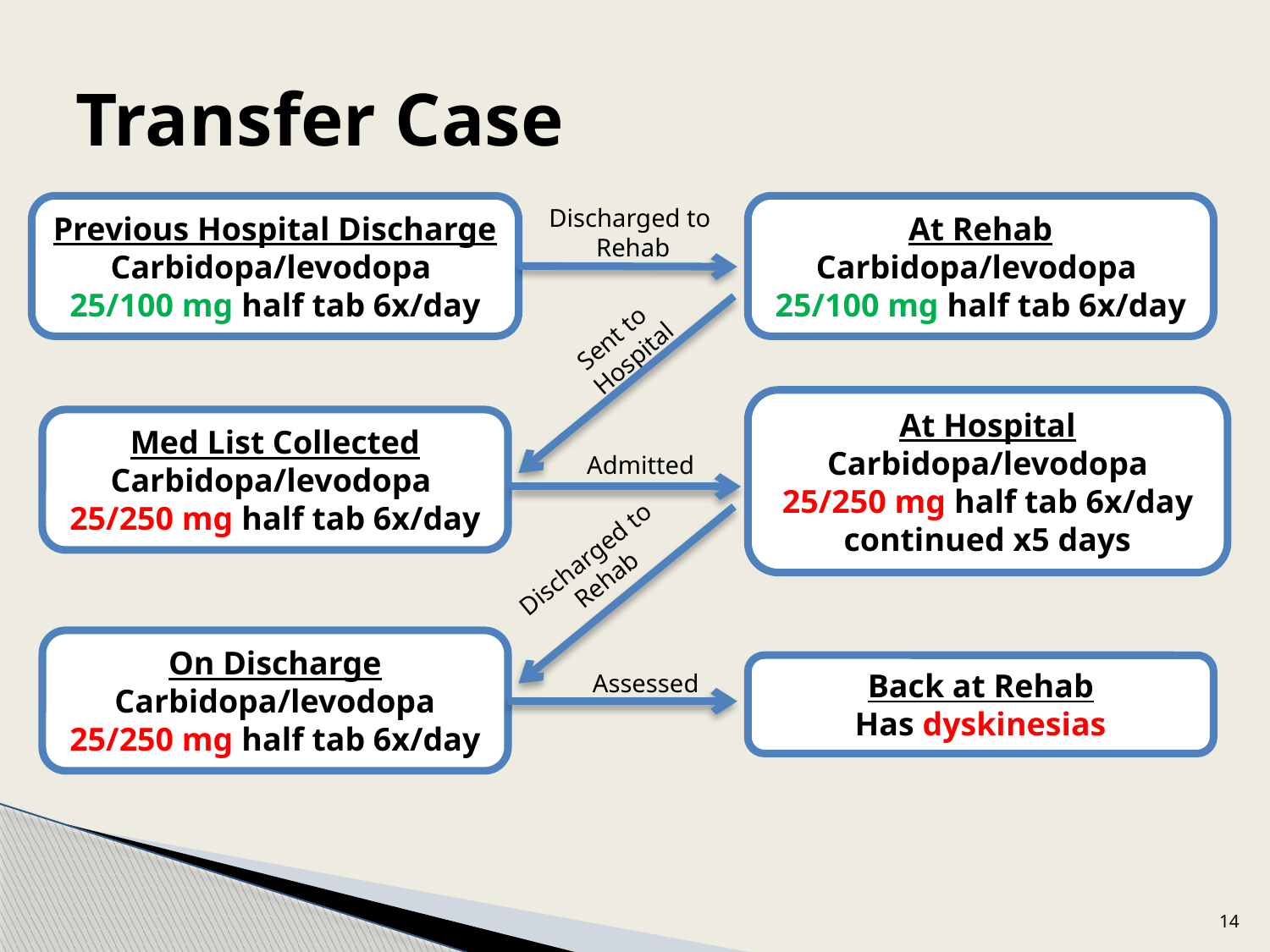

# Transfer Case
Previous Hospital Discharge
Carbidopa/levodopa
25/100 mg half tab 6x/day
At Rehab
Carbidopa/levodopa
25/100 mg half tab 6x/day
Discharged to
Rehab
Sent to
Hospital
At Hospital
Carbidopa/levodopa
25/250 mg half tab 6x/day continued x5 days
Med List Collected
Carbidopa/levodopa
25/250 mg half tab 6x/day
Admitted
Discharged to
Rehab
On Discharge
Carbidopa/levodopa
25/250 mg half tab 6x/day
Back at Rehab
Has dyskinesias
Assessed
14

## Slide 15
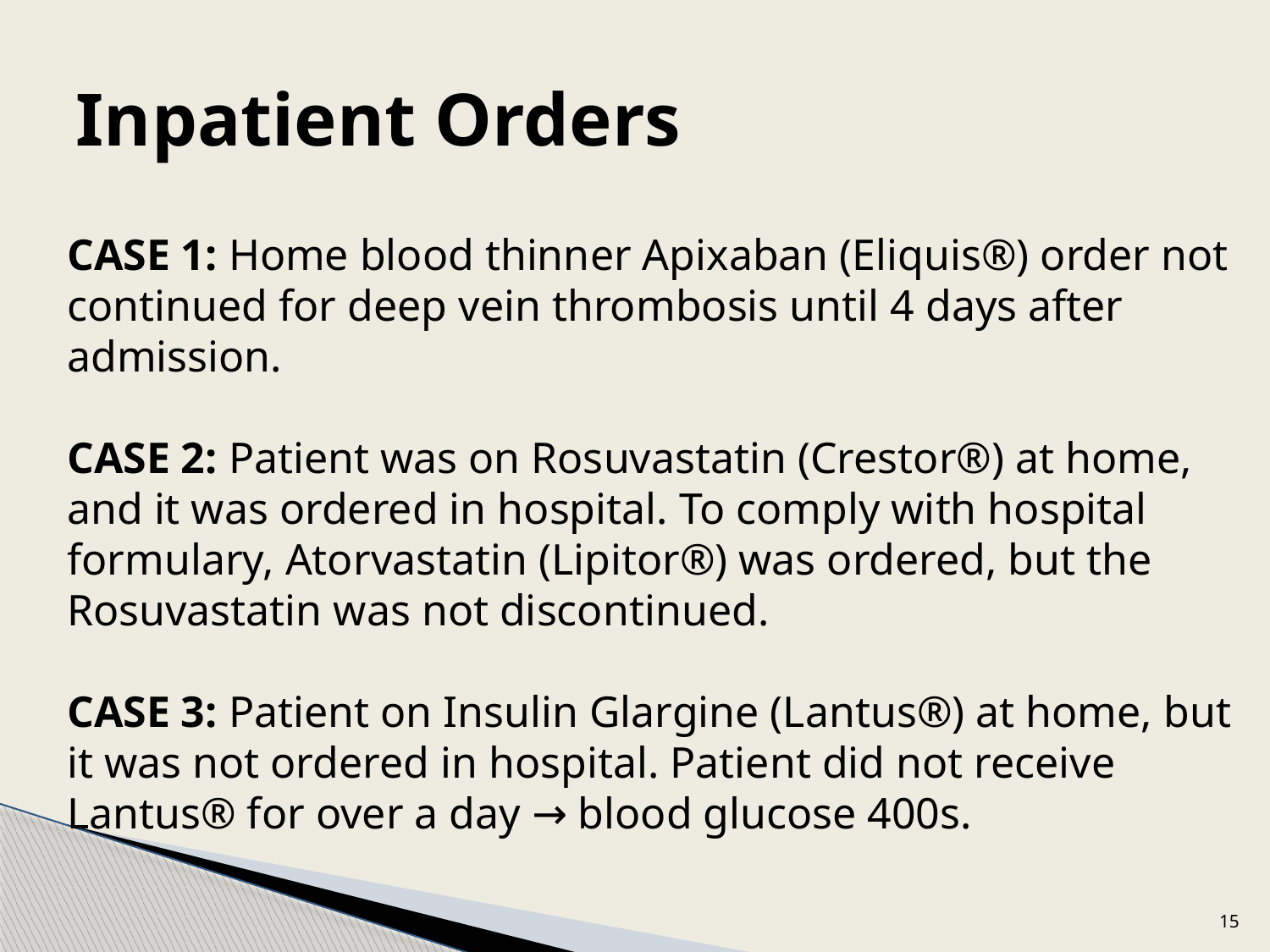

# Inpatient Orders
CASE 1: Home blood thinner Apixaban (Eliquis®) order not continued for deep vein thrombosis until 4 days after admission.
CASE 2: Patient was on Rosuvastatin (Crestor®) at home, and it was ordered in hospital. To comply with hospital formulary, Atorvastatin (Lipitor®) was ordered, but the Rosuvastatin was not discontinued.
CASE 3: Patient on Insulin Glargine (Lantus®) at home, but it was not ordered in hospital. Patient did not receive Lantus® for over a day → blood glucose 400s.
15

## Slide 16
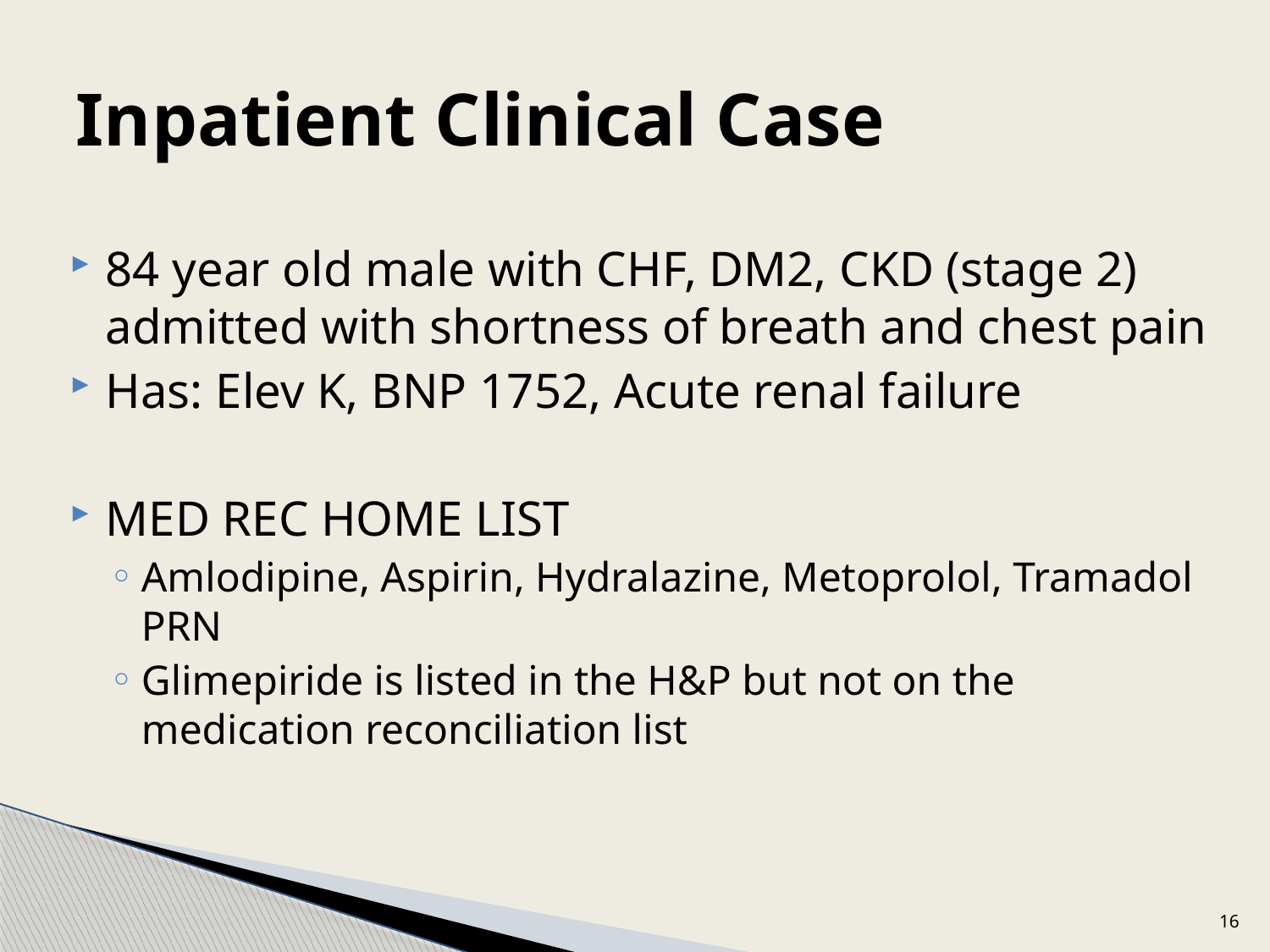

# Inpatient Clinical Case
84 year old male with CHF, DM2, CKD (stage 2) admitted with shortness of breath and chest pain
Has: Elev K, BNP 1752, Acute renal failure
MED REC HOME LIST
Amlodipine, Aspirin, Hydralazine, Metoprolol, Tramadol PRN
Glimepiride is listed in the H&P but not on the medication reconciliation list
16

## Slide 17
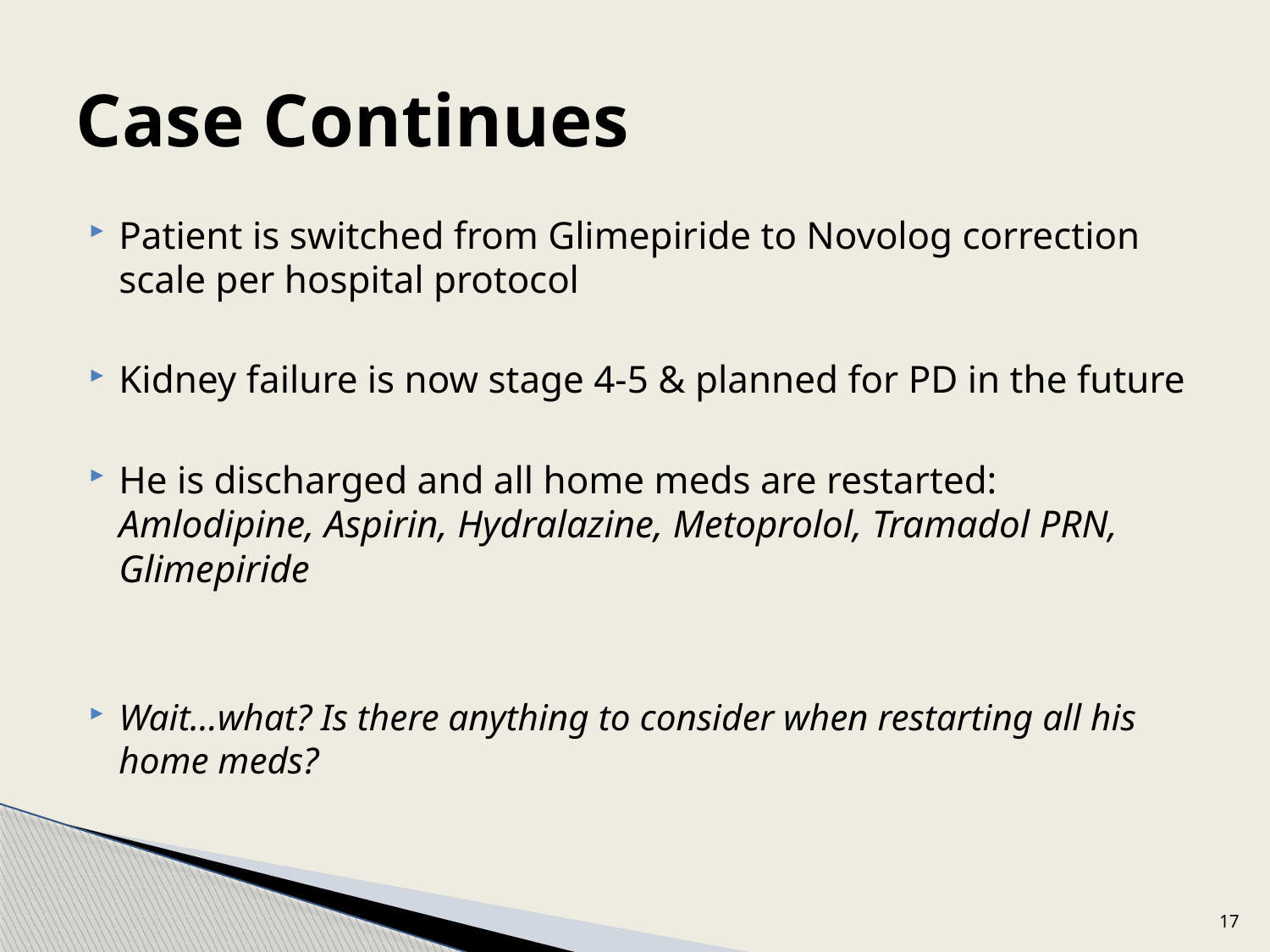

# Case Continues
Patient is switched from Glimepiride to Novolog correction scale per hospital protocol
Kidney failure is now stage 4-5 & planned for PD in the future
He is discharged and all home meds are restarted: Amlodipine, Aspirin, Hydralazine, Metoprolol, Tramadol PRN, Glimepiride
Wait…what? Is there anything to consider when restarting all his home meds?
17

## Slide 18
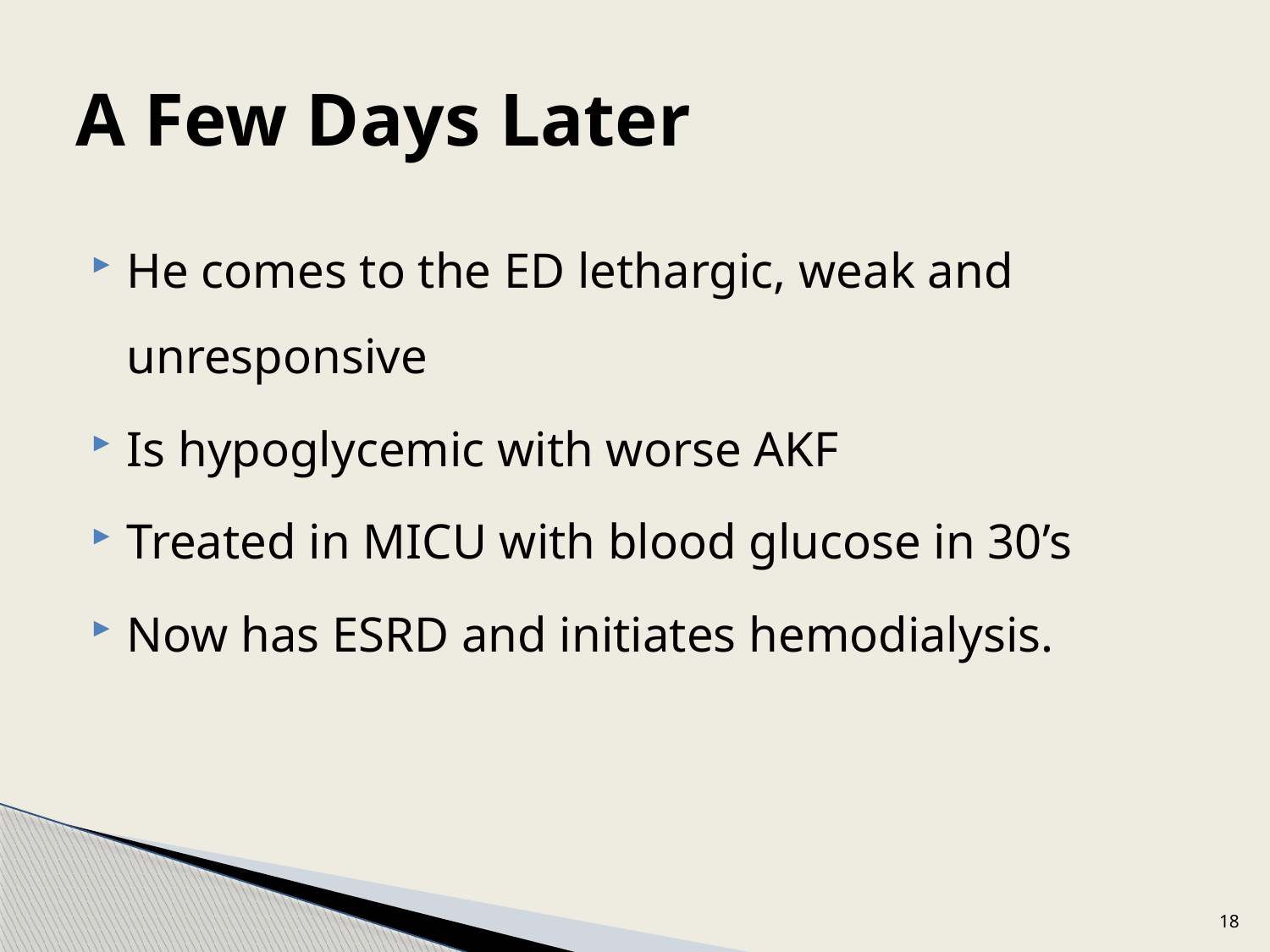

# A Few Days Later
He comes to the ED lethargic, weak and unresponsive
Is hypoglycemic with worse AKF
Treated in MICU with blood glucose in 30’s
Now has ESRD and initiates hemodialysis.
18

## Slide 19
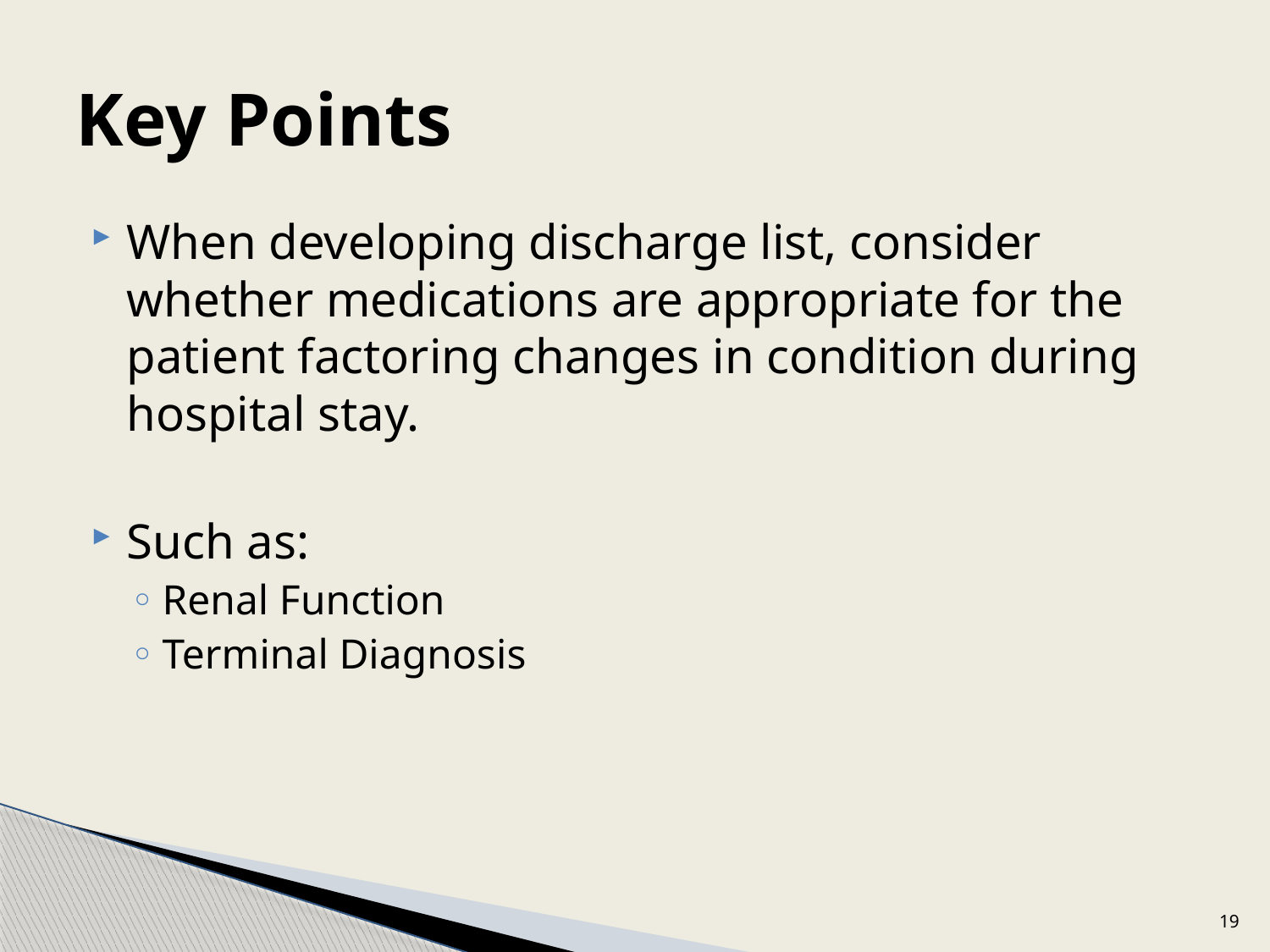

# Key Points
When developing discharge list, consider whether medications are appropriate for the patient factoring changes in condition during hospital stay.
Such as:
Renal Function
Terminal Diagnosis
19

## Slide 20
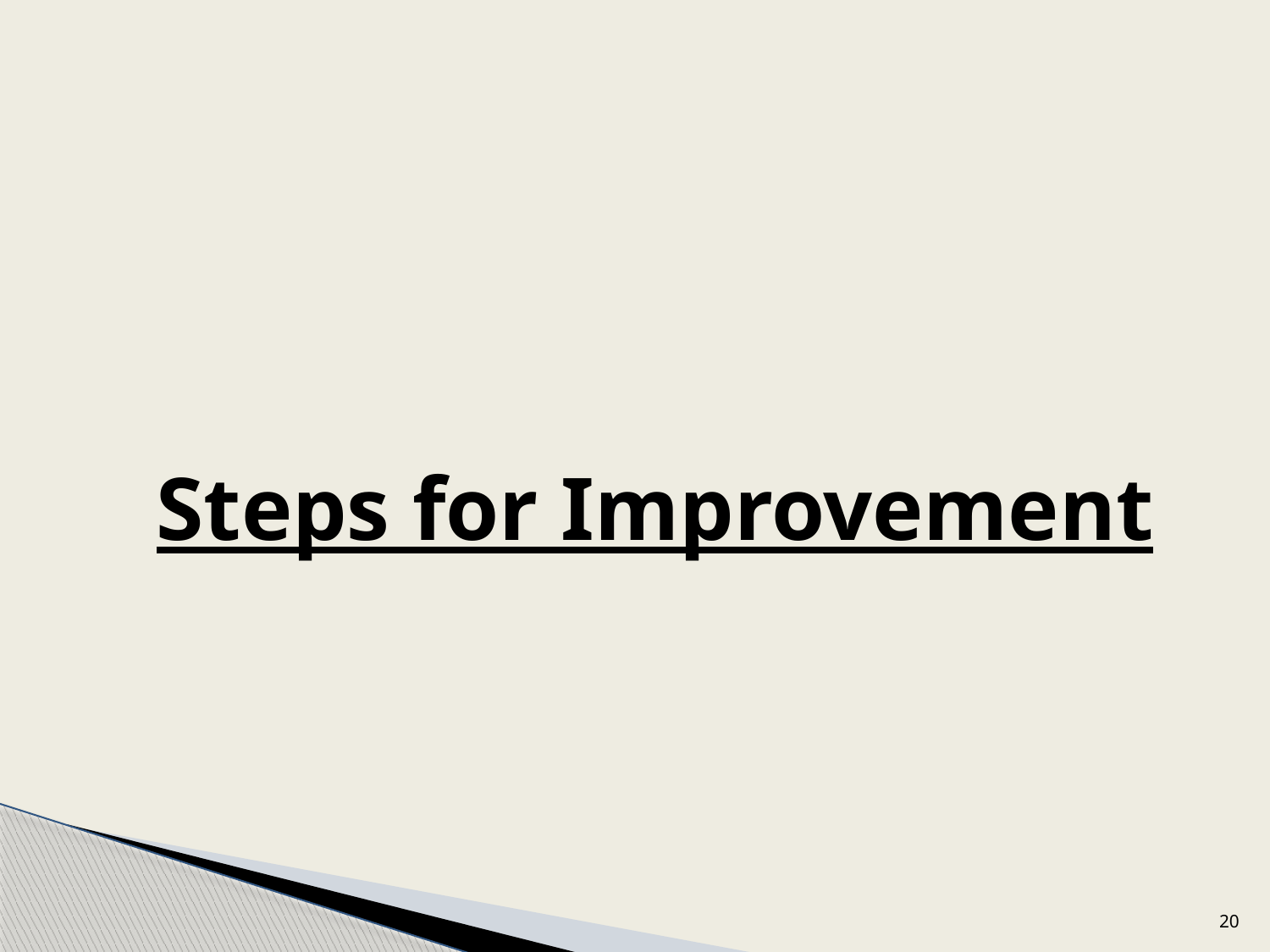

# Steps for Improvement
20

## Slide 21
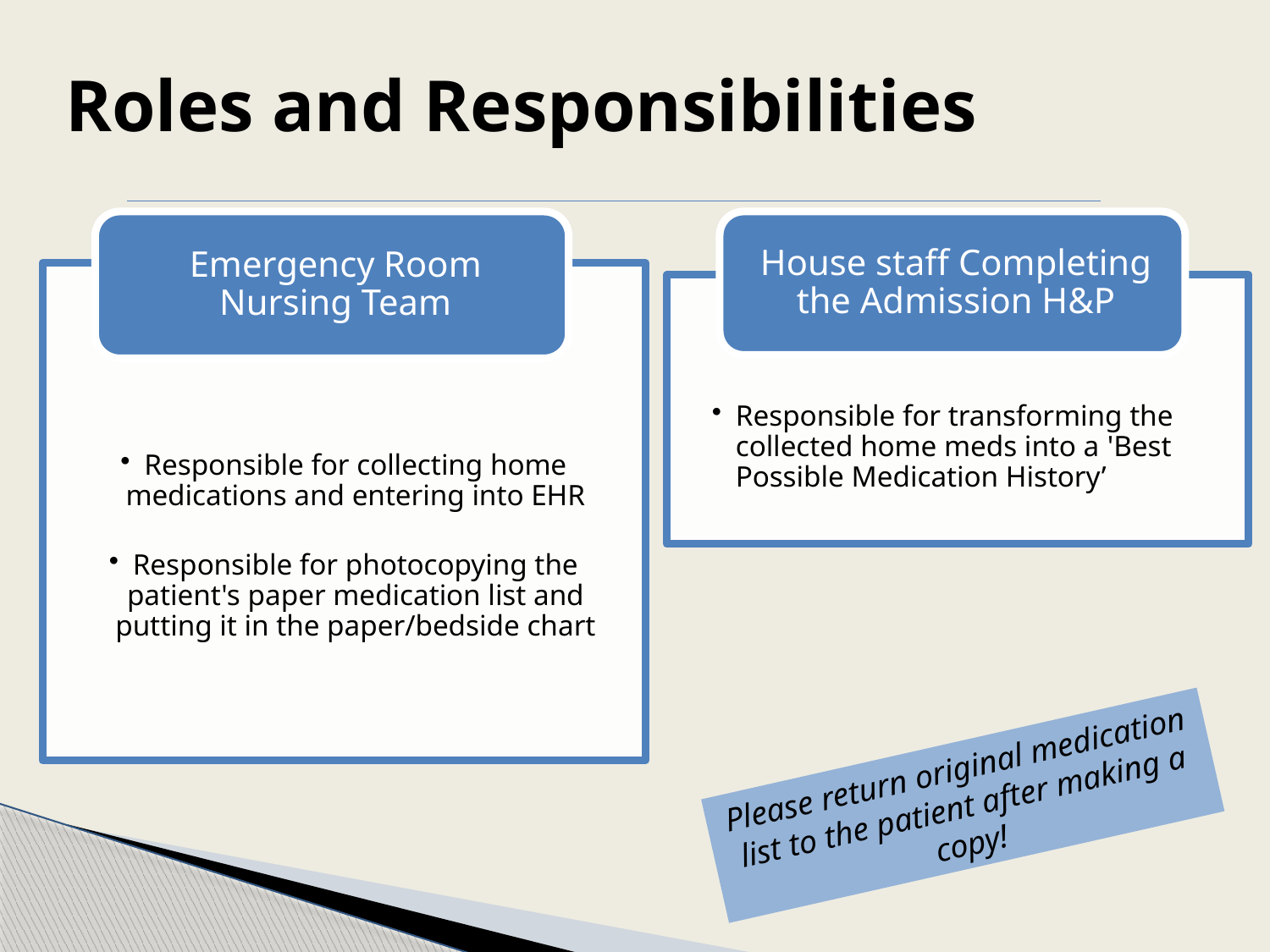

# Roles and Responsibilities
Please return original medication list to the patient after making a copy!

## Slide 22
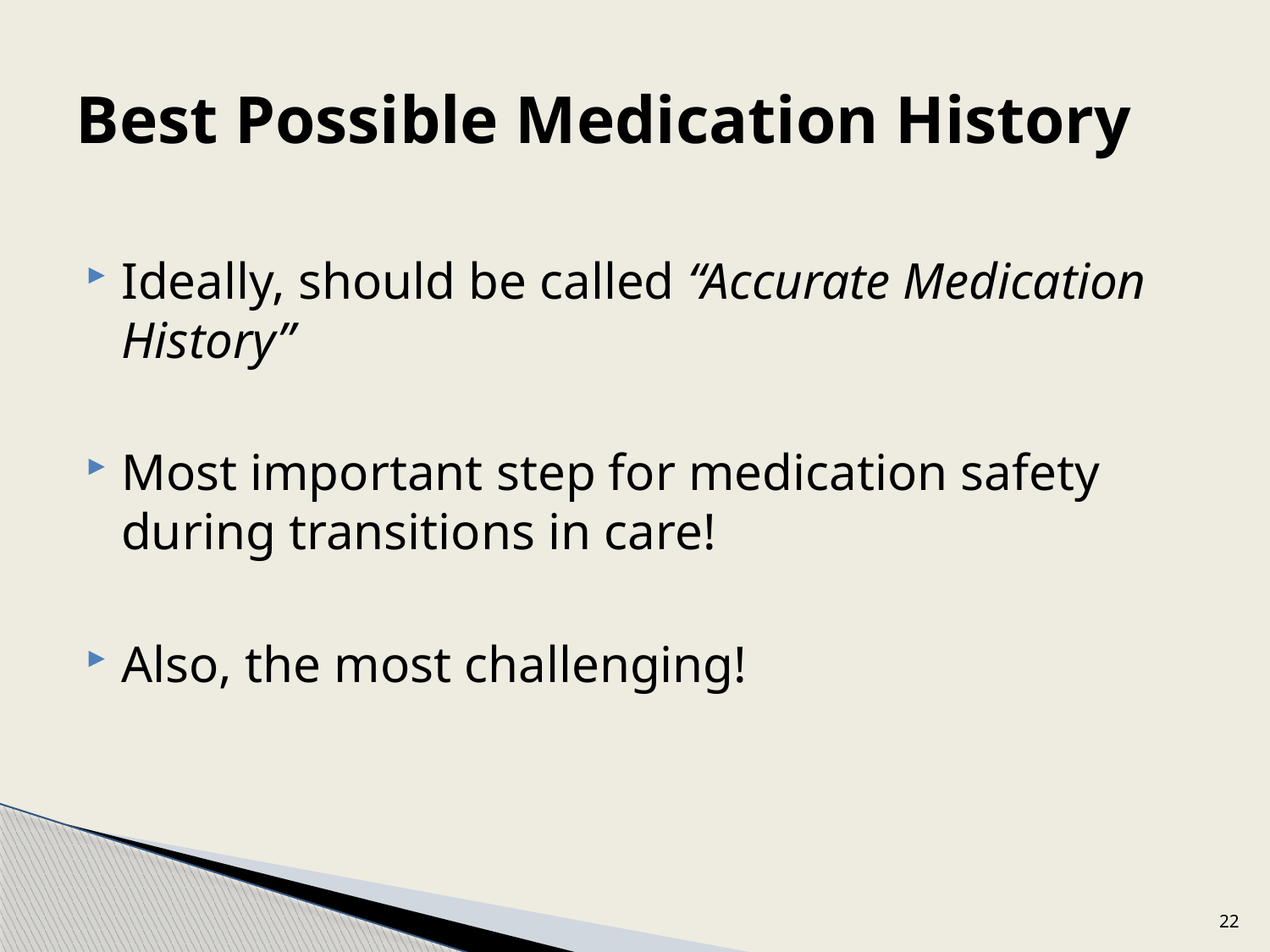

# Best Possible Medication History
Ideally, should be called “Accurate Medication History”
Most important step for medication safety during transitions in care!
Also, the most challenging!
22

## Slide 23
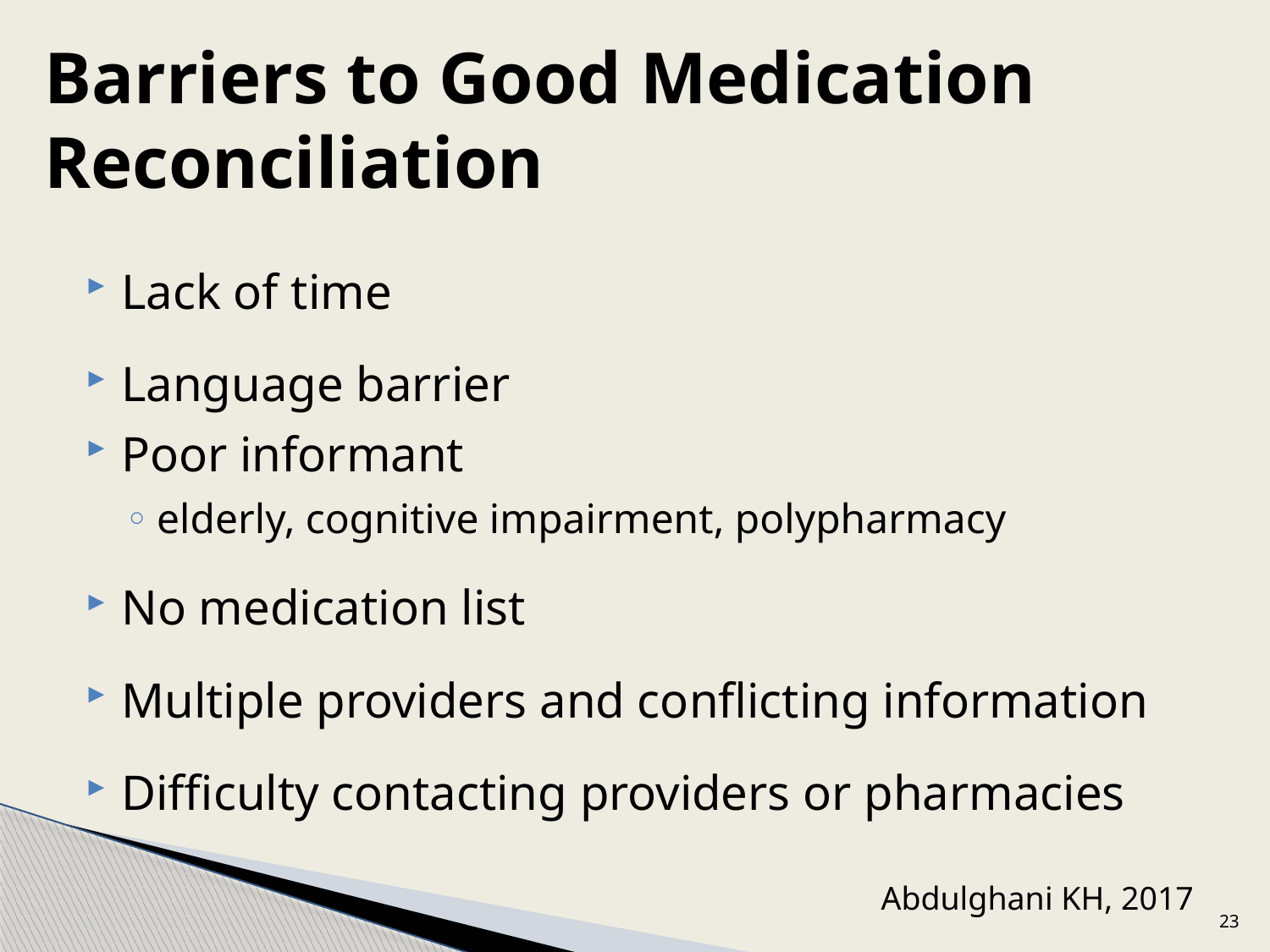

# Barriers to Good Medication Reconciliation
Lack of time
Language barrier
Poor informant
elderly, cognitive impairment, polypharmacy
No medication list
Multiple providers and conflicting information
Difficulty contacting providers or pharmacies
Abdulghani KH, 2017
23

## Slide 24
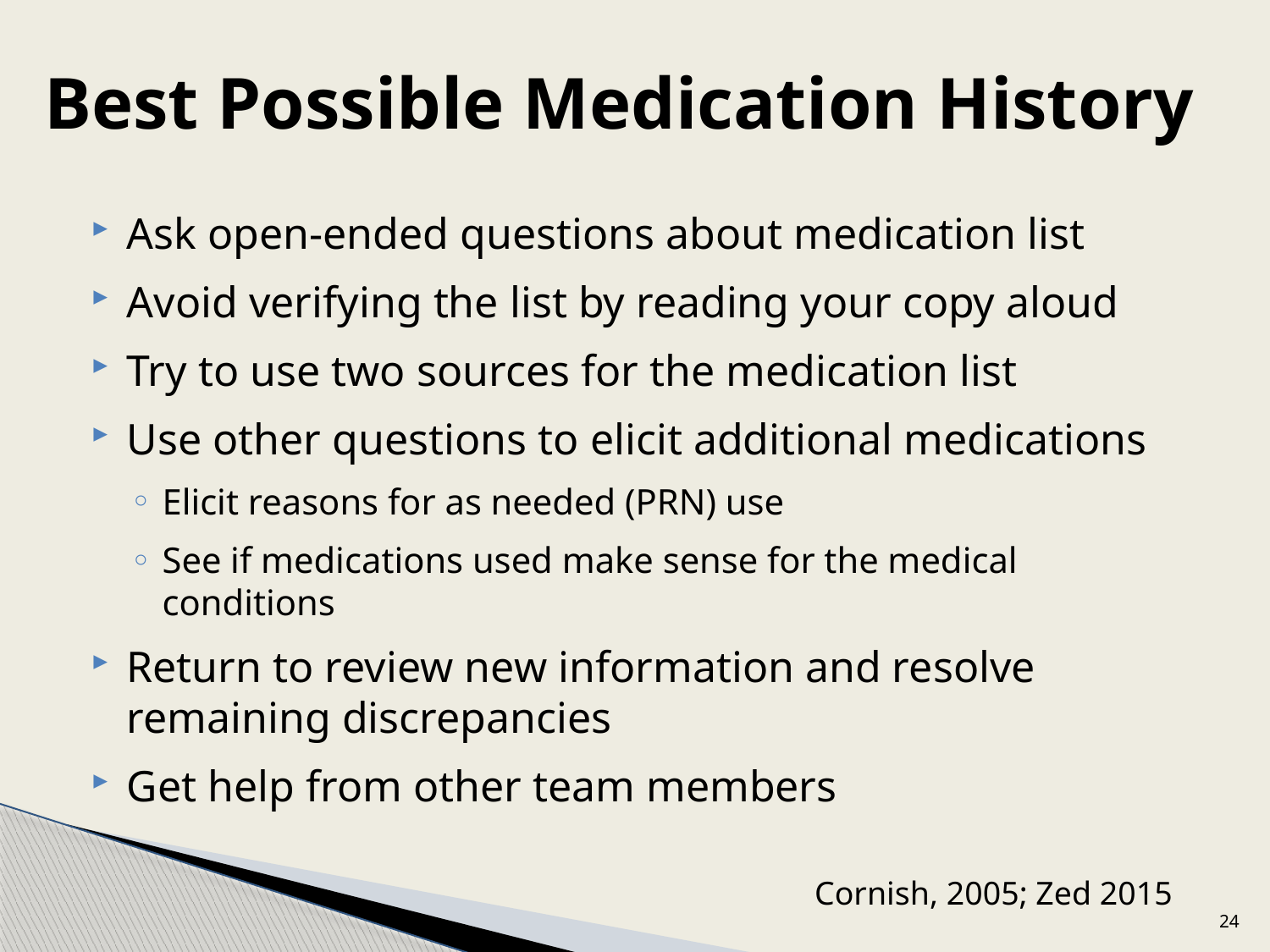

# Best Possible Medication History
Ask open-ended questions about medication list
Avoid verifying the list by reading your copy aloud
Try to use two sources for the medication list
Use other questions to elicit additional medications
Elicit reasons for as needed (PRN) use
See if medications used make sense for the medical conditions
Return to review new information and resolve remaining discrepancies
Get help from other team members
Cornish, 2005; Zed 2015
24

## Slide 25
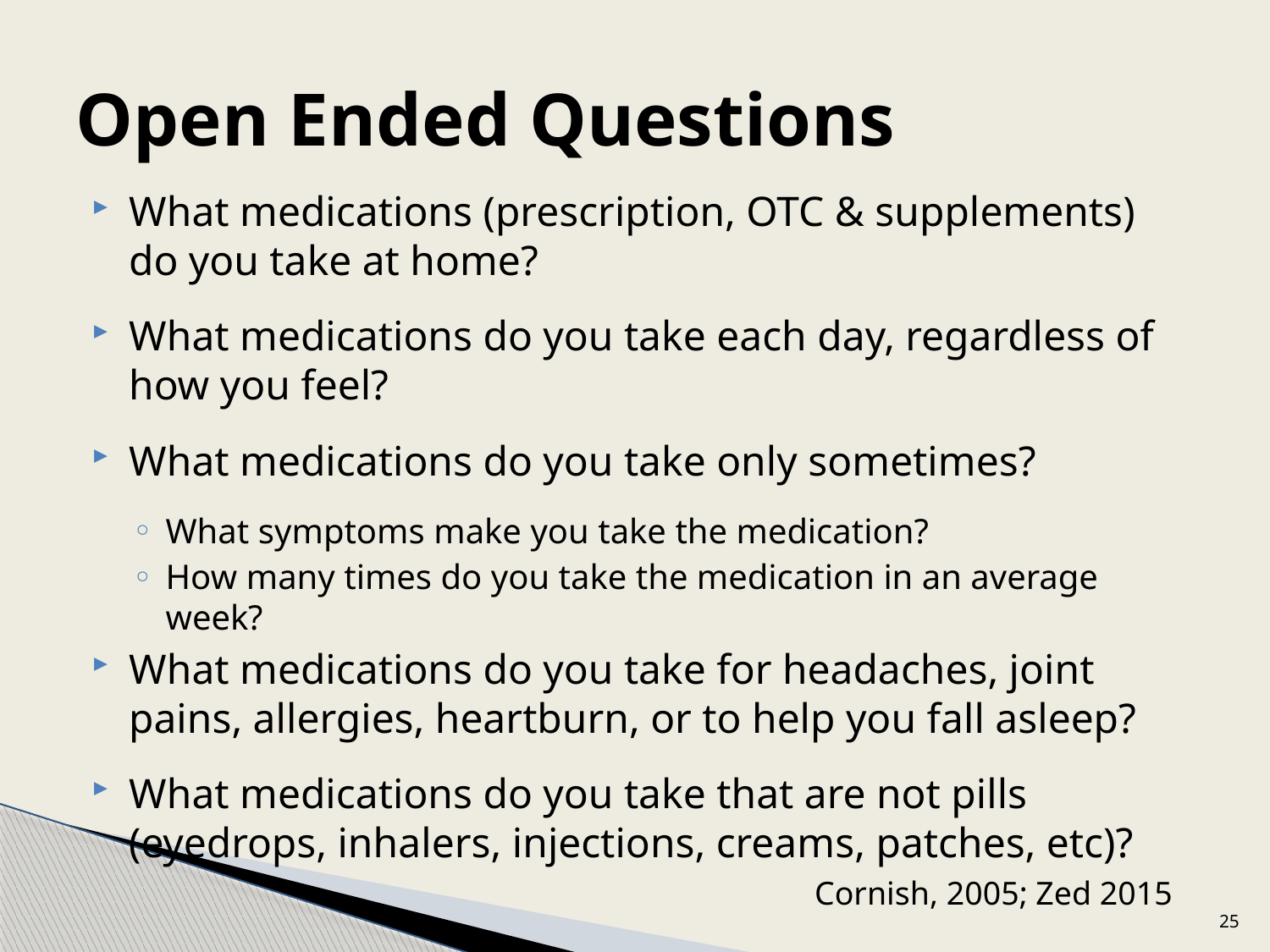

# Open Ended Questions
What medications (prescription, OTC & supplements) do you take at home?
What medications do you take each day, regardless of how you feel?
What medications do you take only sometimes?
What symptoms make you take the medication?
How many times do you take the medication in an average week?
What medications do you take for headaches, joint pains, allergies, heartburn, or to help you fall asleep?
What medications do you take that are not pills (eyedrops, inhalers, injections, creams, patches, etc)?
Cornish, 2005; Zed 2015
25

## Slide 26
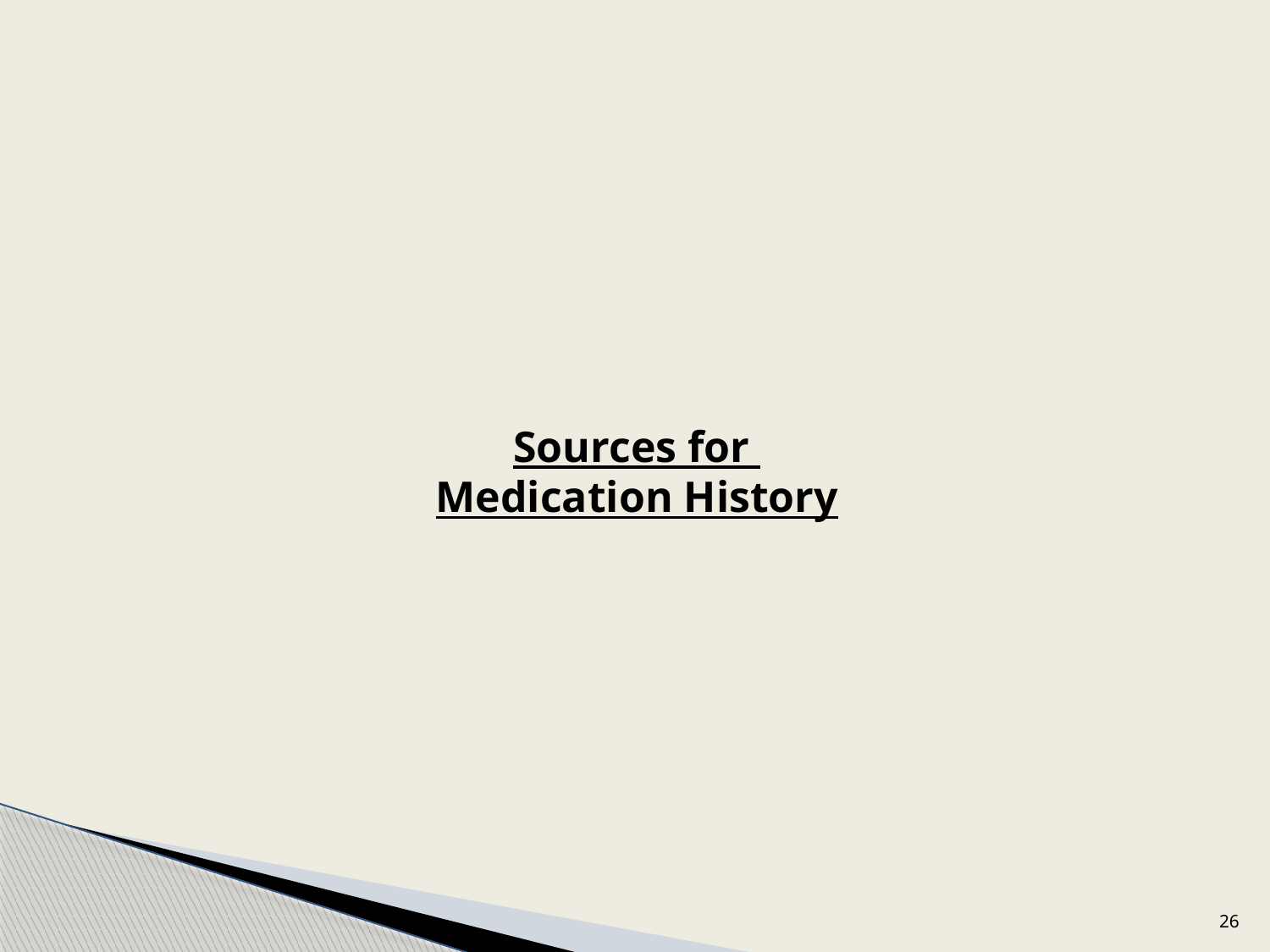

# Sources for Medication History
26

## Slide 27
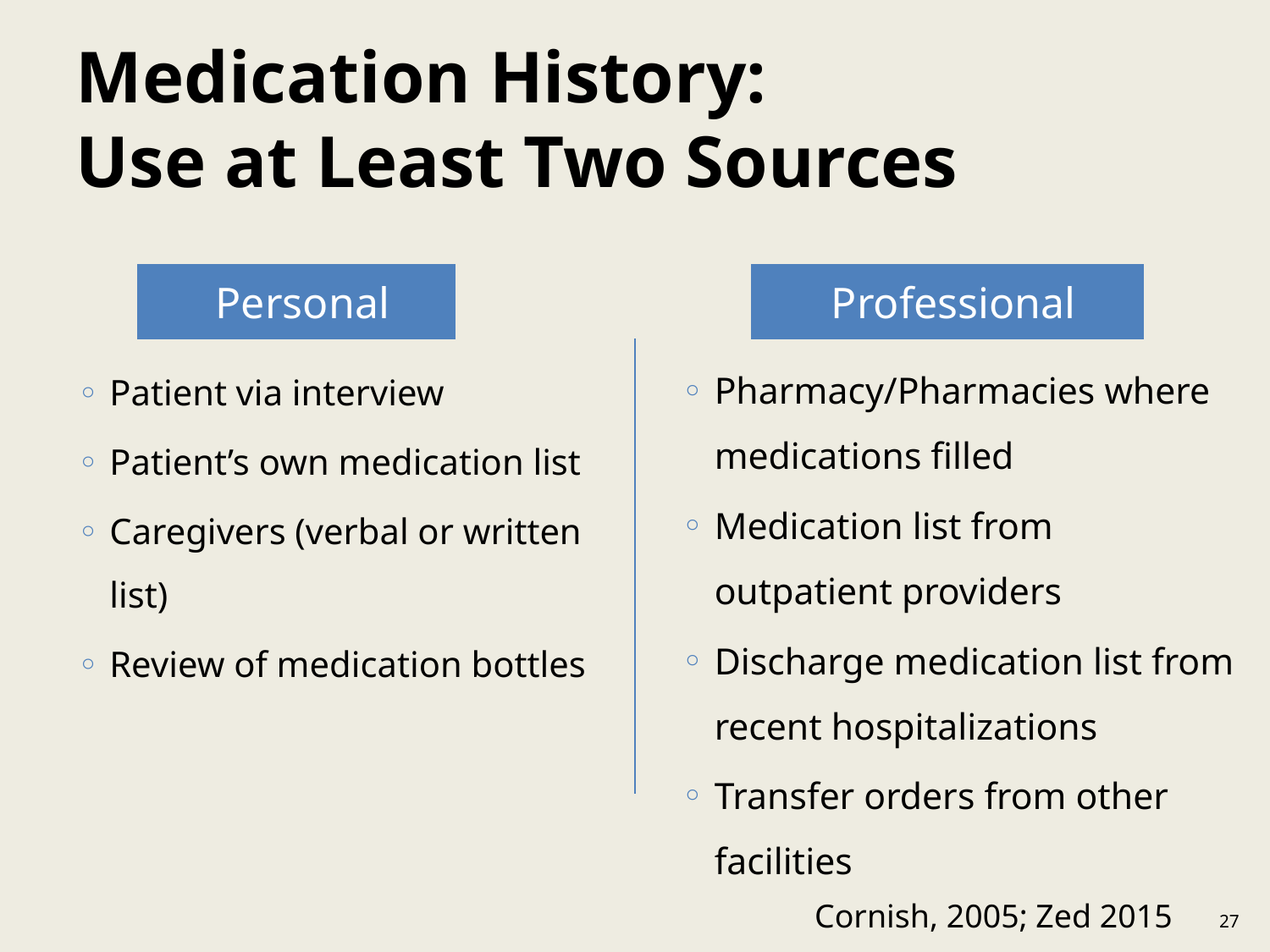

# Medication History: Use at Least Two Sources
Personal
Professional
Pharmacy/Pharmacies where medications filled
Medication list from outpatient providers
Discharge medication list from recent hospitalizations
Transfer orders from other facilities
Patient via interview
Patient’s own medication list
Caregivers (verbal or written list)
Review of medication bottles
27
Cornish, 2005; Zed 2015

## Slide 28
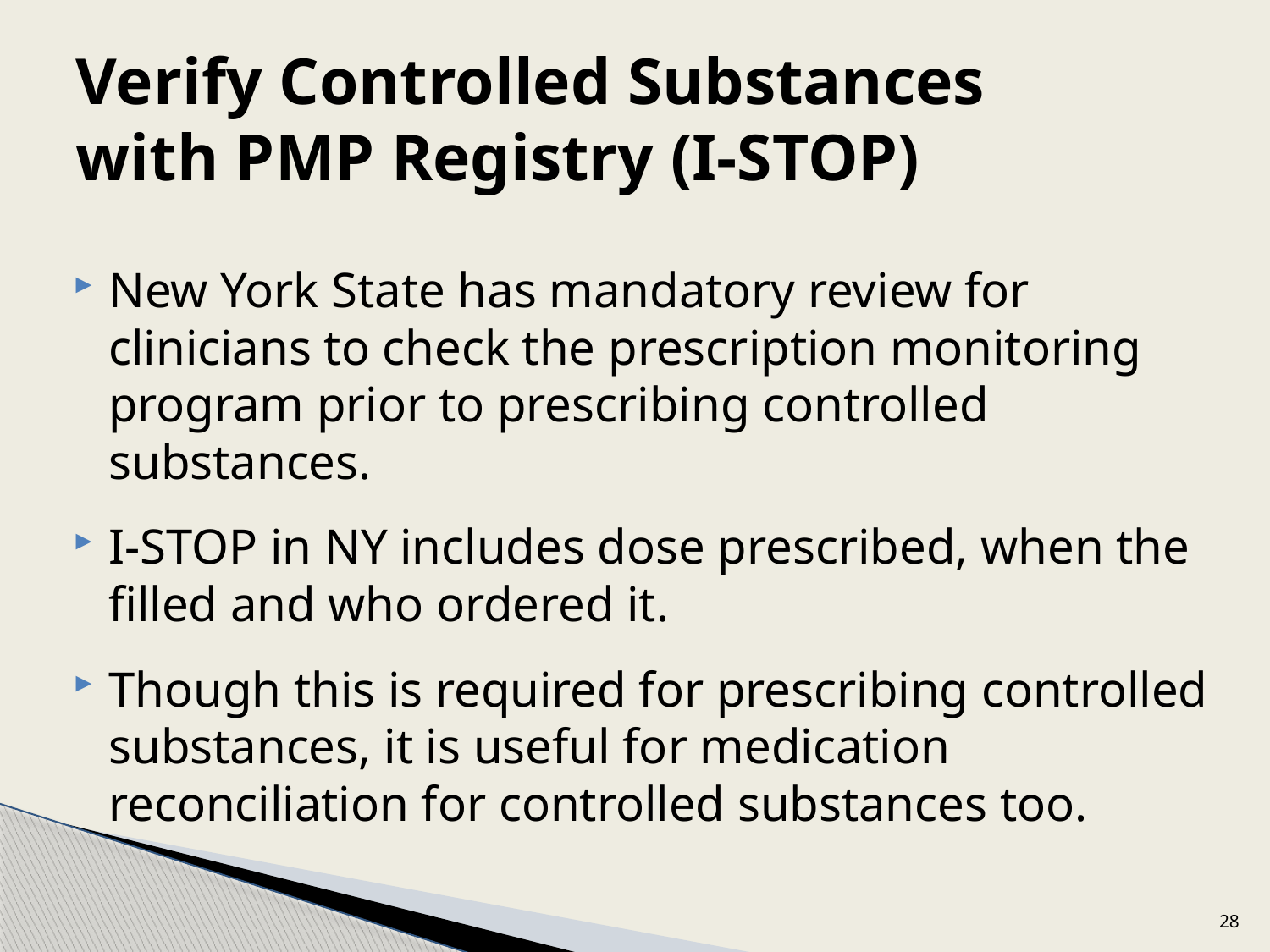

# Verify Controlled Substances with PMP Registry (I-STOP)
New York State has mandatory review for clinicians to check the prescription monitoring program prior to prescribing controlled substances.
I-STOP in NY includes dose prescribed, when the filled and who ordered it.
Though this is required for prescribing controlled substances, it is useful for medication reconciliation for controlled substances too.
28

## Slide 29
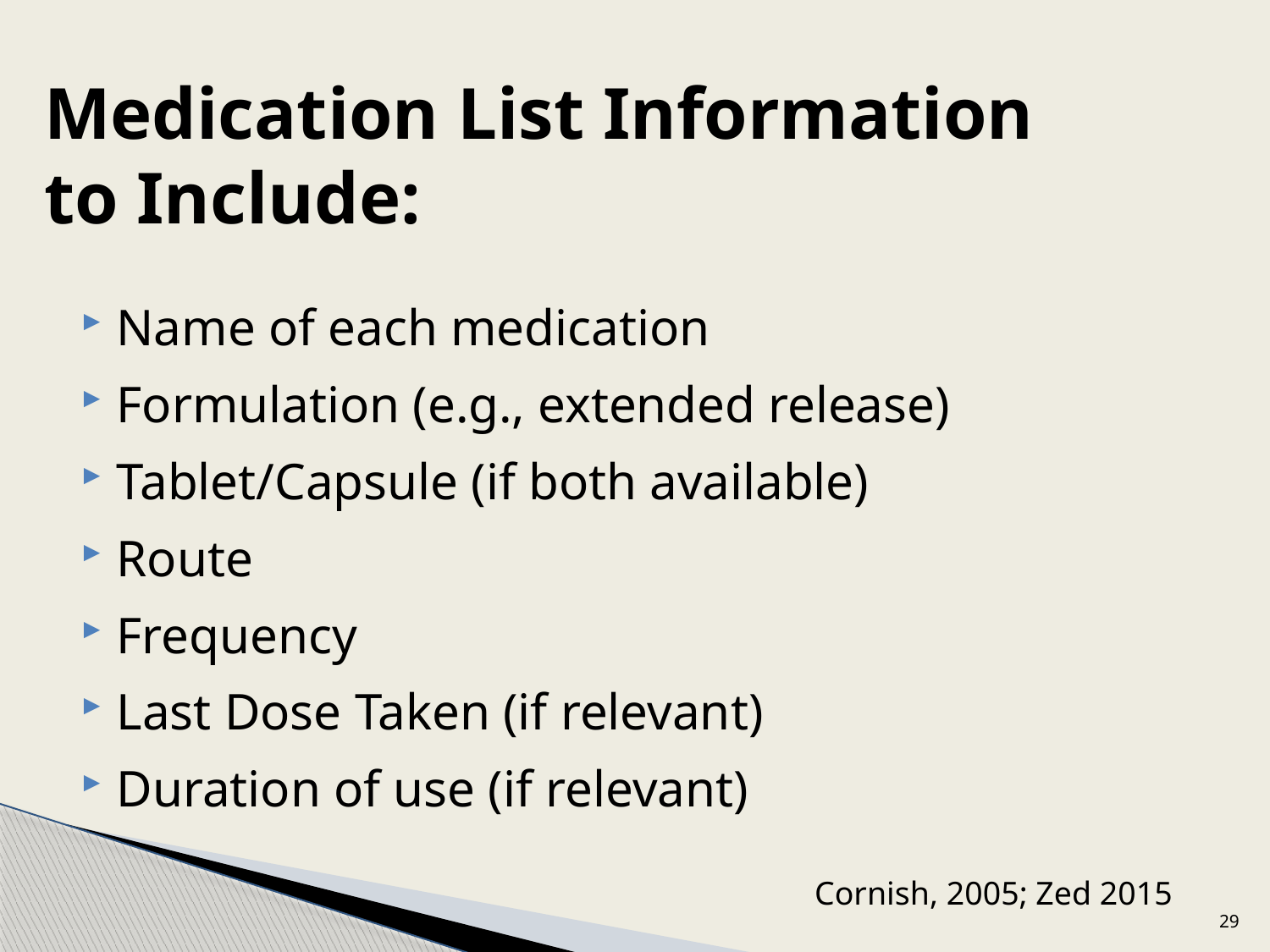

# Medication List Information to Include:
Name of each medication
Formulation (e.g., extended release)
Tablet/Capsule (if both available)
Route
Frequency
Last Dose Taken (if relevant)
Duration of use (if relevant)
Cornish, 2005; Zed 2015
29

## Slide 30
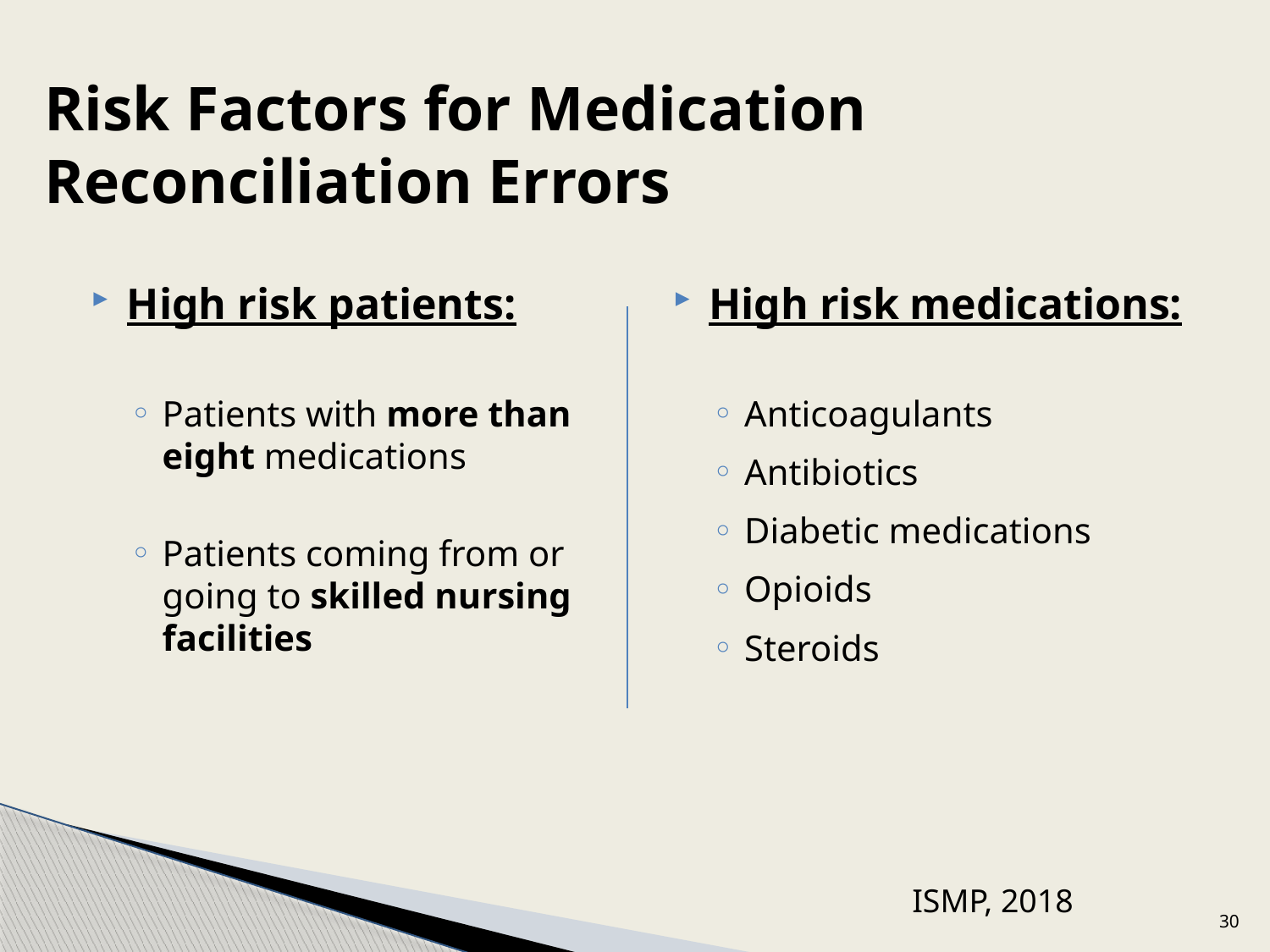

# Risk Factors for Medication Reconciliation Errors
High risk patients:
Patients with more than eight medications
Patients coming from or going to skilled nursing facilities
High risk medications:
Anticoagulants
Antibiotics
Diabetic medications
Opioids
Steroids
ISMP, 2018
30

## Slide 31
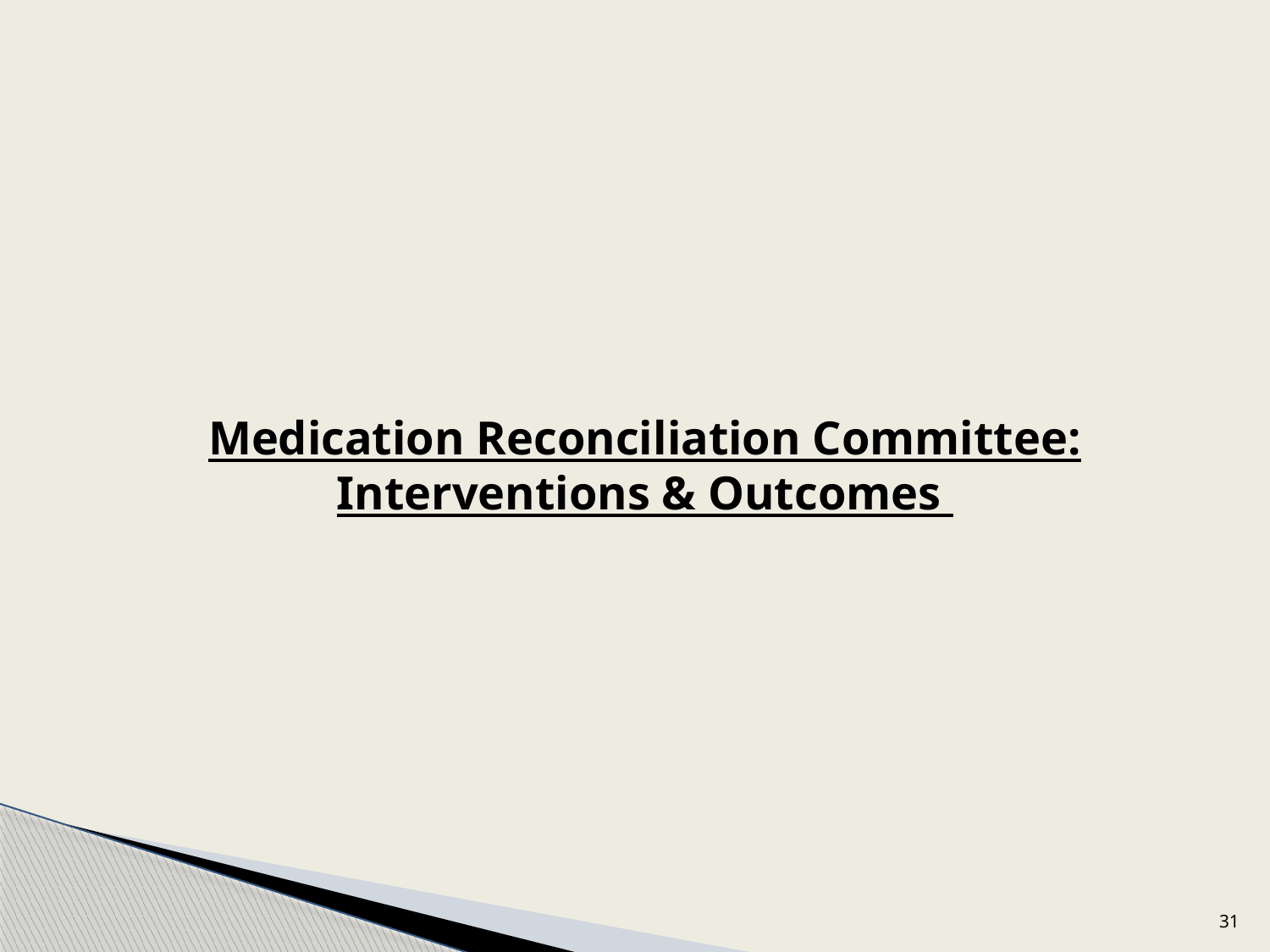

# Medication Reconciliation Committee: Interventions & Outcomes
31

## Slide 32
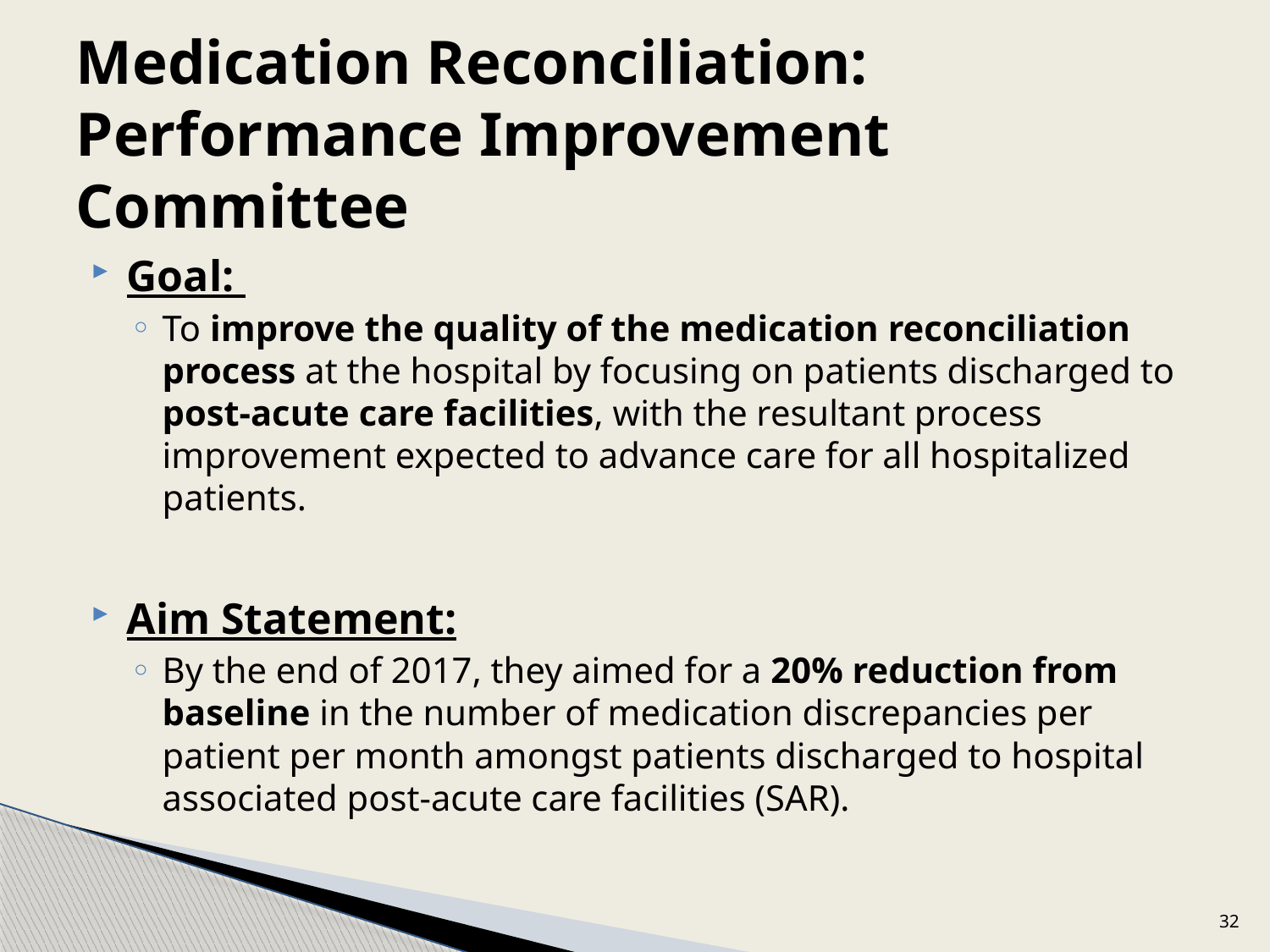

# Medication Reconciliation: Performance Improvement Committee
Goal:
To improve the quality of the medication reconciliation process at the hospital by focusing on patients discharged to post-acute care facilities, with the resultant process improvement expected to advance care for all hospitalized patients.
Aim Statement:
By the end of 2017, they aimed for a 20% reduction from baseline in the number of medication discrepancies per patient per month amongst patients discharged to hospital associated post-acute care facilities (SAR).
32

## Slide 33
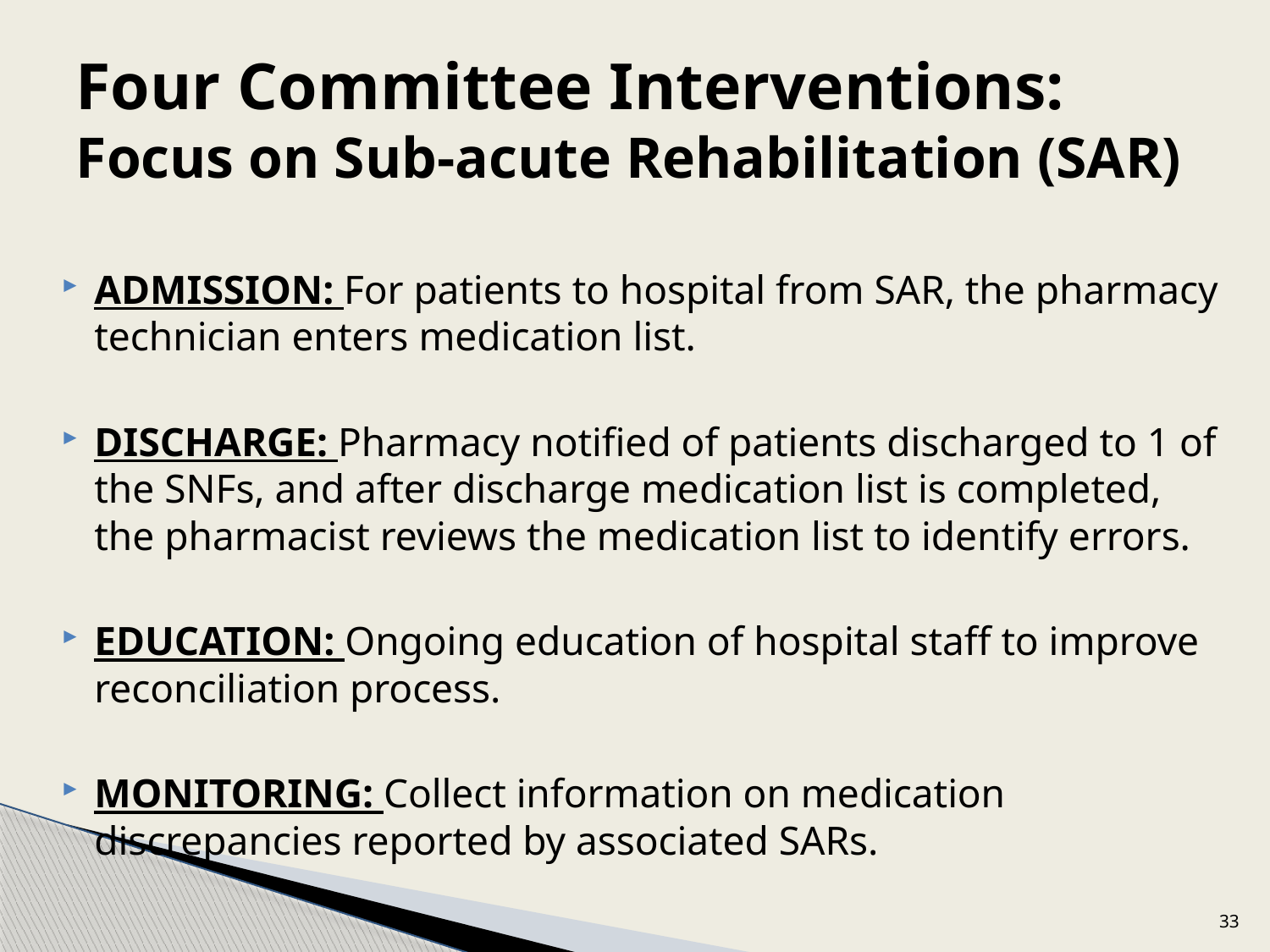

# Four Committee Interventions: Focus on Sub-acute Rehabilitation (SAR)
ADMISSION: For patients to hospital from SAR, the pharmacy technician enters medication list.
DISCHARGE: Pharmacy notified of patients discharged to 1 of the SNFs, and after discharge medication list is completed, the pharmacist reviews the medication list to identify errors.
EDUCATION: Ongoing education of hospital staff to improve reconciliation process.
MONITORING: Collect information on medication discrepancies reported by associated SARs.
33

## Slide 34
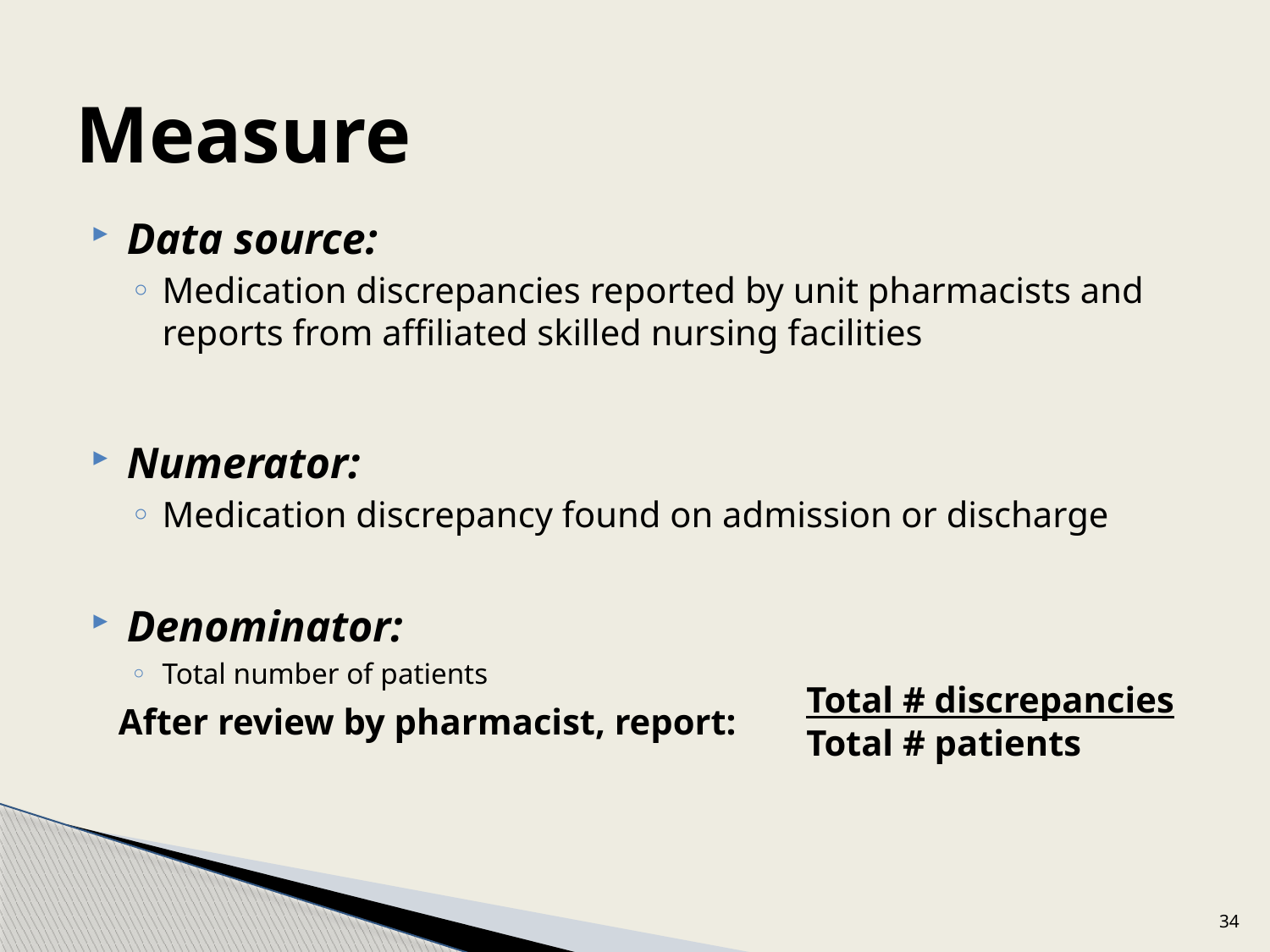

# Measure
Data source:
Medication discrepancies reported by unit pharmacists and reports from affiliated skilled nursing facilities
Numerator:
Medication discrepancy found on admission or discharge
Denominator:
Total number of patients
Total # discrepancies
Total # patients
After review by pharmacist, report:
34

## Slide 35
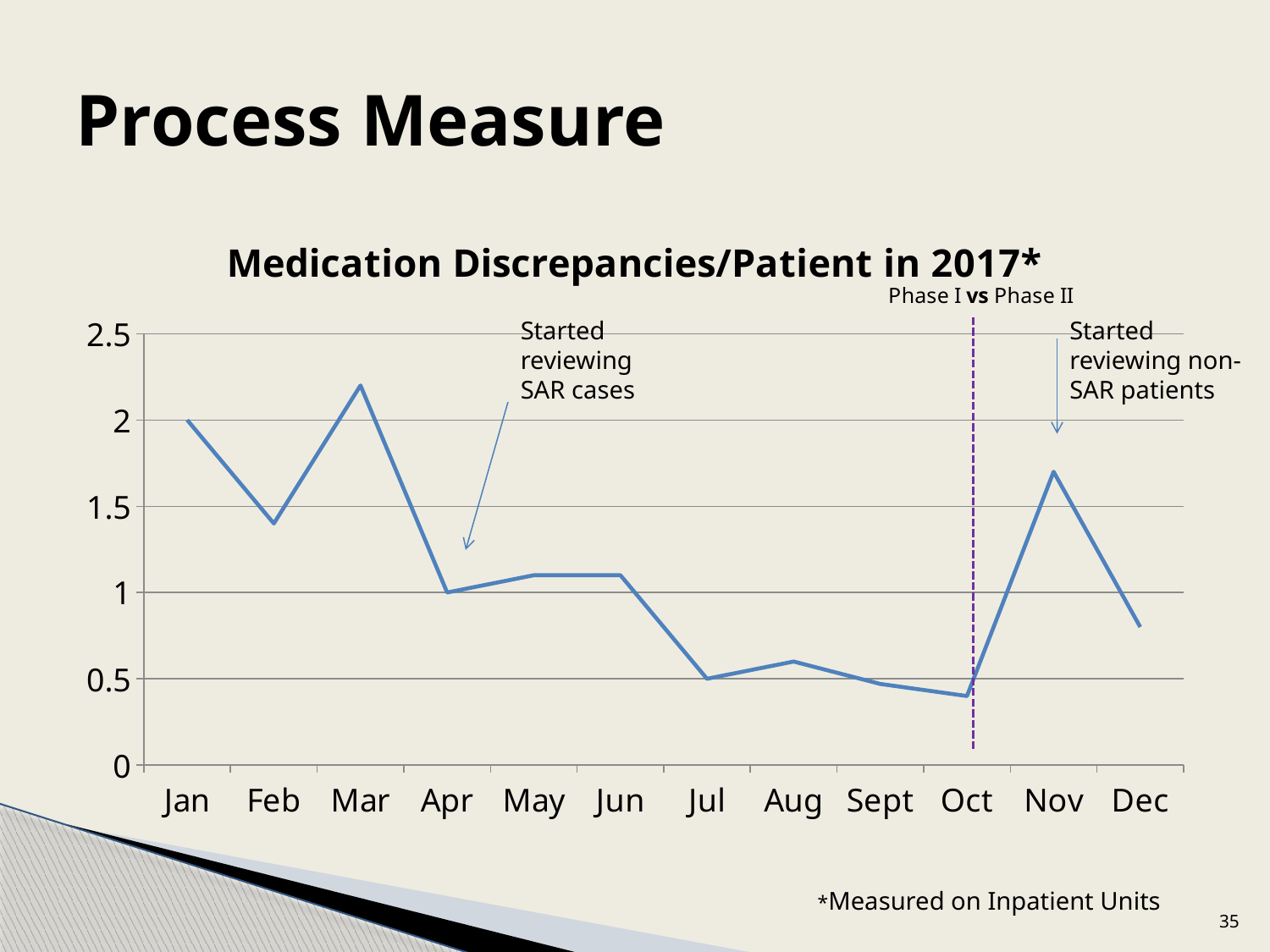

# Process Measure
### Chart: Medication Discrepancies/Patient in 2017*
| Category | Med discrepancies/patient 2017 |
|---|---|
| Jan | 2.0 |
| Feb | 1.4 |
| Mar | 2.2 |
| Apr | 1.0 |
| May | 1.1 |
| Jun | 1.1 |
| Jul | 0.5 |
| Aug | 0.6 |
| Sept | 0.47 |
| Oct | 0.4 |
| Nov | 1.7 |
| Dec | 0.8 |Started reviewing SAR cases
Started reviewing non-SAR patients
*Measured on Inpatient Units
35

## Slide 36
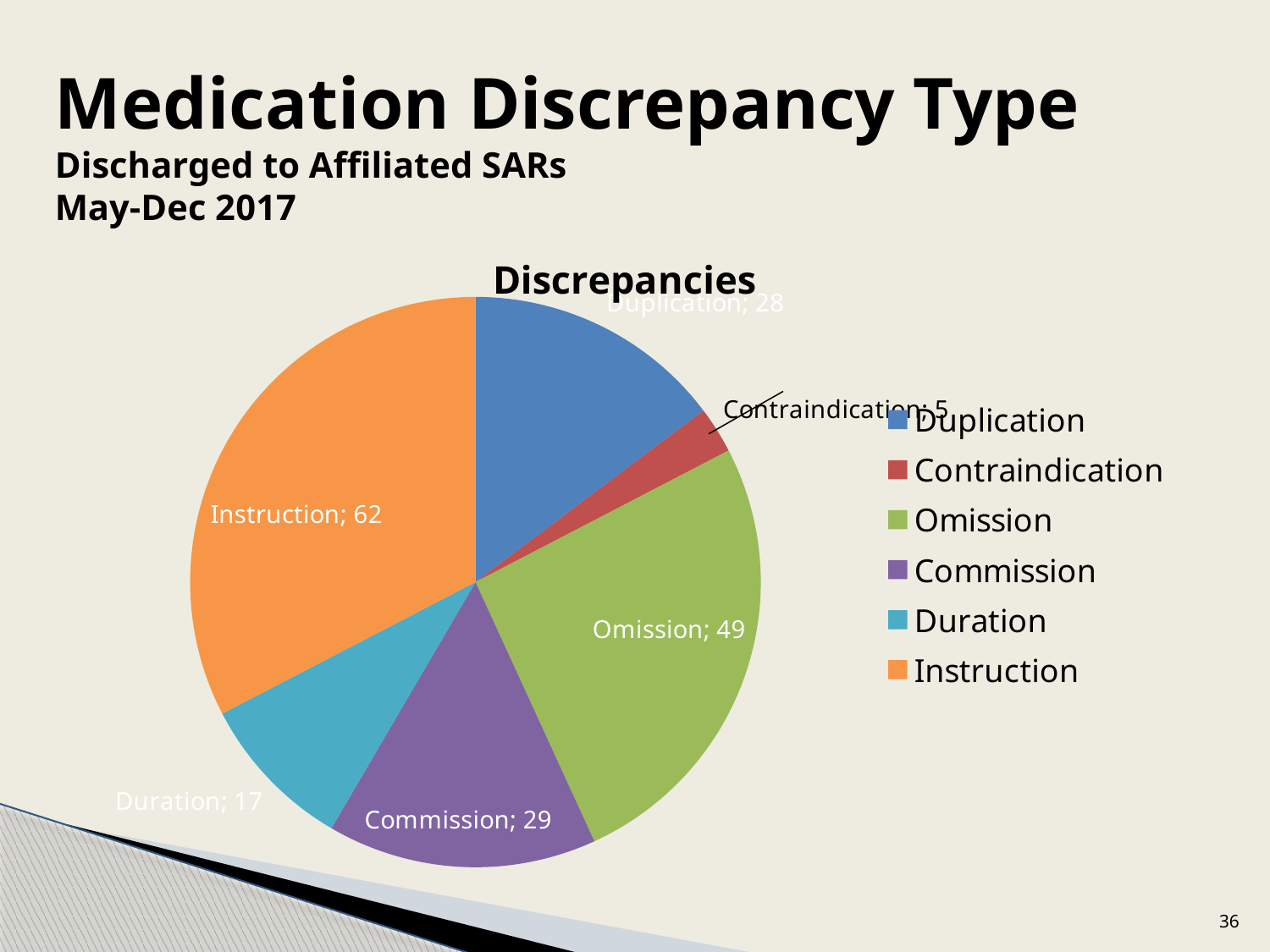

# Medication Discrepancy Type Discharged to Affiliated SARs May-Dec 2017
### Chart:
| Category | Discrepancies | Column1 | Column2 | Column3 | Column4 | Column5 | Column6 |
|---|---|---|---|---|---|---|---|
| Duplication | 28.0 | None | None | None | None | None | None |
| Contraindication | 5.0 | None | None | None | None | None | None |
| Omission | 49.0 | None | None | None | None | None | None |
| Commission | 29.0 | None | None | None | None | None | None |
| Duration | 17.0 | None | None | None | None | None | None |
| Instruction | 62.0 | None | None | None | None | None | None |36

## Slide 37
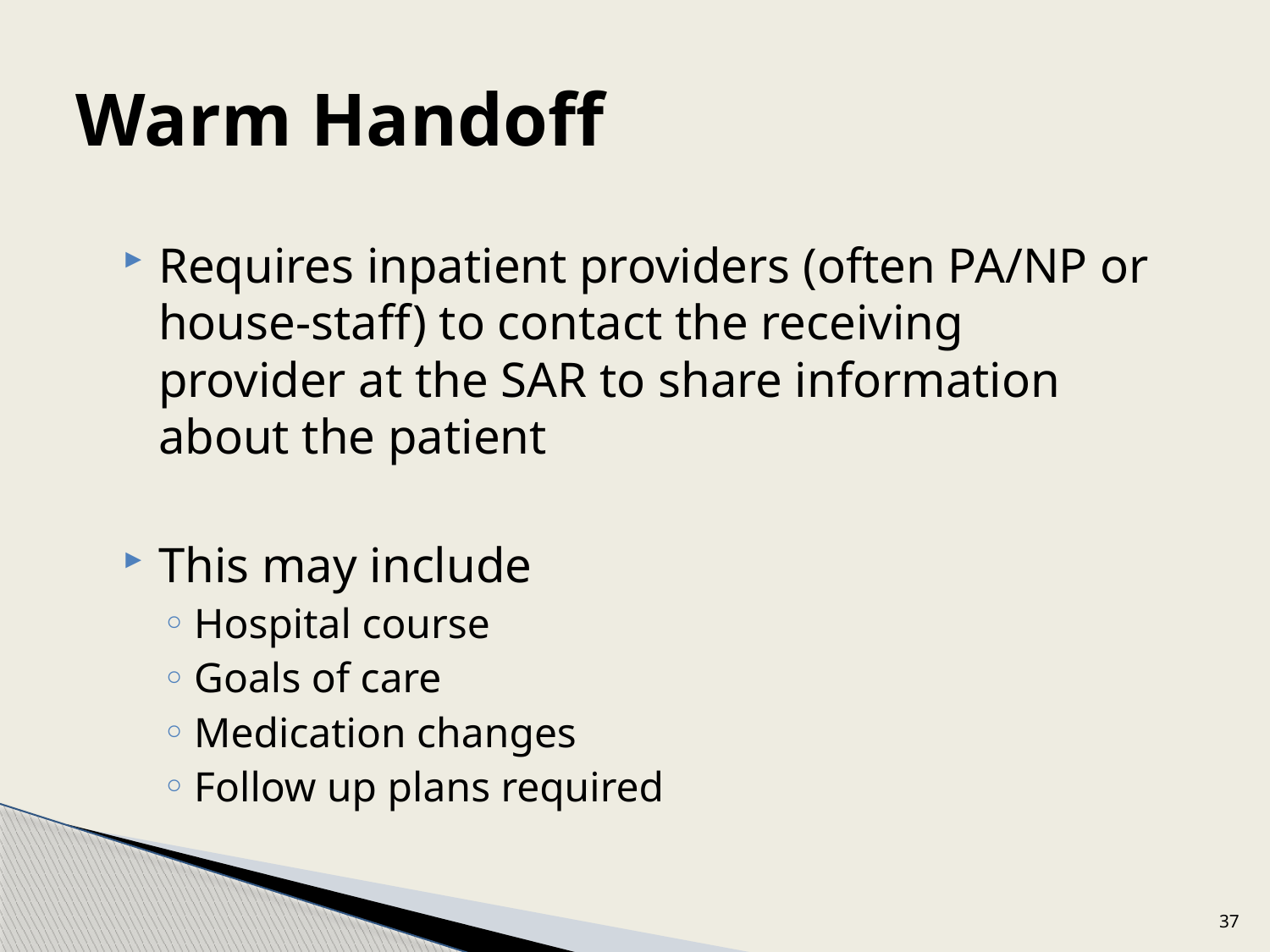

# Warm Handoff
Requires inpatient providers (often PA/NP or house-staff) to contact the receiving provider at the SAR to share information about the patient
This may include
Hospital course
Goals of care
Medication changes
Follow up plans required
37

## Slide 38
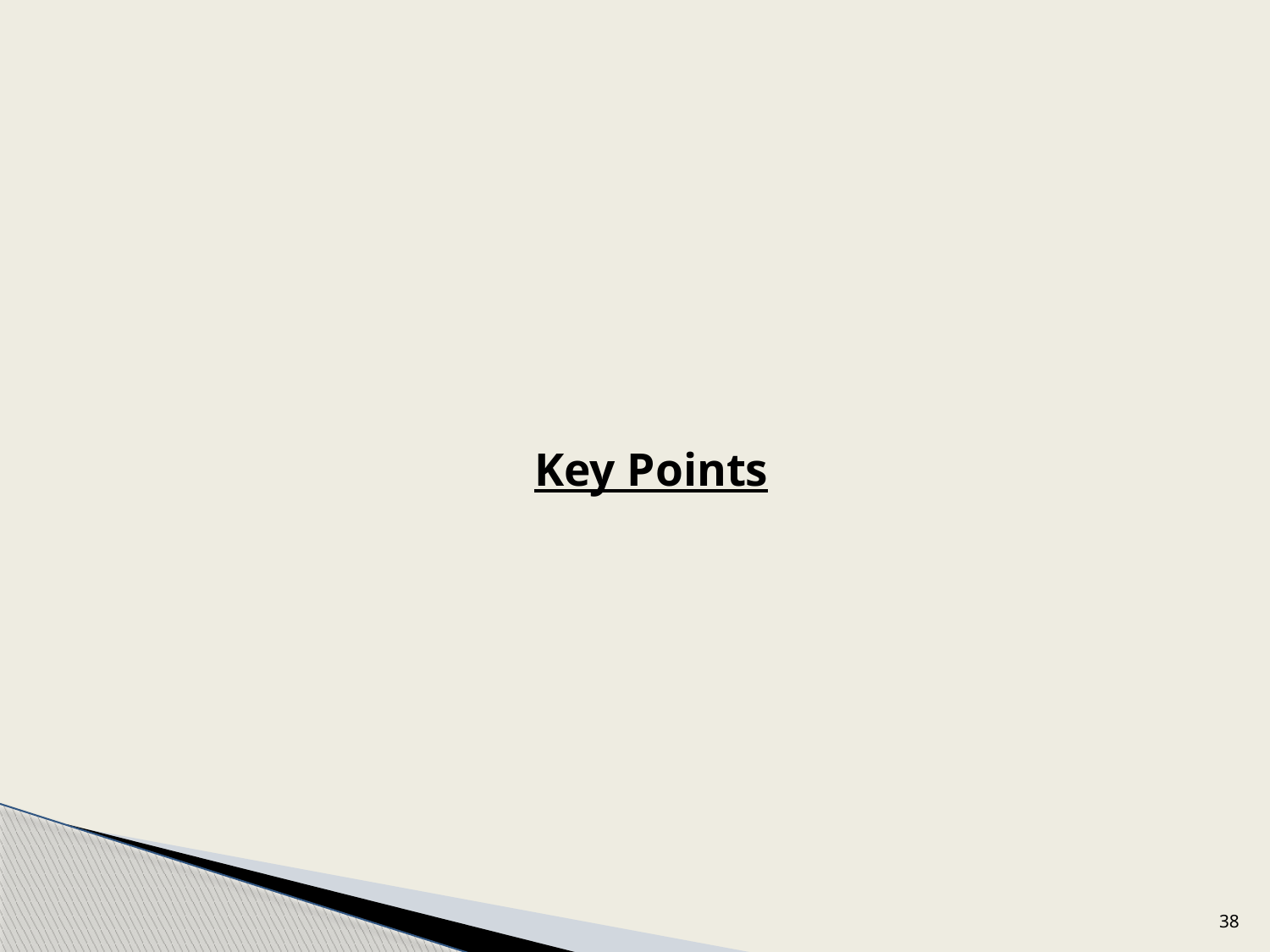

# Key Points
38

## Slide 39
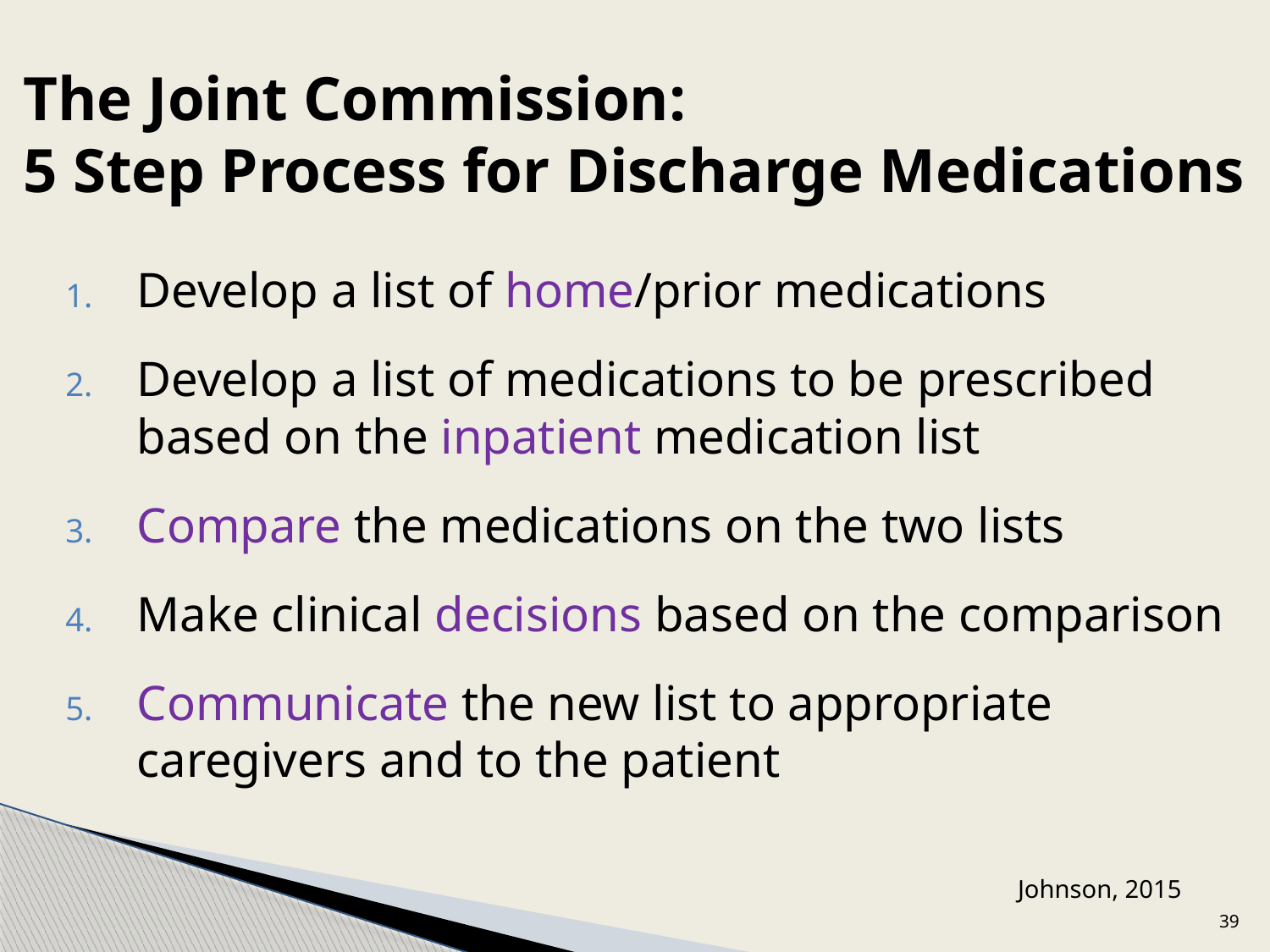

# The Joint Commission: 5 Step Process for Discharge Medications
Develop a list of home/prior medications
Develop a list of medications to be prescribed based on the inpatient medication list
Compare the medications on the two lists
Make clinical decisions based on the comparison
Communicate the new list to appropriate caregivers and to the patient
Johnson, 2015
39

## Slide 40
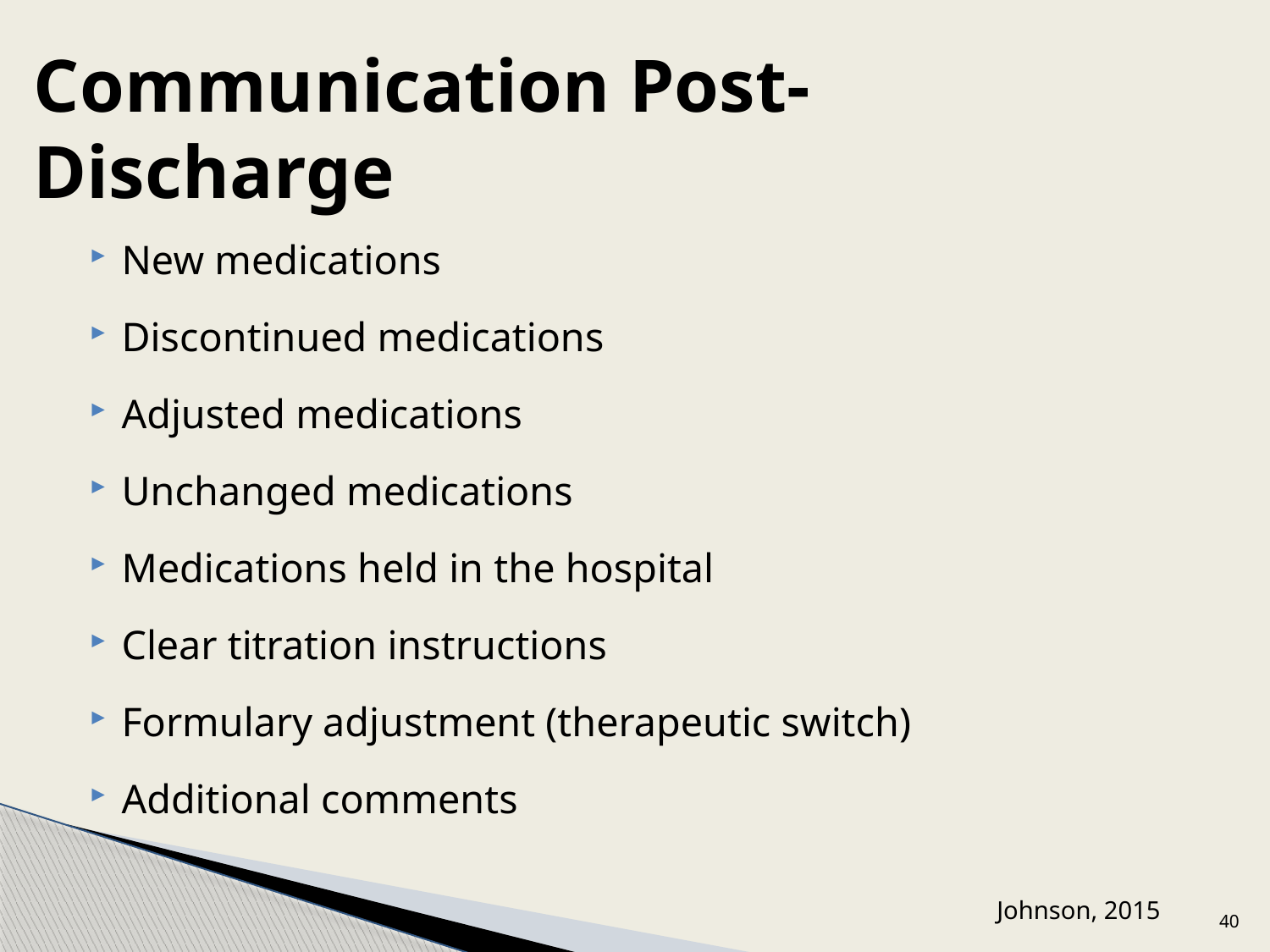

# Communication Post-Discharge
New medications
Discontinued medications
Adjusted medications
Unchanged medications
Medications held in the hospital
Clear titration instructions
Formulary adjustment (therapeutic switch)
Additional comments
Johnson, 2015
40

## Slide 41
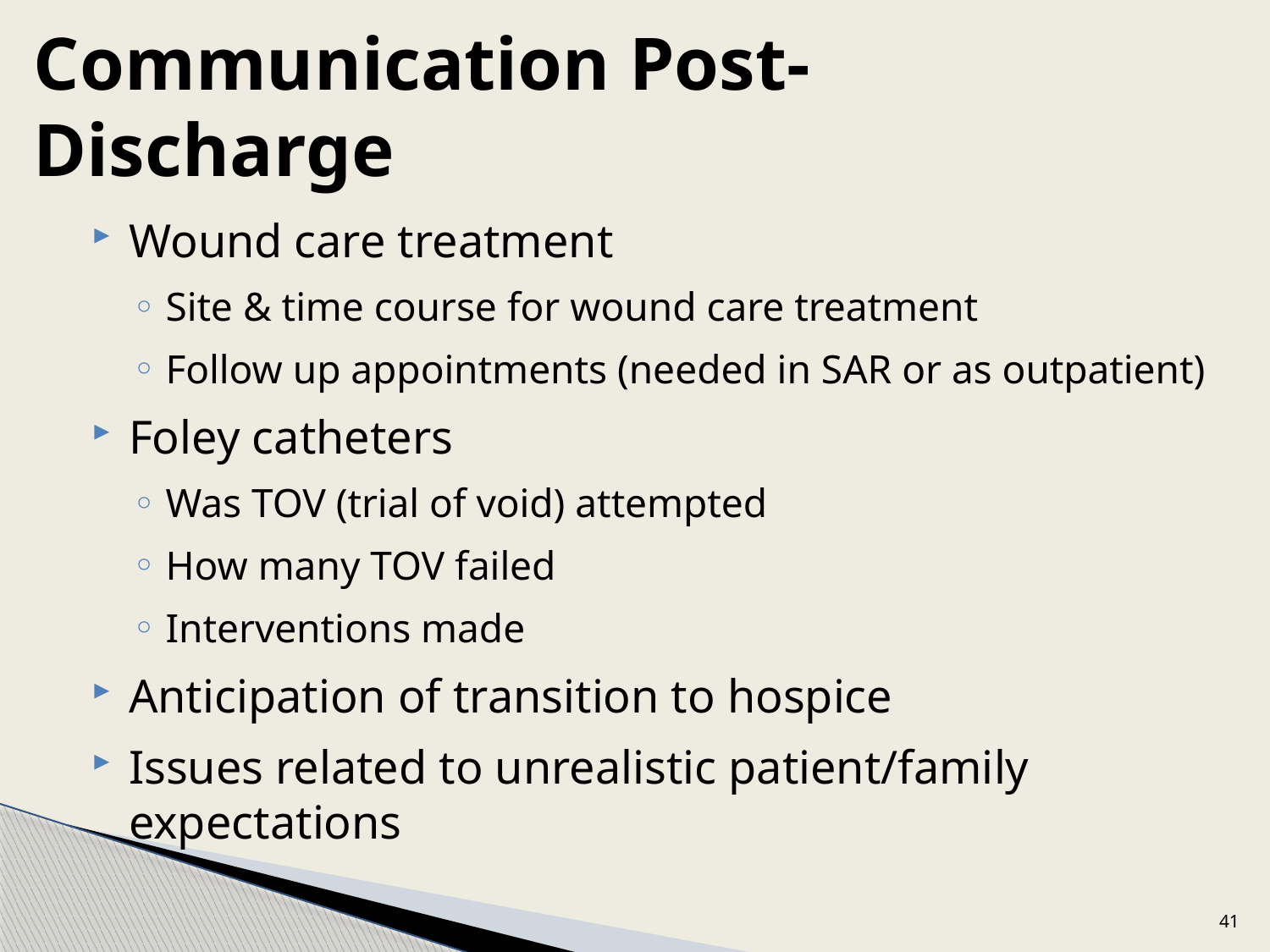

# Communication Post-Discharge
Wound care treatment
Site & time course for wound care treatment
Follow up appointments (needed in SAR or as outpatient)
Foley catheters
Was TOV (trial of void) attempted
How many TOV failed
Interventions made
Anticipation of transition to hospice
Issues related to unrealistic patient/family expectations
41

## Slide 42
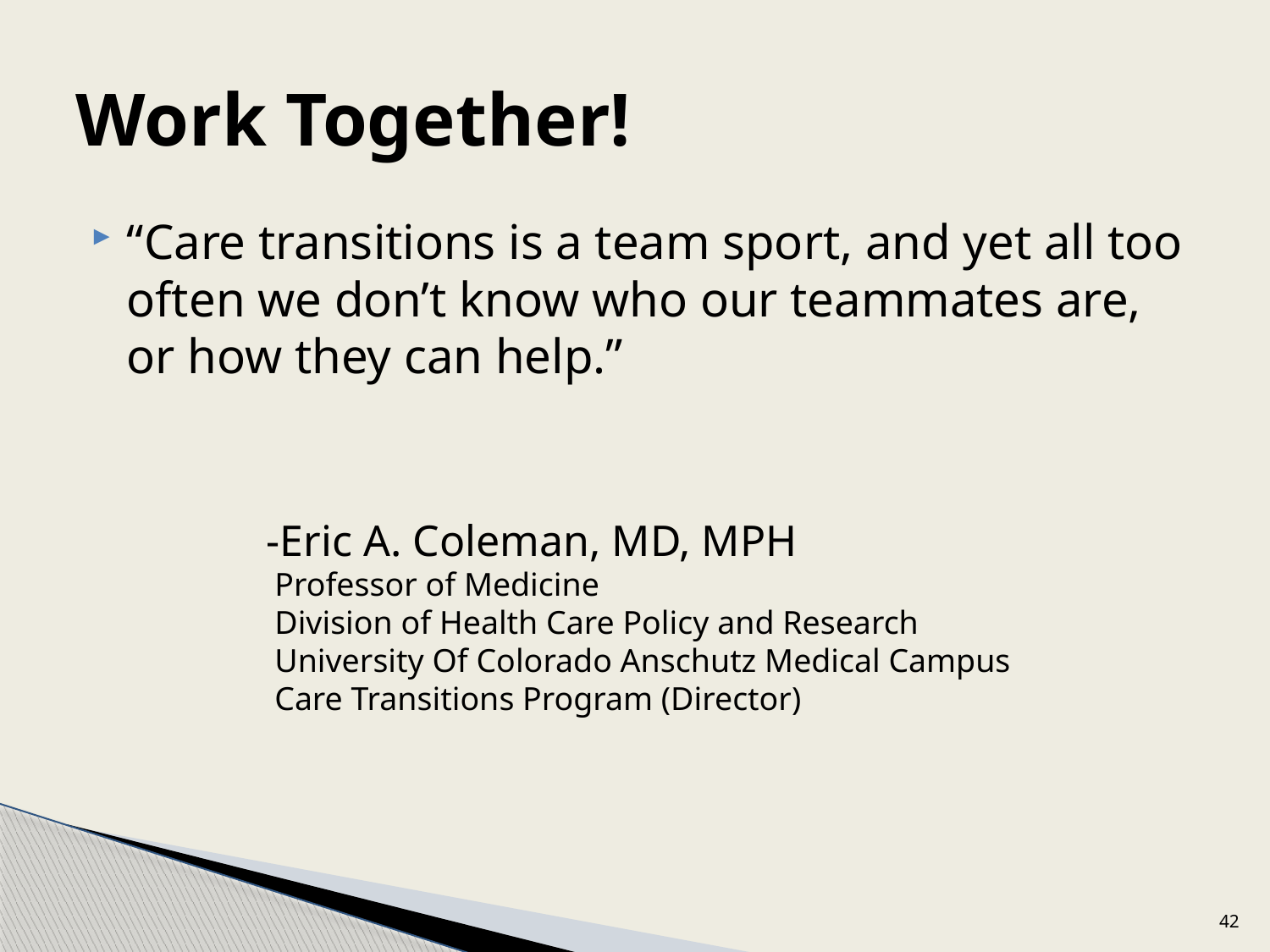

# Work Together!
“Care transitions is a team sport, and yet all too often we don’t know who our teammates are, or how they can help.”
-Eric A. Coleman, MD, MPH
 Professor of Medicine
 Division of Health Care Policy and Research
 University Of Colorado Anschutz Medical Campus
 Care Transitions Program (Director)
42

## Slide 43
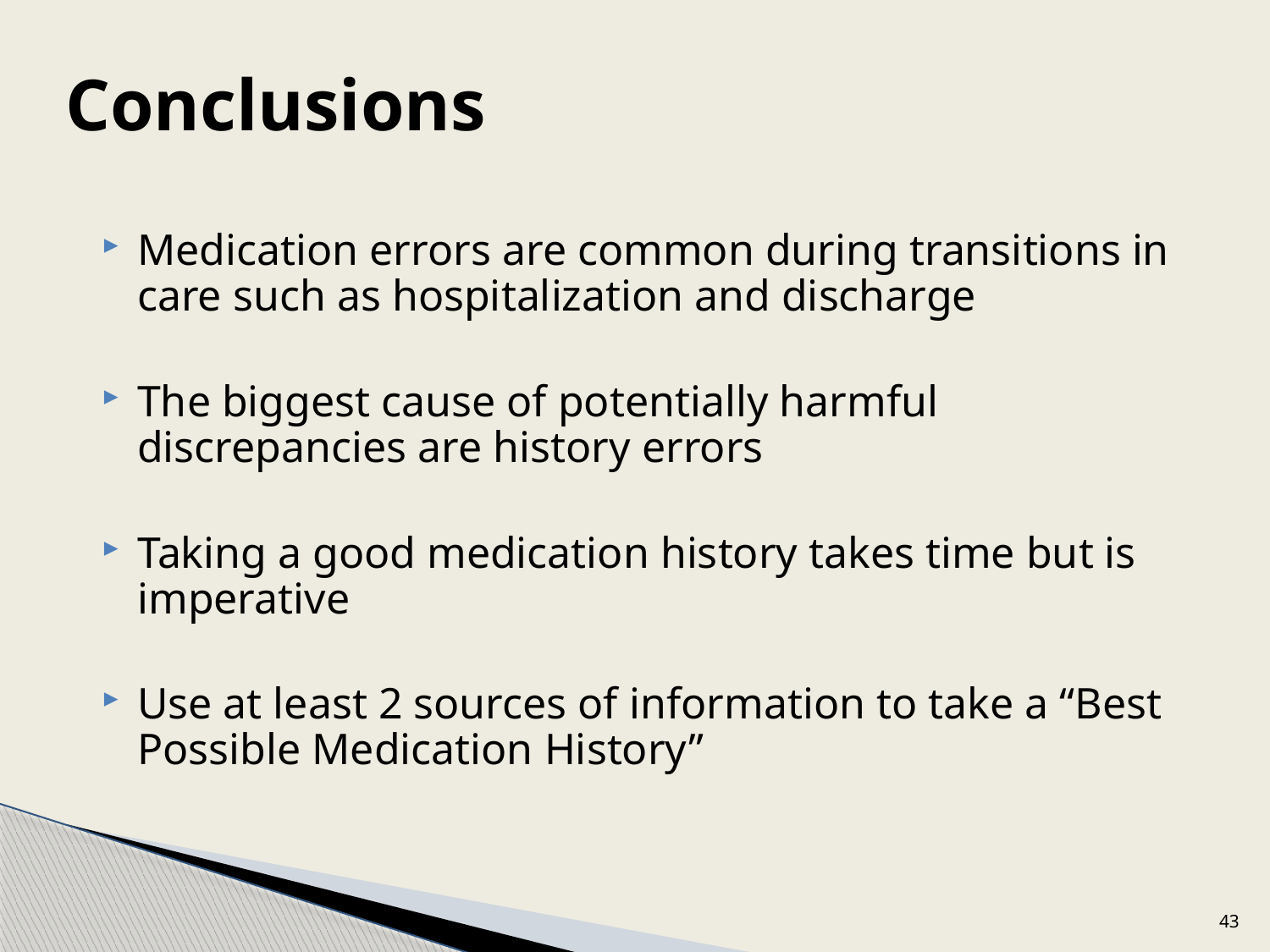

# Conclusions
Medication errors are common during transitions in care such as hospitalization and discharge
The biggest cause of potentially harmful discrepancies are history errors
Taking a good medication history takes time but is imperative
Use at least 2 sources of information to take a “Best Possible Medication History”
43

## Slide 44
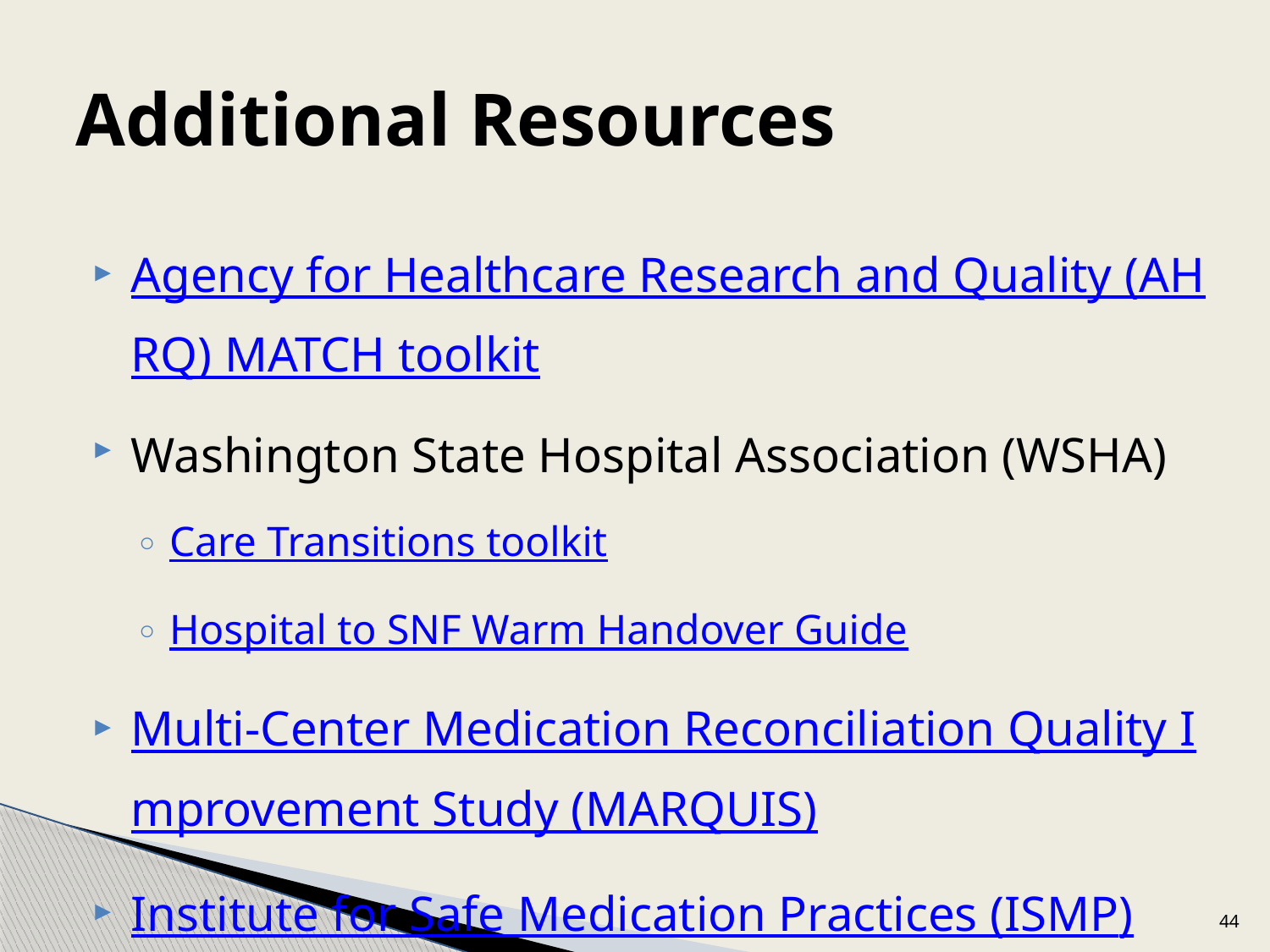

# Additional Resources
Agency for Healthcare Research and Quality (AHRQ) MATCH toolkit
Washington State Hospital Association (WSHA)
Care Transitions toolkit
Hospital to SNF Warm Handover Guide
Multi-Center Medication Reconciliation Quality Improvement Study (MARQUIS)
Institute for Safe Medication Practices (ISMP)
44

## Slide 45
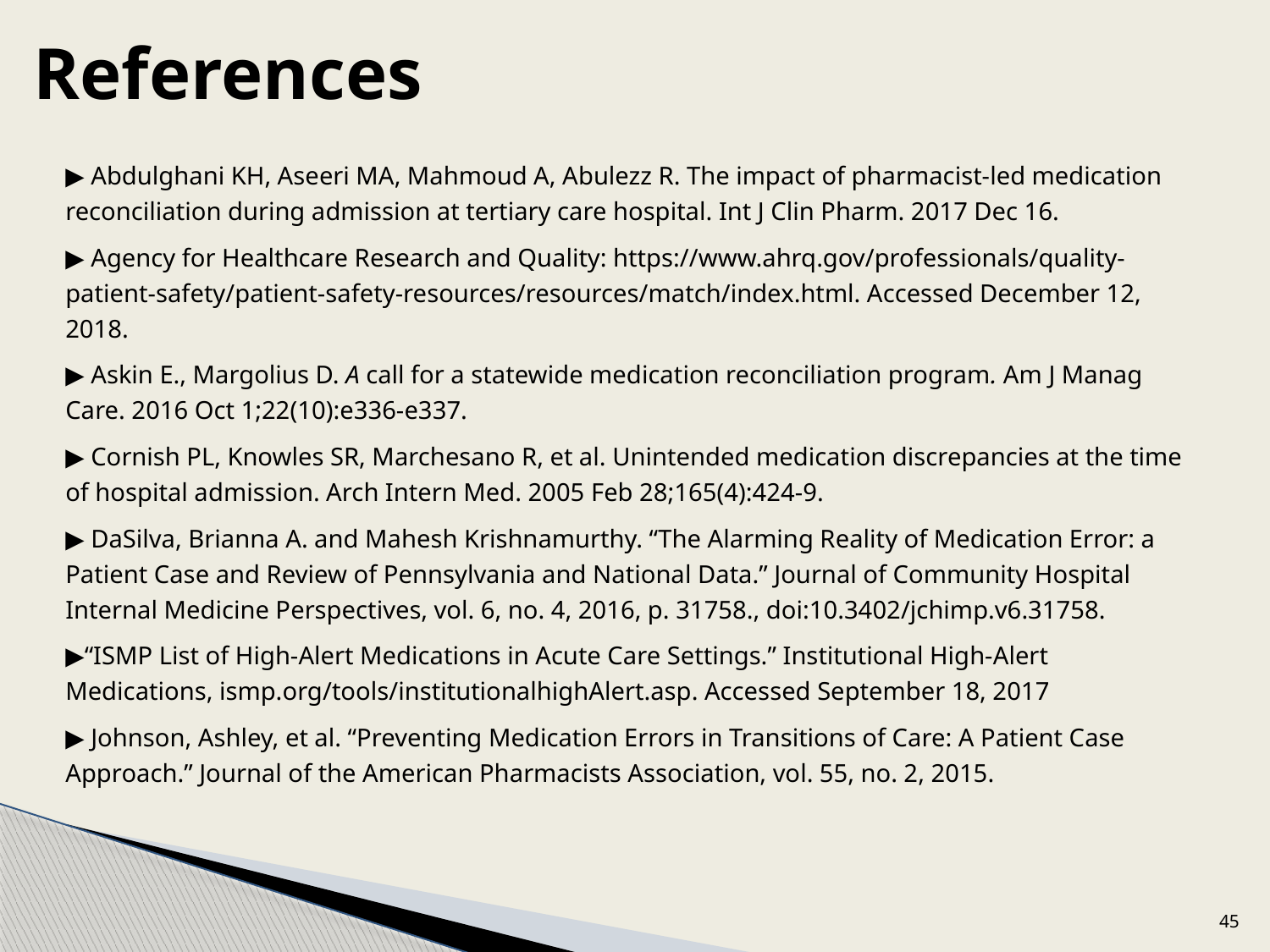

# References
▶ Abdulghani KH, Aseeri MA, Mahmoud A, Abulezz R. The impact of pharmacist-led medication reconciliation during admission at tertiary care hospital. Int J Clin Pharm. 2017 Dec 16.
▶ Agency for Healthcare Research and Quality: https://www.ahrq.gov/professionals/quality-patient-safety/patient-safety-resources/resources/match/index.html. Accessed December 12, 2018.
▶ Askin E., Margolius D. A call for a statewide medication reconciliation program. Am J Manag Care. 2016 Oct 1;22(10):e336-e337.
▶ Cornish PL, Knowles SR, Marchesano R, et al. Unintended medication discrepancies at the time of hospital admission. Arch Intern Med. 2005 Feb 28;165(4):424-9.
▶ DaSilva, Brianna A. and Mahesh Krishnamurthy. “The Alarming Reality of Medication Error: a Patient Case and Review of Pennsylvania and National Data.” Journal of Community Hospital Internal Medicine Perspectives, vol. 6, no. 4, 2016, p. 31758., doi:10.3402/jchimp.v6.31758.
▶“ISMP List of High-Alert Medications in Acute Care Settings.” Institutional High-Alert Medications, ismp.org/tools/institutionalhighAlert.asp. Accessed September 18, 2017
▶ Johnson, Ashley, et al. “Preventing Medication Errors in Transitions of Care: A Patient Case Approach.” Journal of the American Pharmacists Association, vol. 55, no. 2, 2015.
45

## Slide 46
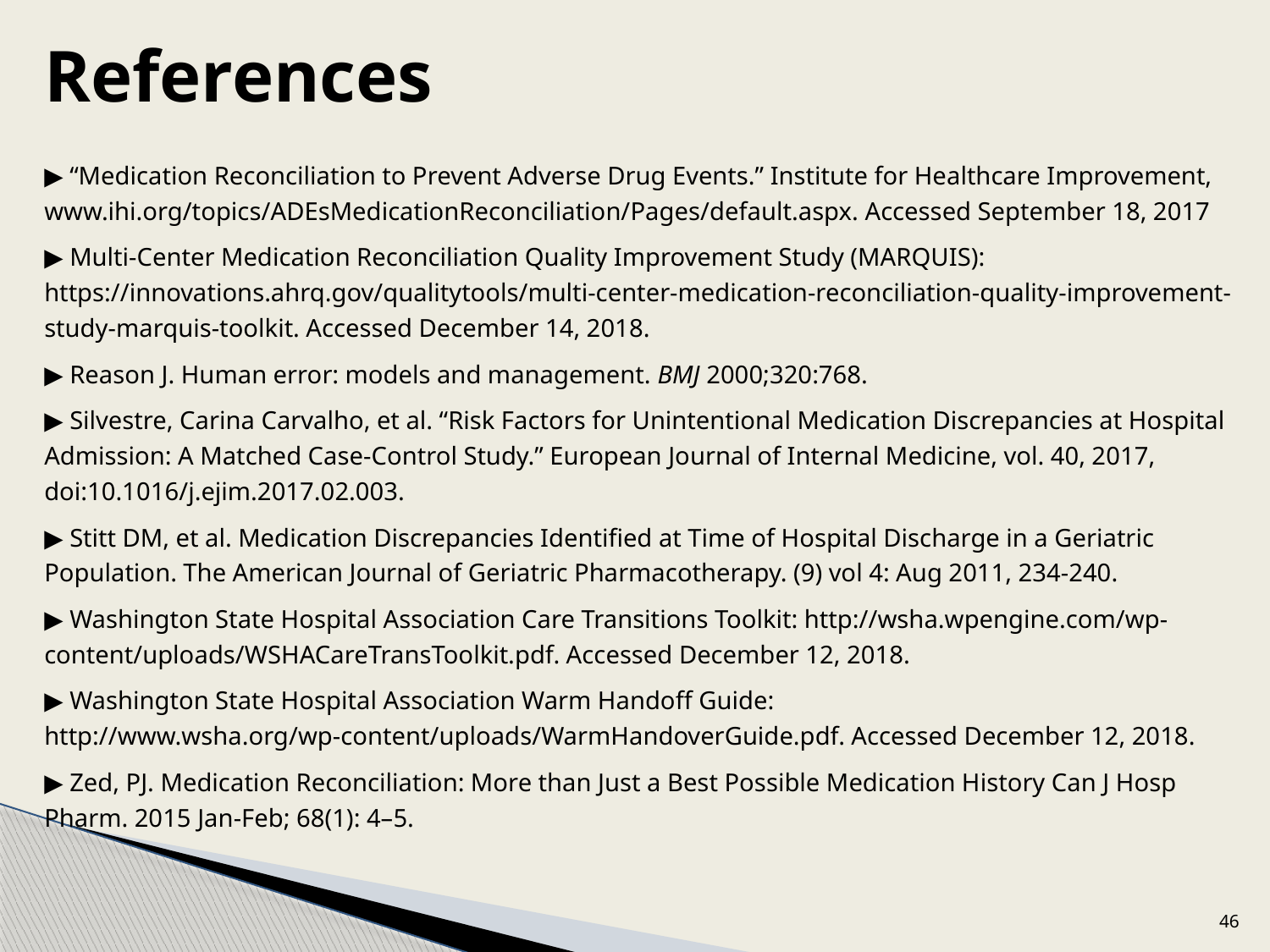

# References
▶ “Medication Reconciliation to Prevent Adverse Drug Events.” Institute for Healthcare Improvement, www.ihi.org/topics/ADEsMedicationReconciliation/Pages/default.aspx. Accessed September 18, 2017
▶ Multi-Center Medication Reconciliation Quality Improvement Study (MARQUIS): https://innovations.ahrq.gov/qualitytools/multi-center-medication-reconciliation-quality-improvement-study-marquis-toolkit. Accessed December 14, 2018.
▶ Reason J. Human error: models and management. BMJ 2000;320:768.
▶ Silvestre, Carina Carvalho, et al. “Risk Factors for Unintentional Medication Discrepancies at Hospital Admission: A Matched Case-Control Study.” European Journal of Internal Medicine, vol. 40, 2017, doi:10.1016/j.ejim.2017.02.003.
▶ Stitt DM, et al. Medication Discrepancies Identified at Time of Hospital Discharge in a Geriatric Population. The American Journal of Geriatric Pharmacotherapy. (9) vol 4: Aug 2011, 234-240.
▶ Washington State Hospital Association Care Transitions Toolkit: http://wsha.wpengine.com/wp-content/uploads/WSHACareTransToolkit.pdf. Accessed December 12, 2018.
▶ Washington State Hospital Association Warm Handoff Guide: http://www.wsha.org/wp-content/uploads/WarmHandoverGuide.pdf. Accessed December 12, 2018.
▶ Zed, PJ. Medication Reconciliation: More than Just a Best Possible Medication History Can J Hosp Pharm. 2015 Jan-Feb; 68(1): 4–5.
46
