# Supplementary material for: Medication Reconciliation: An Educational Module
Source: MedEdPORTAL. 2019 Nov 1;15:10852. doi: 10.15766/mep_2374-8265.10852 (PMC6952281; doi:10.15766/mep_2374-8265.10852)
Supplement: Supplementary file 1 — A. Medication Reconciliation Slides.pptx B. Embedded ARS Questions.docx C. Pre-Post Assessment.docx D. Pre-Post Assessment Answers and References.docx [file mep-15-10852-s001.zip › B. Embedded ARS Questions.docx]

**Appendix B: Optional Embedded Audience Response Self-efficacy Questions**

*You can use a program like Poll Everywhere® or another “audience response system.” Please adjust the “select your current position” based on your audience.*

**At the beginning of the program:**

***Please select your current position***

1. *Student (Medical, PA)*
2. *Resident*
3. *Fellow*
4. *PA/NP*
5. *Attending*
6. *Nurse*
7. *Pharmacist*

***To what extent are you confident in your ability to do an appropriate medication reconciliation on admission TO the hospital?***

1. *Not at all confident*
2. *Somewhat confident*
3. *Neutral*
4. *Confident*
5. *Extremely confident*

***To what extent are you confident in your ability to do an appropriate medication reconciliation on discharge from hospital TO home or rehabilitation?***

1. *Not at all confident*
2. *Somewhat confident*
3. *Neutral*
4. *Confident*
5. *Extremely confident*

**At the conclusion of your program:**

***Rate how likely this presentation will improve your future medication reconciliation efforts:***

1. *Extremely unlikely*
2. *Unlikely*
3. *Neutral*
4. *Likely*
5. *Extremely likely*
