# Supplementary material for: Medication Reconciliation: An Educational Module
Source: MedEdPORTAL. 2019 Nov 1;15:10852. doi: 10.15766/mep_2374-8265.10852 (PMC6952281; doi:10.15766/mep_2374-8265.10852)
Supplement: Supplementary file 1 — A. Medication Reconciliation Slides.pptx B. Embedded ARS Questions.docx C. Pre-Post Assessment.docx D. Pre-Post Assessment Answers and References.docx [file mep-15-10852-s001.zip › C. Pre-Post Assessment.docx]

1. **Which of the following can be used as a source for medication history?**
   1. Patient/caregiver via interview or review of written list
   2. Pharmacy/Pharmacies where medications filled or review of medication bottles
   3. Medication list from outpatient providers
   4. Discharge medication list from recent hospitalizations or other facilities
   5. All of the above
2. **Which techniques should NOT be used to get the “best possible medication history”?**
   1. Ask open-ended questions about medication list
   2. Return to review new information and resolve remaining discrepancies
   3. Verify the list by reading your copy aloud
   4. Try to use two or more sources of information
3. **Which of the following should be included in an accurate medication list for a particular patient?**
4. Drug name, dosage, last time taken, name of prescribing physician
5. Drug name, dosage, frequency of taking medication, and route
6. Drug name, frequency, when medication was started, last time medication was taken
7. Drug name, dosage, name of prescribing physician, and adverse effects
8. **Of all medication errors that occur during transitions of care, medication reconciliation errors account for what percentage?**
9. 80%
10. 70%
11. 60%
12. 40%
13. **Barriers to good medication reconciliation include all of the following EXCEPT:**
    1. Time constraints
    2. Multiple medical providers
    3. Short medication lists
    4. Speaking different language than patient
    5. Cognitive impairment
14. **Which of the following is NOT a high risk for medication errors?**
15. Insurance companies
16. Limited access to health care
17. Low socioeconomic status
18. Language barrier
19. **Which of the following is *correct* about medication discrepancies?**
    1. Error of commission is when a medication is mistakenly omitted (left off) from medication list
    2. A schedule error is when a list is missing duration of therapy (eg antibiotic)
    3. A duplicate drug class error is when patient is discharged on a medication that is contraindicated for his/her condition
    4. I-STOP can be used to reconcile all medications
20. **Which of the following is not included in The Joint Commission’s process for discharge medications?**
    1. Develop a list of current medications
    2. Develop a list of medications to be prescribed
    3. Compare the medications on the two lists
    4. Make clinical decisions based on the comparison
    5. Verify insurance coverage
    6. Communicate the new list to appropriate caregivers and to the patient
21. **How important is accurate medication reconciliation in caring for hospitalized patients?**
    1. Not at all important
    2. Slightly important
    3. Neutral
    4. Moderately important
    5. Extremely important
22. **Please circle your current position/title:**

PA Student

Medical Student

Resident

Fellow

PA/NP

Attending
