# Supplementary material for: Medication Reconciliation: An Educational Module
Source: MedEdPORTAL. 2019 Nov 1;15:10852. doi: 10.15766/mep_2374-8265.10852 (PMC6952281; doi:10.15766/mep_2374-8265.10852)
Supplement: Supplementary file 1 — A. Medication Reconciliation Slides.pptx B. Embedded ARS Questions.docx C. Pre-Post Assessment.docx D. Pre-Post Assessment Answers and References.docx [file mep-15-10852-s001.zip › D. Pre-Post Assessment Answers and References.docx]

1. **Which of the following can be used as a source for medication history?**
   1. Patient/caregiver via interview or review of written list
   2. Pharmacy/Pharmacies where medications filled or review of medication bottles
   3. Medication list from outpatient providers
   4. Discharge medication list from recent hospitalizations or other facilities
   5. All of the above

Answer: e – all of the above are useful sources for medication history

Cornish PL, [Knowles SR](https://www.ncbi.nlm.nih.gov/pubmed/?term=Knowles%20SR%5BAuthor%5D&cauthor=true&cauthor_uid=15738372), [Marchesano R](https://www.ncbi.nlm.nih.gov/pubmed/?term=Marchesano%20R%5BAuthor%5D&cauthor=true&cauthor_uid=15738372), et al.

Unintended medication discrepancies at the time of hospital admission. [Arch Intern Med.](https://www.ncbi.nlm.nih.gov/pubmed/15738372) 2005 Feb 28;165(4):424-9.

Zed, PJ. Medication Reconciliation: More than Just a Best Possible Medication History C[an J Hosp Pharm](https://www.ncbi.nlm.nih.gov/pmc/articles/PMC4350498/). 2015 Jan-Feb; 68(1): 4–5.

1. **Which techniques should NOT be used to get the “best possible medication history”?**
   1. Ask open-ended questions about medication list
   2. Return to review new information and resolve remaining discrepancies
   3. Verify the list by reading your copy aloud
   4. Try to use two or more sources of information

Answer: c – providers should avoid obtaining the current best possible medication history via verifying a written list because it is less reliable than other methods

Cornish PL, [Knowles SR](https://www.ncbi.nlm.nih.gov/pubmed/?term=Knowles%20SR%5BAuthor%5D&cauthor=true&cauthor_uid=15738372), [Marchesano R](https://www.ncbi.nlm.nih.gov/pubmed/?term=Marchesano%20R%5BAuthor%5D&cauthor=true&cauthor_uid=15738372), et al.

Unintended medication discrepancies at the time of hospital admission. [Arch Intern Med.](https://www.ncbi.nlm.nih.gov/pubmed/15738372) 2005 Feb 28;165(4):424-9.

Zed, PJ. Medication Reconciliation: More than Just a Best Possible Medication History C[an J Hosp Pharm](https://www.ncbi.nlm.nih.gov/pmc/articles/PMC4350498/). 2015 Jan-Feb; 68(1): 4–5.

1. **Which of the following should be included in an accurate medication list for a particular patient?**
2. Drug name, dosage, last time taken, name of prescribing physician
3. Drug name, dosage, frequency of taking medication, and route
4. Drug name, frequency, when medication was started, last time medication was taken
5. Drug name, dosage, name of prescribing physician, and adverse effects

Answer: b – though knowing prescribing physician, adverse effects, when medication was started, and when it was last taken can be useful information, they are not the key components of the accurate active medication list (as listed in b).

“Medication Reconciliation to Prevent Adverse Drug Events.” Institute for Healthcare Improvement, www.ihi.org/topics/ADEsMedicationReconciliation/Pages/default.aspx. Accessed September 18, 2017

1. **Of all medication errors that occur during transitions of care, medication reconciliation errors account for what percentage?**
   1. 80%
   2. 70%
   3. 60%
   4. 40%

Answer: c –60% of all medication reconciliation errors occur during transitions of care

Askin E., Margolius D. *A call for a statewide medication reconciliation program.* Am J Manag Care. 2016 Oct 1;22(10):e336-e337.

1. **Barriers to good medication reconciliation include all of the following EXCEPT:**
   1. Time constraints
   2. Multiple medical providers
   3. Short medication lists
   4. Speaking different language than patient
   5. Cognitive impairment

Answer: c – short medication lists are not a barrier to good medication reconciliation as they are less time consuming so that reduces the burden

[Abdulghani KH](https://www.ncbi.nlm.nih.gov/pubmed/?term=Abdulghani%20KH%5BAuthor%5D&cauthor=true&cauthor_uid=29248986), [Aseeri MA](https://www.ncbi.nlm.nih.gov/pubmed/?term=Aseeri%20MA%5BAuthor%5D&cauthor=true&cauthor_uid=29248986), [Mahmoud A](https://www.ncbi.nlm.nih.gov/pubmed/?term=Mahmoud%20A%5BAuthor%5D&cauthor=true&cauthor_uid=29248986), [Abulezz R](https://www.ncbi.nlm.nih.gov/pubmed/?term=Abulezz%20R%5BAuthor%5D&cauthor=true&cauthor_uid=29248986). The impact of pharmacist-led medication reconciliation during admission at tertiary care hospital. Int J Clin Pharm. 2017 Dec 16, Epub.

1. **Which of the following is NOT a high risk for medication errors?**
2. Insurance companies
3. Limited access to health care
4. Low socioeconomic status
5. Language barrier

Answer: a – insurance companies are not a risk factor for medication errors. However, limited access to health care, low socioeconomic status, and language barriers indeed increase risk for medication errors though lack of resources and lack of understanding.

Silvestre, Carina Carvalho, et al. “Risk Factors for Unintentional Medication Discrepancies at Hospital Admission: A Matched Case-Control Study.” European Journal of Internal Medicine, vol. 40, 2017, Epub.

1. **Which of the following is *correct* about medication discrepancies?**
   1. Error of commission is when a medication is mistakenly omitted (left off) from medication list
   2. A schedule error is when a list is missing duration of therapy (eg antibiotic)
   3. A duplicate drug class error is when patient is discharged on a medication that is contraindicated for his/her condition
   4. I-STOP can be used to reconcile all medications

Answer: b – Schedule errors include incorrect or missing duration of therapy, dose or frequency of a medication. Error of omission is when a medication is mistakenly omitted from a list of documented medication. Error of commission is when a medication is mistakenly included in a list of documented medications. I-STOP is a valuable reconciliation tool but only lists controlled substances.

“Medication Reconciliation to Prevent Adverse Drug Events.” Institute for Healthcare Improvement, www.ihi.org/topics/ADEsMedicationReconciliation/Pages/default.aspx. Accessed September 18, 2017

1. **Which of the following is not included in The Joint Commission’s process for discharge medications?**
   1. Develop a list of current medications
   2. Develop a list of medications to be prescribed
   3. Compare the medications on the two lists
   4. Make clinical decisions based on the comparison
   5. Verify insurance coverage
   6. Communicate the new list to appropriate caregivers and to the patient

Answer e: Though for some medications (expensive or generally non-formulary), it is beneficial to verify insurance coverage prior to discharge, this action is not included in the Joint Commission’s process for discharge medications.

Johnson, Ashley, et al. “Preventing Medication Errors in Transitions of Care: A Patient Case Approach.” Journal of the American Pharmacists Association, vol. 55, no. 2, 2015, doi:10.1331/japha.2015.15509.

1. **How important is accurate medication reconciliation in caring for hospitalized patients?**
   1. Not at all important
   2. Slightly important
   3. Neutral
   4. Moderately important
   5. Extremely important
2. **Please circle your current position/title:**

PA Student

Medical Student

Resident

Fellow

PA/NP

Attending
